# Moderate prevalence and evolutionary causes of paralogous compensation in humans

**DOI:** 10.1093/nsr/nwag565

**Published:** 2026-09-03

**Authors:** Jieyu Shen, Daqi Yu, Wenjia Wang, Tianhan Su, Dingding Wang, Junbo Liang, Yiqiong Guo, Shangqi Zhao, Huijing Ma, Jichang Wang, Xiaoyue Wang, Yong E Zhang

**Affiliations:** State Key Laboratory of Animal Biodiversity Conservation and Integrated Pest Management, Institute of Zoology, Chinese Academy of Sciences, Beijing 100101, China; University of Chinese Academy of Sciences, Beijing 100049, China; State Key Laboratory of Animal Biodiversity Conservation and Integrated Pest Management, Institute of Zoology, Chinese Academy of Sciences, Beijing 100101, China; University of Chinese Academy of Sciences, Beijing 100049, China; State Key Laboratory of Common Mechanism Research for Major Diseases, Department of Biochemistry and Molecular Biology, Institute of Basic Medical Sciences & School of Basic Medicine, Chinese Academy of Medical Sciences & Peking Union Medical College, Beijing 100005, China; State Key Laboratory of Animal Biodiversity Conservation and Integrated Pest Management, Institute of Zoology, Chinese Academy of Sciences, Beijing 100101, China; University of Chinese Academy of Sciences, Beijing 100049, China; State Key Laboratory of Common Mechanism Research for Major Diseases, Department of Biochemistry and Molecular Biology, Institute of Basic Medical Sciences & School of Basic Medicine, Chinese Academy of Medical Sciences & Peking Union Medical College, Beijing 100005, China; State Key Laboratory of Common Mechanism Research for Major Diseases, Department of Biochemistry and Molecular Biology, Institute of Basic Medical Sciences & School of Basic Medicine, Chinese Academy of Medical Sciences & Peking Union Medical College, Beijing 100005, China; State Key Laboratory of Complex, Severe, and Rare Diseases, Center for Bioinformatics, National Infrastructures for Translational Medicine, Institute of Clinical Medicine, Peking Union Medical College Hospital, Chinese Academy of Medical Sciences & Peking Union Medical College, Beijing 100730, China; State Key Laboratory of Complex, Severe, and Rare Diseases, Center for Bioinformatics, National Infrastructures for Translational Medicine, Institute of Clinical Medicine, Peking Union Medical College Hospital, Chinese Academy of Medical Sciences & Peking Union Medical College, Beijing 100730, China; State Key Laboratory of Animal Biodiversity Conservation and Integrated Pest Management, Institute of Zoology, Chinese Academy of Sciences, Beijing 100101, China; Key Laboratory for Stem Cells and Tissue Engineering (Sun Yat-sen University), Ministry of Education, Guangzhou 510080, China; Department of Histology and Embryology, Zhongshan School of Medicine, Sun Yat-sen University, Guangzhou 510080, China; State Key Laboratory of Complex, Severe, and Rare Diseases, Center for Bioinformatics, National Infrastructures for Translational Medicine, Institute of Clinical Medicine, Peking Union Medical College Hospital, Chinese Academy of Medical Sciences & Peking Union Medical College, Beijing 100730, China; State Key Laboratory of Animal Biodiversity Conservation and Integrated Pest Management, Institute of Zoology, Chinese Academy of Sciences, Beijing 100101, China; University of Chinese Academy of Sciences, Beijing 100049, China

**Keywords:** gene duplication, paralogous compensation, genetic robustness, CRISPR screening, pleiotropy, retrogene

## Abstract

Paralogous compensation drives genetic robustness; however, its *in vivo* prevalence in humans and the evolutionary forces shaping it remain unresolved. We CRISPR-screened 678 human paralog pairs to identify pairs with negative genetic interactions (nGIs) or compensatory paralogs, and integrated our results with four studies to assemble two complementary resources: the 2168-pair Union and 4859-pair Ito datasets. We extrapolated to the organismal level by classifying paralogs as core (broadly compensating, 99 Union/120 Ito pairs), conditional (111/566) or non-interacting (1958/4173), and testing for enriched compensatory signals in core pairs relative to the latter two groups. Core nGI pairs consistently showed prenatal co-expression, depletion of disease genes and enrichment for upregulation after paralog loss in cancer, though the signals were often moderate. Evolutionary analyses found similar fractions (∼2%) of core compensators among both ancient and young paralogs. Ancient pairs retain overlaps likely under pleiotropic and dosage constraints. Incomplete small-scale duplication mechanisms limit compensation among young pairs, except X-derived retrogenes, which are overrepresented among core nGIs (∼14%). This is consistent with selection to counteract male germline X inactivation and subsequent out-of-testis expansion. Overall, paralogous compensation is moderately prevalent *in vivo* in humans, shaped by multiple evolutionary forces acting on ancient or young duplicates.

## INTRODUCTION

For a century, it has been widely accepted that gene duplication fuels evolution by supplying raw material for new genes and functions [1–8]. Classical models posit that duplicates diverge via neofunctionalization or subfunctionalization, with one copy acquiring a novel role or ancestral functions being partitioned between copies [2–5,7,9–11]. An expression-specialization model arising from sub- or neofunctionalization describes cases in which one paralog evolves a restricted expression pattern while the other retains broader expression [12–14]. Nevertheless, most duplicates actually undergo nonfunctionalization and are lost or pseudogenized within a few million years [15,16]. Across these models, functional redundancy is expected to be uncommon or largely confined to evolutionarily young paralogs.

Since the 1990s, extensive efforts have refined this traditional view where functional overlap (especially partial overlap) could be stably maintained [5,17–23]. On the one hand, a version of sub/neofunctionalization model, i.e. the pleiotropy model, posits that if the shared, redundant function of a paralog is intrinsically linked to its other, non-redundant functions, then selection will preserve this overlap indefinitely [17,18,24]. A refined version of this concept, the entanglement model, further suggests that interdependent or entangled functional domains within

paralogous proteins can lead to correlated evolutionary constraints, thereby locking in their shared roles [19,25]. On the other hand, a version of the subfunctionalization model, i.e. the dosage subfunctionalization model, proposes that natural selection can act to maintain an optimal, combined expression level from both paralogs, effectively preserving a state of mutual dependency [20–22,26]. Consistently, large-scale digenic perturbation screens in yeast and worms have demonstrated that a substantial number of ancient paralog pairs, some separated by over 100 million years of evolution, still exhibit negative genetic interactions (nGIs, a hallmark of functional compensation [24,27–32]). A pioneering study in yeast directly showed the correlated evolution signal of paralogs, supporting the entanglement or pleiotropy model [19,25]. Thus, shaped by pleiotropy and dosage constraints, paralogous compensation may be more pervasive than anticipated, enhancing genetic robustness [19,24,25].

Due to technical challenges, the understanding of paralogous compensation has lagged in most species, including humans. Researchers relied on a limited number of experimental case studies, or on indirect computational inferences, such as associating a gene’s essentiality with the loss of its paralog [33–36]. Similarly, large-scale organismal homozygous knockout studies in mice suggest the presence of potential paralogous compensation: non-essential genes are more likely to have paralogs than essential genes [37]. However, this inference may be confounded by the possibility that non-essential genes are themselves more duplicable [34]. Since 2020, the advent of combinatorial CRISPR-based screening technologies has recently enabled high-throughput experimental interrogation of GIs in human cell lines, opening a new window into this question [38–43]. These studies, often motivated by the search for synthetic lethal interactions for cancer therapy [44,45], have begun to map the landscape of paralogous dependencies. Despite this progress, three critical gaps in humans remain. First, the vast combinatorial space of over 80 000 paralog pairs is largely unexplored, with existing screens only covering hundreds to thousands of pairs. Second, our knowledge is almost exclusively derived from *in vitro* cell line models, leaving the extent and nature of compensation at the organismal level (for instance, during development or in the context of disease) as a major open question. Finally, the evolutionary forces that have shaped paralogous compensation are still poorly understood.

To address these fundamental questions, we conducted a study that integrates a newly generated, purpose-built CRISPR library with existing large-scale datasets. By analyzing this extensive resource, we investigated the prevalence of paralogous compensation across diverse human cell lines, inferred its implications in development and cancer, and dissected the distinct evolutionary forces that underlie the functional overlap of both young and ancient duplicate genes (Fig. 1a).

**Figure 1.**
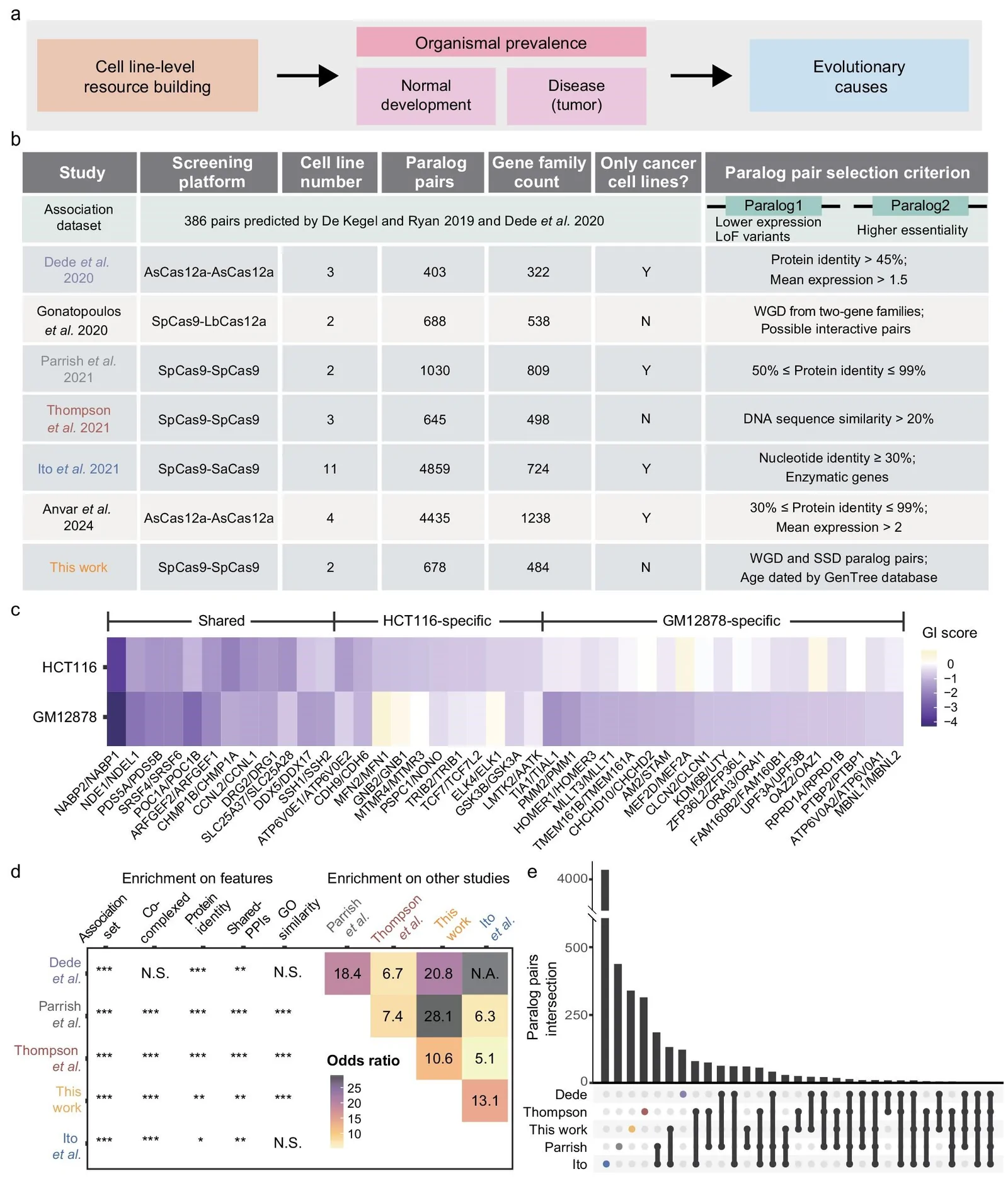
Overview and quality control of dual CRISPR knockout datasets for paralog pairs. (a) The overall design of this study. (b) Summary of CRISPR screens targeting paralog pairs. The ‘association dataset’ comprises predicted negative GI pairs (Materials and Methods). Five datasets (color-coded) were used downstream. ‘LoF’ represents loss-of-function. (c) Identification of 42 negative GI pairs in HCT116 or GM12878 cells. Smaller negative GI scores indicate greater-than-additive fitness loss upon double knockout. (d) Enrichment of negative GI pairs by molecular features and cross-study overlap. Features include membership in the association dataset, co-complex status, protein sequence identity, shared PPIs and functional annotation similarity. Odds ratio (OR) denotes enrichment relative to baseline. Significance was assessed by binomial, one-sided Fisher’s exact tests, or Wilcoxon rank-sum tests, as appropriate. *, *P* < 0.05; **, *P* < 0.01; ***, *P* < 0.001; N.S., not significant. (e) Overlap of screened paralog pairs across the five datasets. Two pairs are considered the same if they contain the same two paralogs.

## RESULTS

### Our purpose-built library matches the quality of existing datasets while providing complementary coverage

While the aforementioned previous studies have provided invaluable data, they have often focused on some gene families, employed specific selection criteria of paralog pairs or tumor-derived cell lines (Fig. 1b, Fig. S1a and Table S1). To generate a resource tailored for evolutionary studies, we developed a CRISPR/Cas9-based digenic screen library targeting 678 paralog pairs (Table S2 and Supplementary Note), characterized by three key features. (1) This library was designed to sample paralogs annotated by origination time and mechanisms (whole‑genome duplication or WGD, small‑scale duplication or SSD). (2) We employed a stringent single guide RNA (sgRNA) design pipeline to differentiate individual paralogs, even those with high sequence similarity (Fig. 1b, Fig. S1a–c and Supplementary Note). (3) The experiment was performed in both a colon cancer cell line (HCT116) and a relatively normal lymphoblastoid cell line (GM12878), covering malignant and normal contexts. Among 678 paralog pairs, our screen identified 42 paralog pairs (6.2%) with negative GIs in either HCT116 or GM12878, indicating functional compensation (Fig. 1c, Table S3 and Supplementary Note). Consistent with the context-dependent nature of negative GIs reported in previous studies [38–43], only 12 pairs showed compensation in both cell lines, while 11 and 19 were specific to HCT116 and GM12878, respectively.

Our screening dataset demonstrates quality comparable to existing datasets, with experimental evaluation parameters largely consistent (e.g. between-replicate correlation: 0.80–0.87 vs. 0.78–0.92; Fig. S1a, Table S2 and Supplementary Note). Computational assessments reveal similar trends (Fig. 1d). From six published digenic paralog screens, we selected four for comparison: smaller-scale studies by Dede *et al.* [40], Parrish *et al.* [39] and Thompson *et al.* [41], and the large-scale dataset from Ito *et al.* [42]. The Gonatopoulos-Pournatzis *et al.* dataset was excluded due to its focus on WGD pairs and potentially relaxed statistical thresholds [38]. Compared to the large-scale Anvar dataset [43], we prioritized the Ito dataset [42] for its broader cell line coverage (Fig. 1b).

We further performed three additional evaluations across Dede *et al.*, Parrish *et al.*, Thompson *et al.*, Ito *et al.* and our dataset (Materials and Methods). First, using a published ‘association dataset’ of 386 paralog pairs predicted to have negative GIs [35,40], we found significant overrepresentation in all five datasets, with odds ratios of 3.0–7.0 (Fig. 1d left panel and Fig. S1d and e). Second, we analyzed four molecular features associated with compensation, co-complex membership, protein sequence identity, shared protein–protein interaction (PPI) partners and Gene Ontology (GO) similarity [27–30]. All datasets showed expected enrichments for at least two features (Fig. 1d left panel, Fig. S1e and Table S1). Third, for paralog pairs shared across studies, we observed strong consistency in negative GI status, with a median odds ratio of 10.6, highlighting the screens’ reliability (Fig. 1d right panel).

Notably, our library adds 340 previously unassayed paralog pairs (Fig. 1e), expanding the pool of experimentally tested pairs. Together with comparable quality and design tailored for evolutionary studies, it provides a valuable complement to existing datasets.

### Cross-cell line analyses reveal moderate paralog compensation and yield two complementary large-scale datasets

With five high-quality datasets, we examined the prevalence of paralogous compensation at the cellular level. Notably, these datasets involve a diverse collection of cell lines, including 17 cell lines from diverse cancer types and 2 relatively normal cell lines (RPE1 and GM12878). They are derived from up to nine distinct tissues (e.g. skin, eye; Fig. 2a). From a transcriptome-based tree of hundreds of cell lines, these 19 lines span roughly half of major branches (Fig. S2a). The screening data revealed five major patterns (Fig. 2b): (1) a consistently moderate nGI proportion, with a median of 3.6% (range: 0.7%–8.0%), exceeding the median of 1.7% estimated from large-scale surveys of random gene pairs in human cell lines [46,47] (Fig. S2b), (2) no substantial difference between cancer and normal cell lines with A375 and MeWo similar to RPE1 in the Thompson *et al.* dataset (4%–6% vs. 6%), and HCT116 similar to GM12878 in our dataset (3% vs. 5%), (3) a lower proportion of negative GIs in the Ito *et al.* dataset compared to the other four datasets, highlighted by two shared cell lines, MeWo and A549 (0.7%–1.9% vs. 4%), (4) predominant detection of nGIs in cell lines in which both paralogs were expressed (70%–90%; Fig. S2c) and (5) increase in the cumulative proportion of paralog pairs exhibiting nGI in at least one cell line with the number of cell lines surveyed, from 2%–5% in a single cell line to 10%–14% across 10 cell lines (Fig. S2d).

**Figure 2.**
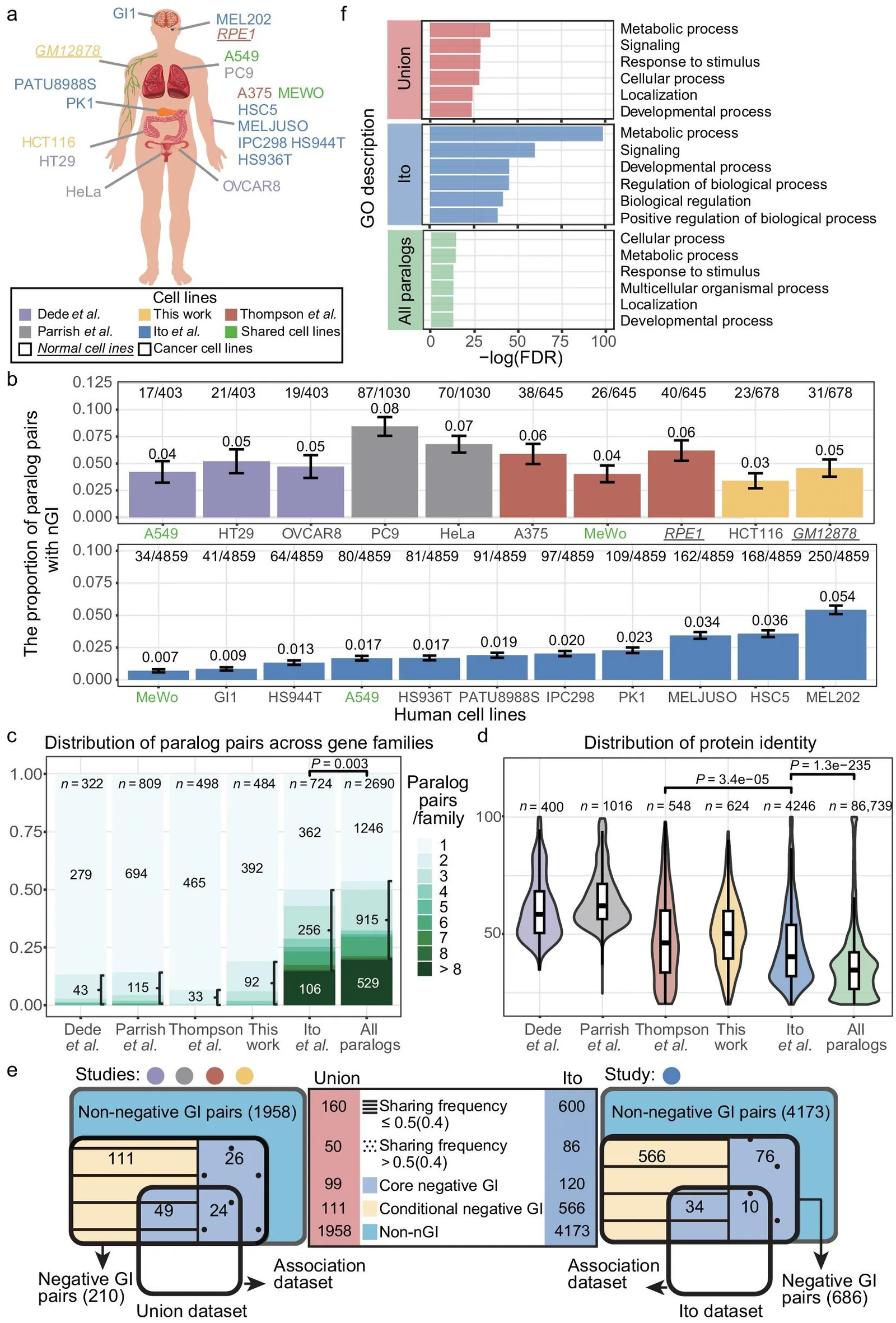
Cell line level paralogous compensation and construction of the Union and Ito datasets. (a) Distribution of the 19 cell lines by organ of origin across the five datasets. The icon source is Freepik (https://www.freepik.com/). (b) Negative GI proportions for individual cell lines across the five studies. Proportions are shown above bars, with error bars representing one standard error (s.e.); counts (negative GI pairs/total library size) are labeled atop each panel. (c and d) Distributions of (c) the number of paralog pairs per screened gene family and (d) paralogous sequence identity, compared with the genome-wide background of all paralog pairs. All studies differ significantly from background, and each of the four smaller datasets differs significantly from the Ito dataset. For clarity, only the two largest *P* values from two-sided Wilcoxon rank-sum tests are reported. For the violin plot in panel (d), the bar indicates the first to third quartiles, and the curve indicates the data density. (e) Classification scheme for core negative GI, conditional negative GI and non-nGI paralog pairs in the Union and Ito datasets. (f) GO enrichment for Union and Ito gene sets vs. the genome-wide duplicate background; the top six enriched GO biological process terms are shown. FDR indicates false discovery rate.

The lower rate in the Ito dataset likely reflects its inclusion of paralogs less prone to compensation, resembling a genome-wide background distribution (Fig. 2c and d). Specifically, Ito sampled more paralogs from large gene families with at least two pairs (50% vs. 7%–24%) representing more thorough paralog combinations within large gene families and included paralogs with lower protein sequence identity (median: 40% vs. 46%–62%). These factors likely reduce digenic compensation rates by increasing high-order paralogous interaction and lowering protein similarity.

To facilitate organism-level analyses while accounting for dataset differences, we compiled the data into two complementary datasets (Materials and Methods): (1) the Union dataset, comprising 2168 pairs from our study and the Dede, Parrish and Thompson screens and (2) the Ito dataset, consisting of 4859 pairs exclusively from Ito *et al*. Paralogs in each dataset were further categorized based on the prevalence of negative GI signals across cell lines (Fig. 2e and Fig. S2e–g). Core nGI pairs exhibited negative GIs in a high proportion of cell lines (sharing frequency >0.5 for Union, >0.4 for Ito), indicative of context-independent functional redundancy. Conditional nGI pairs showed context-specific compensation in fewer cell lines, while non-nGI pairs exhibited no interactions. This classification yielded 99 core, 111 conditional and 1958 non-nGI pairs in the Union dataset, and 120 core, 566 conditional and 4173 non-nGI pairs in the Ito dataset (Table S4).

Beyond evolutionary features such as family size and paralog identity (Fig. 2c and d), the Union and Ito datasets also complement each other in molecular function and duplication type. Union genes reflect all duplicate genes in the human genome, sharing five of the six top GO terms except ‘Signaling’ (Fig. 2f). Conversely, Ito genes, biased toward enzyme families (e.g. kinases [42]), are enriched in biological regulation, with only two GO terms (‘Metabolic process’ and ‘Developmental process’) shared with the genomic background. Furthermore, Union genes maintain a roughly even distribution between WGD- and SSD-derived duplicates (48.4% vs. 51.6%), whereas Ito genes are biased toward SSD-derived duplicates (70.1%; Fig. S2h).

In summary, these analyses corroborate moderate cell line-level paralogous compensation. More importantly, they provide an analytical framework, i.e. core, conditional and non-nGI pairs across two large, complementary datasets, enabling downstream robust and extrapolatory organism-level analyses.

### Core nGI pairs tend to show prenatal co-expression and to be depleted in disease-causing genes

We investigated whether nGIs are relevant to organismal development by testing two hypotheses (Fig. 3a): (1) core or broadly compensatory nGI pairs are more likely to maintain functional overlap during early development, as case studies suggest paralogous compensation in early stages and divergent functions later [48] and (2) loss of a single gene in such pairs is less likely to be associated with severe developmental phenotypes, reflected by their under-representation among developmental disorder genes [49,50].

**Figure 3.**
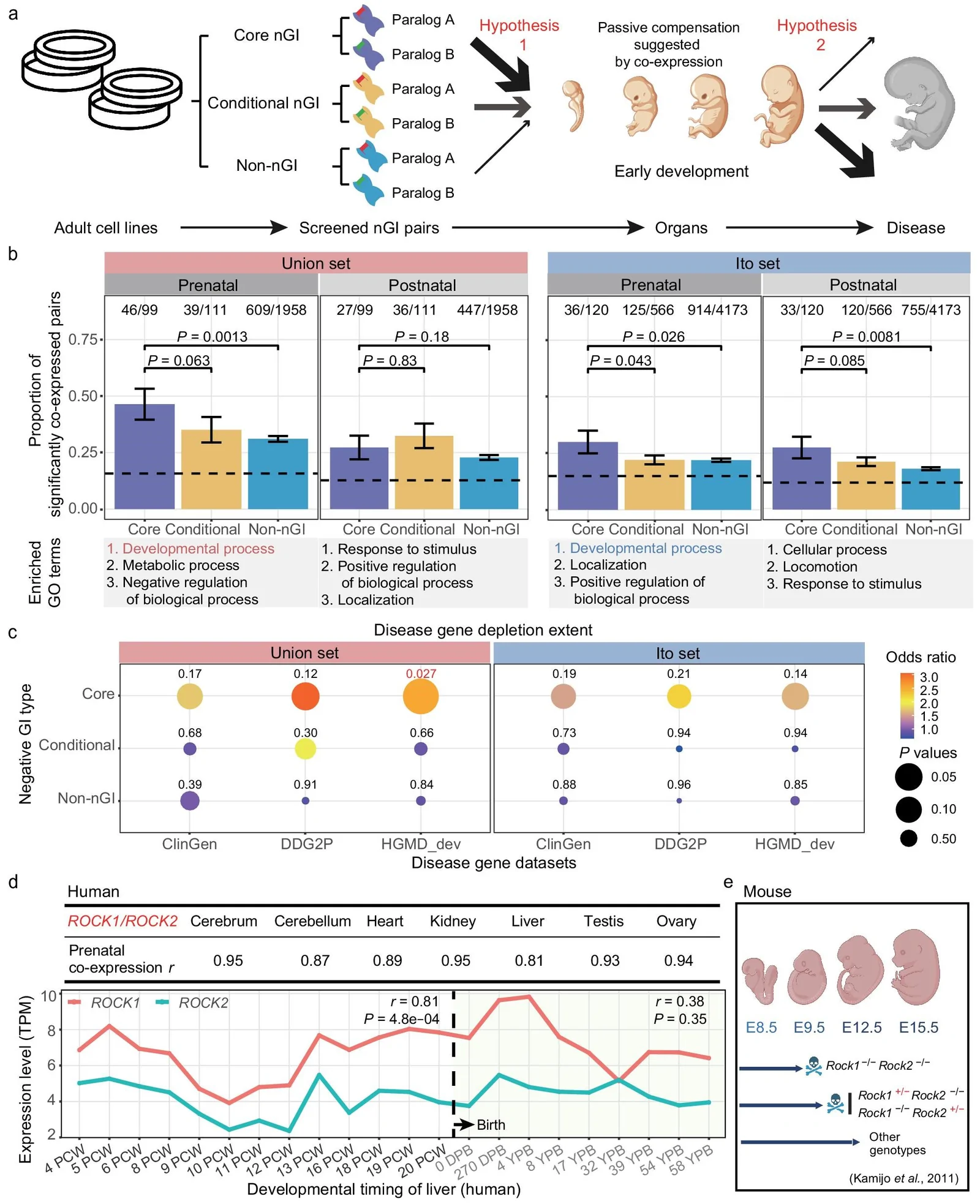
Co-expression and disease gene enrichment analyses across nGI groups. (a) Schematic extrapolating core negative GIs detected in adult cell lines to development and disease. Arrow thickness reflects each group’s relative enrichment for compensatory signals (co-expression) and developmental disorders. Icons: cell plate by Eucalyp (NounProject.com); development by Freepik (https://www.freepik.com/). (b) Proportion of co-expressed paralog pairs in prenatal (left) vs. postnatal stages. Dashed lines indicate randomly shuffled pairs (Materials and Methods). Counts (significantly co-expressed pairs/total pairs) are labeled atop each panel. Group comparisons use one-sided Fisher’s exact tests; error bars denote one s.e. under binomial distribution. Bottom, top three enriched GO biological process terms among significantly co-expressed genes in prenatal and postnatal stages. (c) Depletion status of prenatally co-expressed paralogs among disease-associated genes (ClinGen, DDG2P, HGMD development). Odds ratios compare disease-gene enrichment in non-co-expressed vs. co-expressed pairs; one-sided Fisher’s exact *P* values are shown. (d) Prenatal co-expression of *ROCK1/ROCK2* across organs. Pearson correlations across organs are shown (top). Expression dynamics in human liver are shown (bottom), with prenatal and postnatal periods shaded. Pearson’s *r* and *P* values are reported by stage. PCW, post-conception weeks; DPB, days post birth; YPB, years post birth. (e) Embryonic lethality with loss of *Rock1* and/or *Rock2* in mouse, adapted from Kamijo *et al.* [59]. E, embryonic day.

To test hypothesis 1, we used co-expression, a widely accepted proxy for compensation [21,51–53], and analyzed a developmental transcriptome dataset spanning seven organs from embryonic stages through adulthood [54]. Given the large-scale expression shifts at birth [54], we analyzed prenatal and postnatal stages separately. Two complementary results supported hypothesis 1 (Materials and Methods). First, we computed the median expression correlation for each nGI group within each organ. Consistent with prior studies (e.g. [21]), paralogs showed higher co-expression than random gene pairs (0.11–0.49 vs. −0.01 to 0.05 for Union; 0.06–0.36 vs. −0.02 to 0.04 for Ito; Fig. S3a and Table S5), likely due to the shared regulatory context. More importantly, core nGI pairs showed higher median correlations than other groups in five to six of the seven organs prenatally, a pattern that weakened postnatally. Second, we identified pairs with statistically significant co-expression in at least one organ. All three groups of duplicated gene pairs again exhibited higher co-expression compared to random pairs (Fig. 3b). Among them, core nGI pairs showed the highest proportion of significantly co-expressed pairs during prenatal stages (46.5% for Union, 30.0% for Ito; Fig. 3b), exceeding conditional and non-nGI pairs (one-sided Fisher’s exact test, *P* ≤ 0.063). In contrast, the excess of co-expressed core nGI pairs appears relatively weaker in postnatal stages. Notably, the prenatally co-expressed paralogs were significantly enriched for GO terms related to development (Fig. 3b bottom panel).

To test hypothesis 2, we analyzed three curated human disease gene datasets (Materials and Methods): the Clinical Genome Resource (ClinGen) [55], the Developmental Disorders Gene-to-Phenotype database (DDG2P) [56] and developmental disorder genes from the Human Gene Mutation Database (HGMD_dev) [57,58]. As predicted, compared to non-co-expressed genes, prenatally co-expressed core nGI pairs were depleted among disease genes, with odds ratios ranging from 1.53 to 3.07 (Fig. 3c and Table S6). While only core nGI pairs from the Union dataset showed statistical significance in HGMD_dev (*P* = 0.027), both Union and Ito datasets exhibited similar trends of stronger depletion in two developmental disease gene datasets, i.e. DDG2P and HGMD_dev. In contrast, postnatally co-expressed pairs appeared to show weaker patterns (Fig. S3b).

Notably, prenatal co-expression of core nGI pairs was typically restricted to a few organs, with 67.4% or 50.0% of pairs co-expressed in only one of seven organs (Fig. S3c). The Rho kinases *ROCK1* and *ROCK2* were the only pair co-expressed across all organs. Human prenatal correlation coefficients ranged from 0.81 to 0.95 (Fig. 3d and e) and decreased postnatally, mirroring patterns in mouse development (Fig. 3d and Fig. S3d). Experimental evidence in mice aligns with these findings, as the loss of either *Rock1* or *Rock2* is partially compensated by the other, while their simultaneous loss is embryonically lethal, highlighting their essential and overlapping roles in early development (Fig. 3e [59,60]).

Altogether, core or broadly compensatory paralogs identified in cell lines exhibit heightened co-expression during development and the co-expressed genes appear to be depleted from developmental disorder gene lists. Both patterns appear stronger during prenatal than postnatal stages, suggesting greater potential for paralogous buffering during prenatal development.

### Core nGI pairs exhibit active compensation and mutual exclusivity in cancer, though both are modest

To investigate the role of paralogous compensation in disease, particularly cancer, we analyzed large-scale mutation and expression data from The Cancer Genome Atlas (TCGA [61]) across 32 tumor types (Materials and Methods). Analogous to how we analyzed development, we also hypothesized that functional backup provided by core nGI pairs would be co-opted by cells following the loss of one paralog. Two analytical frameworks were used (Fig. 4a): identifying potentially actively compensatory pairs [62,63], where the copy number (CN) loss of one paralog (paralog 1) is associated with significant transcriptional upregulation of its partner (paralog 2), and mutually exclusive pairs, where the simultaneous loss of both paralogs occurs less frequently than expected by chance.

**Figure 4.**
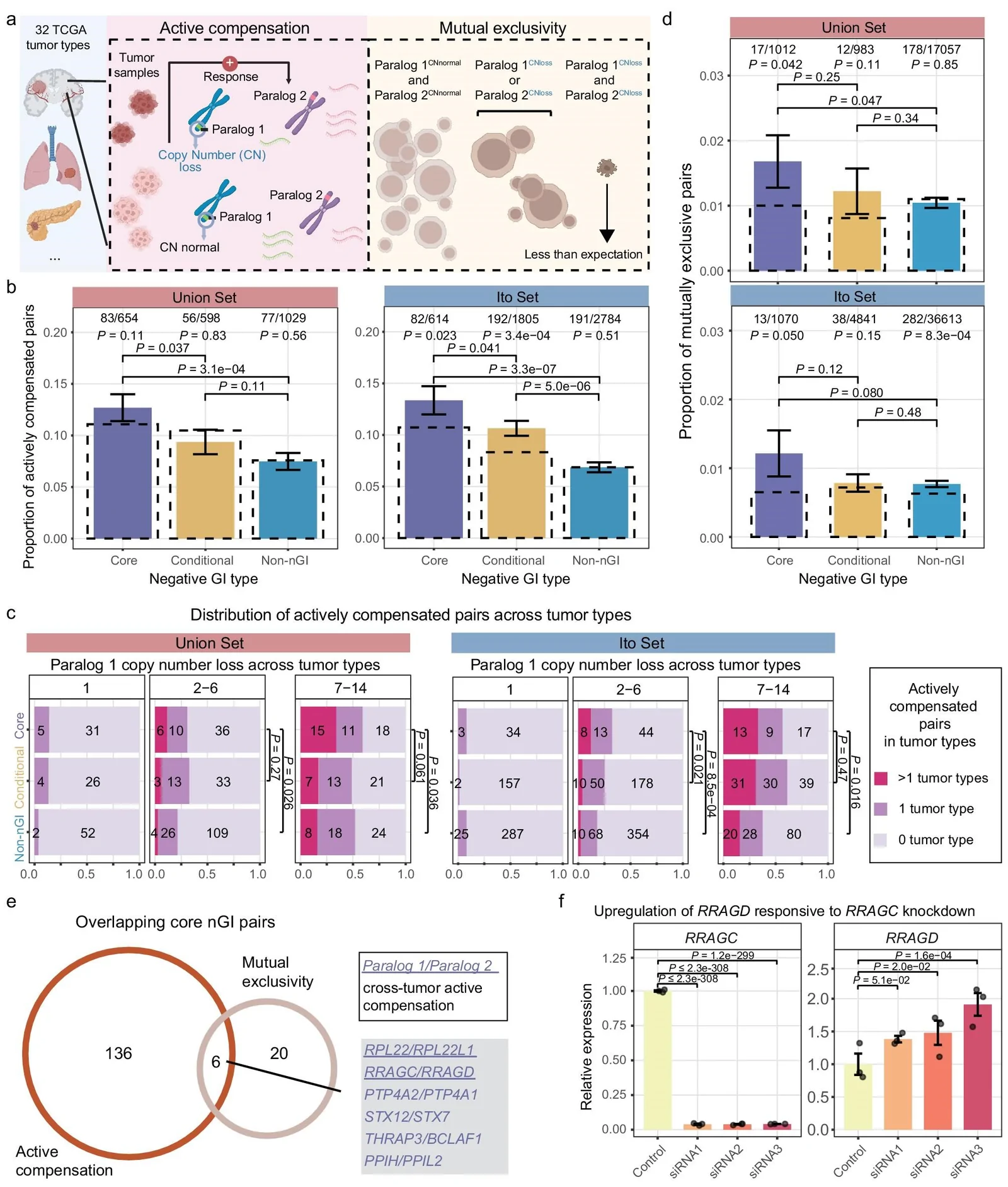
Pan-cancer paralogous compensation analyses. (a) Schematic of the tumor-analytics framework (potential active compensation, mutual exclusivity). Icons are from Freepik (https://www.freepik.com/). (b) Proportion of paralog pairs showing active compensation across nGI groups. The *y*-axis is the fraction of pairs compatible with active compensation in at least one tumor type; the dashed line marks the random-pair baseline (Materials and Methods). One-sided Fisher’s exact tests compare nGI groups; binomial tests compare observed proportions to random. (c) The fraction of cases where paralog 2 is upregulated in >1, exactly 1 or 0 tumor types with paralog 1 copy-number loss in 1, 2–6 or 7–14 tumor types. One-sided Fisher’s exact tests compare nGI groups. (d) Proportion of paralog pairs exhibiting mutual exclusivity across nGI groups. The *y*-axis is the fraction of pairs compatible with mutual exclusivity in at least one cancer type; statistical tests and plotting conventions match panel (b). (e) Venn diagram of six core negative GI pairs identified by both active compensation and mutual exclusivity analyses in the corresponding tumor types. Pairs meeting active compensation criteria in at least two tumor types are underlined. (f) Validation of *RRAGD* upregulation following *RRAGC* knockdown in immortalized astrocytes; *P* values from Wald tests.

Core nGI pairs were found to exhibit significantly higher rates of active compensation compared to conditional and non-nGI groups in both the Union (*P* = 0.037 and 3.1e–04; Fig. 4b, Fig. S4a and Table S7) and Ito (*P* = 0.041 and 3.3e–07) datasets. Randomized analyses seem to be in line with these findings: while the upregulation signal in the Union dataset could be explained by chance (*P* = 0.11), the signal for core nGI pairs in the Ito dataset persisted (*P* = 0.023). More importantly, active compensation was also observed across multiple tumor types, with core nGI pairs showing the highest recurrence of compensatory upregulation in cases where one paralog was lost in at least two cancers (Fig. 4c, Fig. S4a and Table S8). This cross-tumor recurrence reached statistical significance compared to non-nGI groups in both datasets (*P* ≤ 0.036). Multiple well-characterized synthetic lethal pairs, such as *SMARCA2/SMARCA4* [64], *HDAC1/HDAC2* [65] and *EP300/CREBBP* [66], were identified among the cross-tumor pairs, further validating these results.

Core negative GI pairs also showed the highest levels of mutual exclusivity (Fig. 4d and Tables S9 and S10). However, the low frequency of double-loss events in tumors limited the statistical power, with only ∼1% of pairs identified as mutual exclusivity candidates. Despite this, mutual exclusivity was enriched in core nGI pairs, with boundary significance observed between core and non-nGI groups (*P* = 0.047/0.080). Randomized control analyses confirmed that the observed enrichment could not be explained by chance (*P* ≤ 0.050).

The intersection of 142 actively compensated and 26 mutually exclusive candidates revealed six highly reliable buffering paralog pairs (Fig. 4e and Fig. S4b): three previously known compensatory pairs (*RPL22/RPL22L1* [67], *PTP4A2/PTP4A1* [68] and *THRAP3/BCLAF1* [69]) and three newly identified pairs (*RRAGC/RRAGD, STX12/STX7* and *PPIH/PPIL2*). Among these pairs, *RPL22/RPL22L1* and *RRAGC/RRAGD* were associated with cross-tumor active compensation. Notably, the newly discovered *RRAGC/RRAGD* pair encodes key components of the mTORC1 signaling pathway [70], a major regulator of cell growth that is frequently hyperactivated in cancer [71]. In lower-grade glioma (LGG), this pair exhibited both active compensation and mutual exclusivity. Knockdown of *RRAGC* in an immortalized human astrocyte cell line, a model for LGG, led to significant upregulation of *RRAGD*, confirming their compensatory relationship (Fig. 4f). This highlights their potential as a synthetic lethal target in tumors like LGG.

Collectively, although the overall signals of active compensation and mutual exclusivity are moderate, the enrichment of core nGI pairs in these processes suggests that paralogous compensation contributes to cancer cell survival.

### The functional overlap of ancient paralogs appears to align with both pleiotropy and dosage subfunctionalization models

After analyzing organismal paralogous compensation, we asked whether ancient or young duplicates, generated through WGDs or SSDs, contribute to buffering paralogs and whether pleiotropy or dosage subfunctionalization explains the redundancy of these pairs (Fig. 5a).

**Figure 5.**
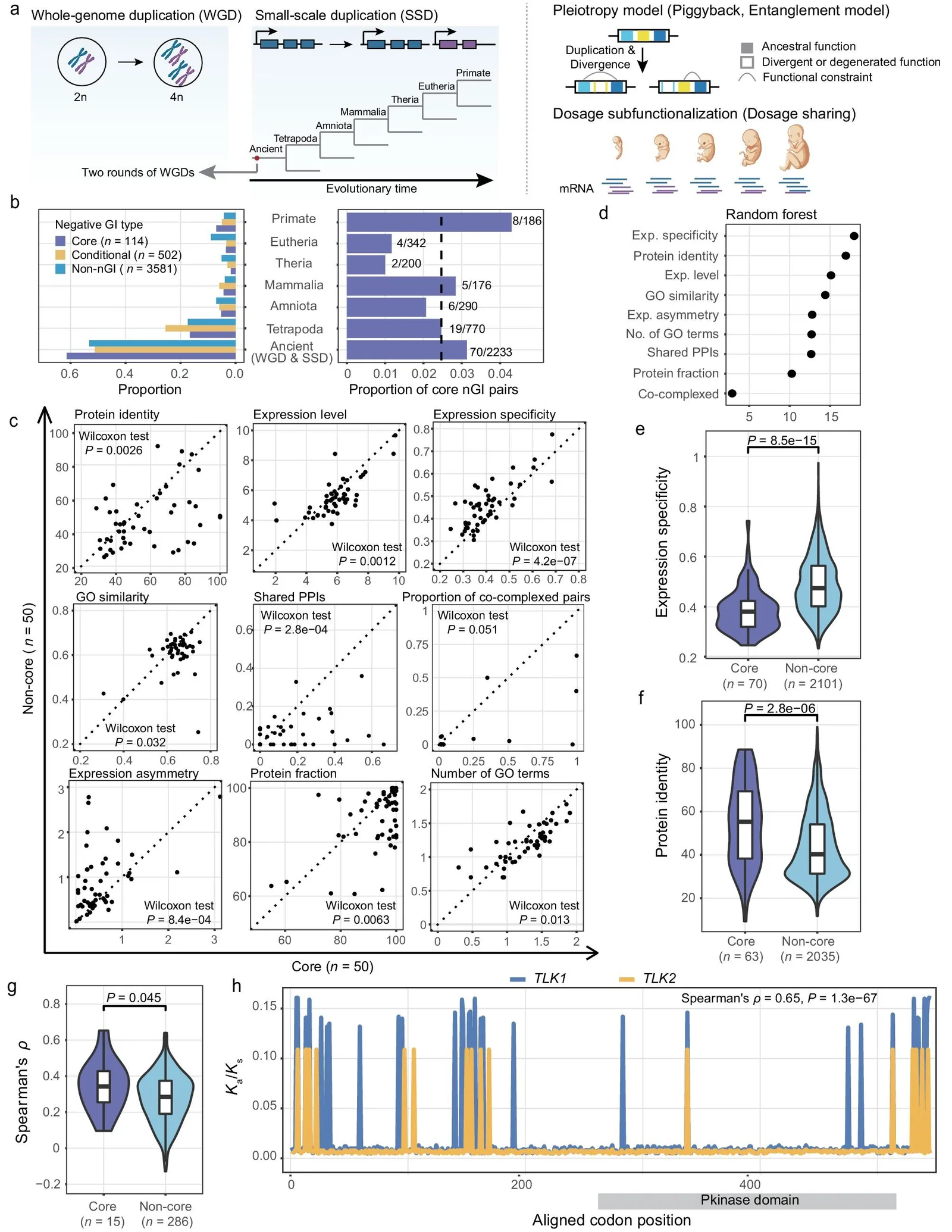
Pleiotropy and dosage subfunctionalization model analyses. (a) Schematic representation of two duplication mechanisms (left) and two evolutionary models for maintaining compensatory capability between paralogs (right). The schematic for the entanglement model is adapted from Kuzmin *et al.* [19]. (b) Distribution of duplication age groups. The left panel depicts the distribution of negative GI paralog pairs across different age groups, categorized into three nGI types. The right panel shows the proportion of core negative GI pairs within each age group. The dashed line in the right panel represents the overall median proportion of core negative GI paralog pairs. (c) Intra-family analysis of sequence and functional features for paralog pairs. Each point in the scatter plot represents a gene family that contains both core and non-core negative GI pairs. Feature values were averaged for core and non-core pairs within each family. *P* values are derived from two-sided Wilcoxon signed-rank tests. (d) Feature importance evaluation for predicting duplicate gene pairs as core negative GI pairs using a random forest model. (e and f) Distribution of expression specificity (e) and protein identity (f) for ancient duplicate gene pairs. For each pair, the mean temporal expression specificity (lower values indicate broader expression; see Materials and Methods) was calculated across organs. The distribution of average specificity for each pair is shown. *P* values were calculated using two-sided Wilcoxon rank-sum tests. (g) Distribution of paralogous correlation in evolutionary rates (*K*_a_/*K*_s_) across different nGI types. The *P* value was derived from a one-sided Wilcoxon rank-sum test. (h) Correlated evolutionary rates of *TLK1* and *TLK2*. The conserved domains are annotated at the bottom. Notably, panels b–g are based on the Ito dataset, while corresponding figures for the Union dataset are presented in Fig. S5i–m.

Using the Ito dataset, due to its large sample size, we performed duplication age analyses and observed two key patterns. First, consistent with the genomic background distribution, more than 60% of core nGI pairs are ancient, originating before the divergence of jawed vertebrates over 445 million years ago [12] (Fig. 5b; Table S11). Second, the proportion of core nGI pairs remains largely consistent across different evolutionary age groups or synonymous divergence bins (${d}_S$, a commonly used proxy of evolutionary time; Fig. S5a), with a median of 2.47% or 2.38%, respectively. This suggests that functional overlap is not just a transient feature of young duplicates, but is also preserved in ancient ones.

The second pattern could be confounded by sgRNA mapping ambiguity for young duplicates, potential under-representation of young duplicates and the bias of core nGI genes toward broadly or highly expressed duplicates. Additional analyses nevertheless support the robustness of the relatively even age-distribution pattern after accounting for these issues. Specifically, we re-examined the pattern using our purpose-built dataset with strict controls for sgRNA mappability, and repeated the analysis after restricting to paralog pairs with coding sequence identity <90%. In both cases, the proportion of core nGI pairs remained broadly comparable across age groups, despite the smaller sample size and consequently increased variance (Fig. S5b and c). We also demonstrated that young duplicates were under-represented relative to the genomic background in the Union dataset but not in the Ito dataset (Fig. S5d), arguing against under-representation of young paralogs as a general explanation for the observed patterns. Finally, we performed logistic regression controlling for cell-line expression breadth, cell-line expression level and sequence identity, and found that adding duplication age did not significantly improve model fit (likelihood-ratio test, *P* = 0.44; Materials and Methods).

The second pattern could also be confounded by the operational definition of core nGI, which may enrich for broadly expressed or functionally essential paralog pairs. We therefore repeated the analysis in the Ito dataset, which provides the large sample across 11 cell lines, while controlling for expression breadth and essentiality (Materials and Methods). Specifically, we first restricted the analysis to paralog pairs co-expressed across all 11 cell lines (the ‘co-expressed subset’) and then further restricted this set to pairs whose mouse orthologs were both annotated as essential (the ‘co-expressed/essential subset’). In both subsets, the proportion of core nGI pairs remained broadly similar across duplication-age groups, consistent with Fig. 5b (Fig. S5e), although variance was greater in the co-expressed/essential subset owing to its smaller sample size.

To test the pleiotropy and dosage subfunctionalization models, we collected sequence, expression and functional features that predict paralogous compensation and reflect pleiotropy/dosage models (Materials and Methods). Protein sequence identity, expression level, expression specificity, GO similarity, shared PPI partners and co-protein-complex membership are six well-established features predicting paralogous compensation [21,24,27–30]. Expression specificity and expression level capture pleiotropy and dosage subfunctionalization respectively, while protein identity bears on both, reflecting pleiotropic/entanglement constraints or slower evolution driven by high expression. We additionally included three less-studied features: expression asymmetry and protein fraction, both of which quantify paralog divergence, and the number of GO terms, which reflects the level of pleiotropy.

A series of statistical analyses demonstrated that expression specificity, followed by protein identity, are the top two features predicting core nGI status. Specifically, because gene families with different molecular functions may vary in their propensity for buffering between duplicate members, we first performed intra-family analyses. These analyses showed that nearly all nine features are significantly associated with core compensatory capability across distinct protein families (Fig. 5c and Table S12). That is, through a family-level analysis comparing core and non-core pairs within the same family, we consistently observed significant contrasts, such as broader expression profiles and higher protein identity for core pairs (Wilcoxon signed-rank test, *P* = 4.2e–07 and 2.6e–03, respectively). Given the well-known interrelationships among these features (Fig. S5f [35]), we utilized a random forest model across all pairs, and discovered that expression specificity and protein identity were the two most powerful predictors of core nGI status (Fig. 5d). Consistently, for the ancient paralog pairs, core nGI pairs were still distinct from their non-compensating counterparts of the same age, showing broader expression (Wilcoxon rank-sum test, *P* = 8.5e–15; Fig. 5e) and higher protein identity (*P* = 2.8e–06; Fig. 5f). Notably, the elevated protein identity of core relative to non-core nGI pairs was also reproduced in both the co-expressed subset and the co-expressed/essential subset (*P* ≤ 0.05; Fig. S5g). Since these two features are central to both the pleiotropy and dosage sharing models, their prominence supports the relevance of these models in explaining paralogous compensation.

To directly test the pleiotropy-based entanglement model [19,25], we analyzed the correlation of evolutionary rates at the codon level for each paralog pair. As predicted, core nGI pairs showed significantly more correlated evolutionary rates than non-core pairs (one-sided Wilcoxon rank-sum test, *P* = 0.045; Fig. 5g and Table S12), suggesting that purifying selection preserves the intertwined functions. A similar pattern was again observed in both the co-expressed and co-expressed/essential subsets (*P* = 0.017 and 0.089, respectively; Fig. S5h). To exemplify, the WGD paralogs *TLK1* and *TLK2*, which function as cell cycle checkpoint kinases [72], exhibit correlated evolutionary rates (Fig. 5h). In both proteins, the N-terminal and the C-terminal regions evolve relatively fast, whereas most regions of the Pkinase domains evolve more slowly and are likely functionally entangled.

These patterns, including the consistent proportion of core nGI pairs across age groups, the predictive power of expression specificity and protein identity and the correlated evolutionary rates of core nGI pairs, were largely replicated in the independent Union dataset (Fig. S5i–m). Together with the Ito-based analyses controlling for potential confounders, such as sgRNA mappability, expression breadth or essentiality, these results support the robustness of these evolutionary patterns.

Overall, paralogous compensation could be a long-term feature preserved in a subset of ancient duplicate genes, which seems to be shaped by the combined forces of pleiotropy and dosage sharing.

### Incomplete SSDs constrain the compensatory capacity of young paralogs, while out-of-X retrogenes exhibit stronger buffering

Notably, despite their presumably high sequence similarity, young paralogs show limited compensatory ability (Fig. 5b and Fig. S5a and c). This observation may result from the incomplete nature of SSD-derived paralogs, which often lack essential coding or regulatory regions (Fig. 6a; [1,73,74]). In contrast, out-of-X retrogenes, i.e. autosomal daughter genes derived from X-linked parental genes via retropositions, may have evolved stronger buffering capacity. These retrogenes are shaped by natural selection to counteract transcriptional silencing of X-linked parental genes during male meiosis, especially pachytene cells (meiotic sex chromosome inactivation [MSCI]) [75,76]. We hypothesized that this selection may have broadened their compensatory functionality, extending beyond the germline to somatic tissues.

**Figure 6.**
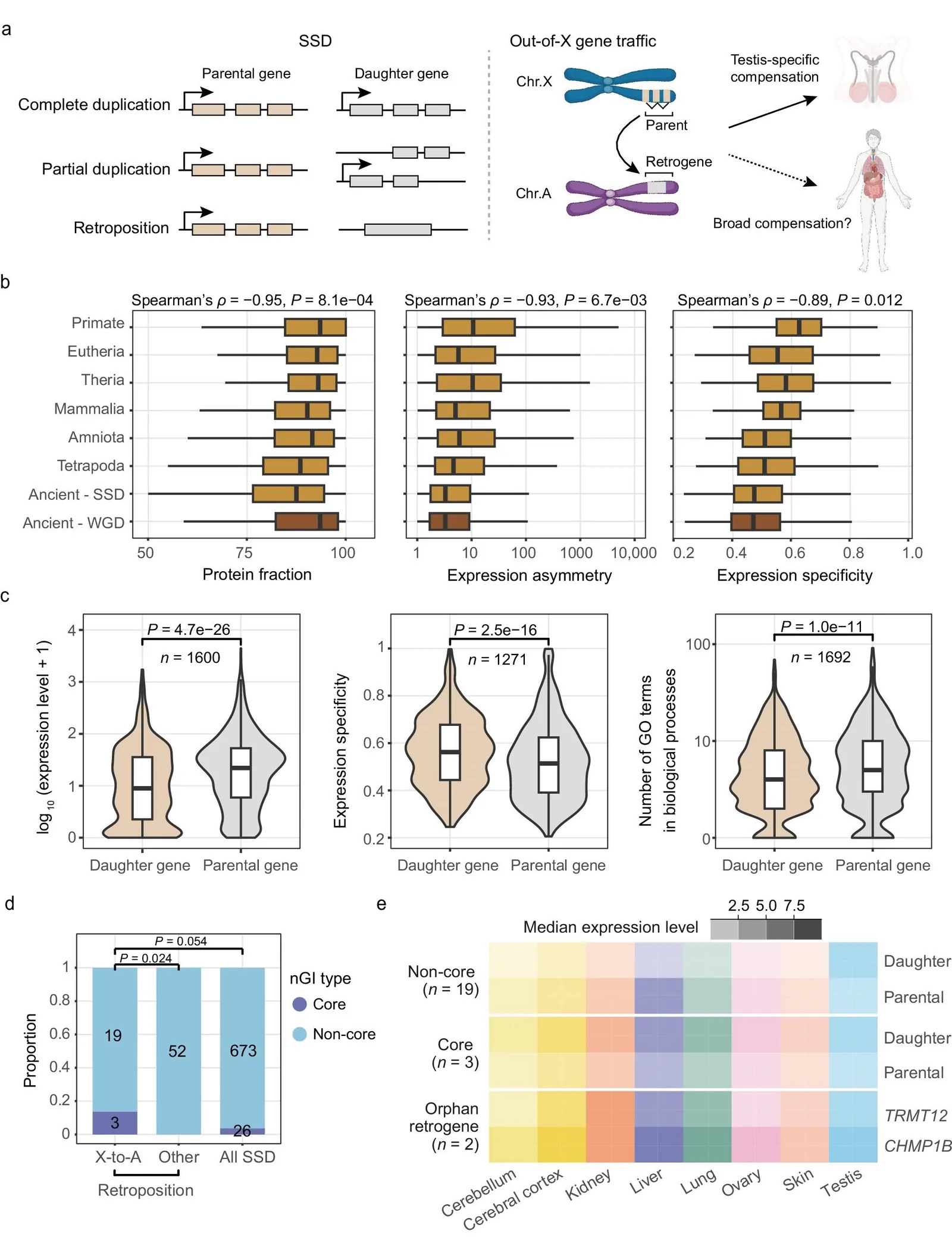
The SSD pair and out-of-X retrogene analyses. (a) Schematic representation of SSD events (left) and out-of-X retroposition (right). SSD events are classified into complete duplication, partial duplication (5ʹ or 3ʹ) and retroposition. Retrogenes may compensate for their X-linked parental genes in both testis and other tissues. (b) Distribution of protein alignment fraction, expression asymmetry and expression specificity across SSD age groups. For each SSD pair, protein alignment fraction and expression specificity (τ) were averaged across the two paralogs, whereas expression asymmetry was calculated at the pair level. Correlations were calculated between SSD age group and the median value of each feature across age groups. (c) Distribution of expression and functional features of daughter and parental genes. *P* values are derived from two-sided Wilcoxon signed-rank tests. (d) Proportion of core negative GI pairs among paralog pairs originating from retroposition only and all SSD mechanisms. Retroposed pairs are further categorized based on the chromosomal locations of daughter and parental genes. The *P* values are derived from two-sided Fisher’s exact tests. (e) Median expression levels of X-derived retrogenes (including two orphan retrogenes) across various organs. For non-orphan retrogenes, the expression levels of their X-linked parental genes are also included. Note: SSD panels in b and c are based on the Ito dataset, while corresponding figures for the Union dataset are presented in Fig. S6a and b; Panels (d) to (e) are based on the merged dataset of the Union and Ito datasets.

Using the Ito dataset with a larger sample size, we tested three predictions of the incomplete duplication hypothesis (Materials and Methods): incomplete protein sequence alignment, high expression asymmetry caused by low expression of daughter copies and high expression specificity caused by narrow expression breadth of daughter copies. The youngest primate-specific paralogs had a median alignment of 93.5%, with only 25.8% (48/186) showing 100% alignment, with the loss of protein terminals indicating widespread absence of 5ʹ or 3ʹ regulatory sequences (Fig. 6b). These pairs also exhibited high expression asymmetry and specificity, aligning with the low expression levels and tissue specificity typical of young genes [21,77,78]. Consistently, compared to their parental genes, daughter copies had significantly lower expression levels, narrower expression breadth and reduced pleiotropy, as measured by GO terms (Fig. 6c).

Interestingly, as duplicate age increased, the alignable fraction declined (Spearman’s *ρ* = −0.95, *P* = 8.1e–04; Fig. 6b), likely due to accumulating sequence divergence. Concurrently, expression asymmetry and specificity also decreased (*ρ* ∼ −0.90, *P* ≤ 0.012), eventually converging to levels observed in WGD pairs. These findings suggest that SSD-derived duplicates are initially constrained by their incomplete nature, limiting their buffering capacity. Over time, selection likely optimizes their regulatory features and enhances their expression, increasing compensatory potential.

Notably, the major patterns, such as the high expression specificity of daughter duplicates and its age-dependent decrease, were largely replicated in the smaller Union dataset (Fig. S6a and b), reinforcing the prevalence of SSD incompleteness and the gradual expression evolution.

In this gradual evolutionary trajectory, out-of-X retrogenes represent an exceptional class, exhibiting three patterns consistent with MSCI. First, analysis of the merged Union and Ito datasets revealed that X-derived retrogenes are enriched in core nGI pairs compared to other retrogenes (e.g. 13.6% vs. 0%, *P* = 0.024; Fig. 6d; Materials and Methods), or relative to all SSD-derived pairs in the merged datasets (3.72%, *P* = 0.054; Fig. 6d). Second, while non-compensating X-derived retrogenes were, as expected, often mainly expressed in the testis, with upregulation timed to the pachytene stage of meiosis, core nGI retrogenes had evolved broad expression patterns across somatic and germline tissues, often at levels comparable to or even exceeding their parental genes (Fig. 6e and Fig. S6d). Third, all core nGI retrogenes originated in early eutherian evolution, coinciding with the reported emergence of a wave of compensatory retrogenes during the rapid establishment of MSCI after the origin of therian sex chromosomes [76] (Fig. S6c and Table S12). Therefore, despite the loss of original promoters during retroposition, these core retrogenes appear to have gradually acquired broad expression, especially considering their birth in the eutherian ancestor 100 million years ago. As an extreme outcome, X-derived retrogenes can replace their parental copies, leading to parental loss: of the three known orphan retrogenes that have lost their parental genes in the mammalian lineage [79], two arose from the X chromosome (Fig. S6e). Similar to retrogenes in core nGI pairs, these two genes are expressed in pachytene spermatocytes as well as in various somatic tissues (Fig. 6e and Fig. S6d).

In summary, the limited compensatory potential of young paralogs stems from the incomplete nature of SSD mechanisms. However, over evolutionary time, derived duplicates, particularly X-derived retrogenes, can acquire enhanced compensatory capacity through an increase of expression breadth.

## DISCUSSION

By assembling two complementary cellular datasets of paralog compensation (Union and Ito) and performing inference analyses, we detected moderate *in vivo* compensation (Fig. 7a) and highlighted the roles of selection and mutation in shaping these mechanisms (Fig. 7b).

**Figure 7.**
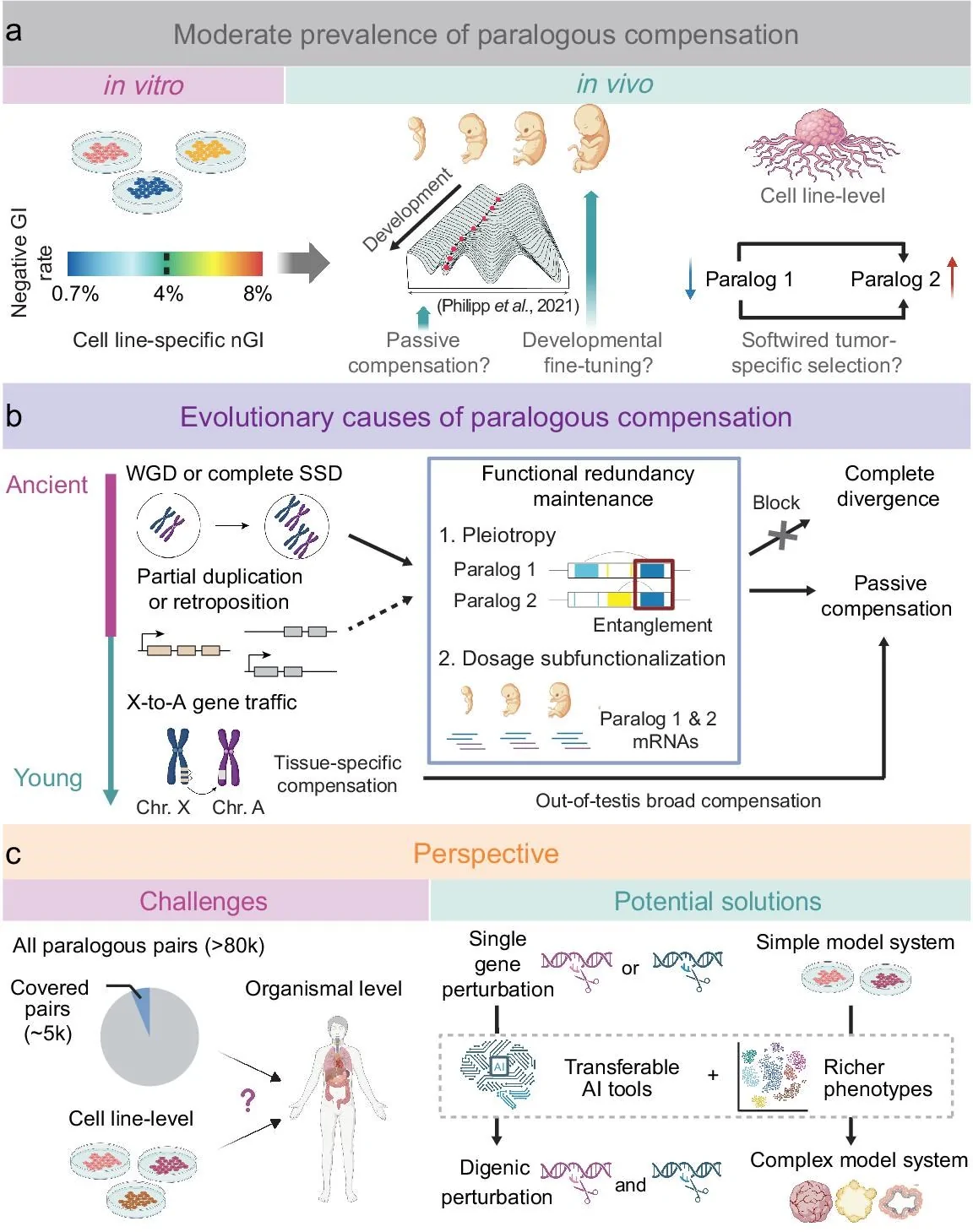
An overview and perspective on paralogous compensation in humans.

On one hand, our analyses, integrating *in vitro* cell line screens and *in vivo* computational inferences, converge on a finding: paralogous compensation is not prevalent in humans (Fig. 7a). Consistent with prior studies, only a small fraction of paralog pairs (∼4% median) exhibit nGIs across cancer and normal cell types (Fig. 2b), far fewer than in yeast (∼30% [24,27–30]) and worms (∼11% [31,32]). This contrast likely reflects both methodological and biological differences among systems. Perturbation approaches differ across species, synthetic genetic arrays in yeast, RNA interference (RNAi) in worms and CRISPR-based screens in humans, as do the functional contexts, spanning cells, whole organisms and cell lines, respectively. Biological differences may also contribute, including higher-order compensation arising from two rounds of WGD in humans (Fig. 5a [12]), the possibility that human duplicates more often create fragility through heteromeric interactions than confer robustness [80], and reduced compensatory capacity associated with cell-type specialization in worms and humans. Consistent with the latter, putatively expression-specialized paralog pairs in the Ito dataset showed markedly lower nGI proportions than putatively redundant pairs (1.5%–2.1% vs. 5.0%; *P* = 1.6e–26; Fig. S7a and b; Materials and Methods).

Besides human cell lines, compensation is also moderate in two *in vivo* contexts: development and cancer. For development, core nGI pairs show strong co-expression and are depleted from disease-gene lists, consistent with passive buffering that contributes to canalization and mitigates mutational effects [48,81,82]. This signal is stronger for core than for conditional or non-nGI groups but only marginally significant (*P* ∼ 0.05; Fig. 3b and c), suggesting buffering is not widespread. Co-expression may also reflect fine-tuned overlap with distinct roles [83,84]; for example, *ROCK1* and *ROCK2* knockouts both reduce hippocampal spine density, but only *ROCK2* alters spine morphology [60]. In cancer, active compensation (∼12%) aligns with prior tumor proteomics studies [85], but a substantial proportion appears attributable to random chance (Fig. 4b). The remaining *bona fide* compensations likely arise through two mechanisms: rare, hardwired regulatory circuits that directly respond to paralog loss (e.g. *RPL22/RPL22L1* and *RRAGC/RRAGD*) and softwired responses driven by tumor selection. The aforementioned *RPL22/RPL22L1* and *RRAGC/RRAGD* fit the first scenario: *RPL22* directly represses *RPL22L1* by inducing messenger RNA (mRNA) degradation (Fig. S7c [67]), while *RRAGC/RRAGD* interaction is validated in normal cells, i.e. the immortalized astrocytes (Fig. 4f). The second mechanism is supported by the overrepresentation of oncogenes in upregulated paralogs in the Ito dataset (Fig. S7d and Table S8), consistent with its tumor enzyme focus [42]. Notably, prioritized core nGI pairs, such as *ROCK1/ROCK2* and *RRAGC/RRAGD* (Figs 3d and 4e), implicated in development and tumor biology, warrant further exploration in the contexts of canalization [81,86,87] and synthetic lethality-based cancer therapy [38–45].

On the other hand, paralogous compensation is shaped by multiple selective forces and duplication incompleteness (Fig. 7b). Analysis of evolutionary features, including duplication fraction, along with previously identified predictors of compensation, highlights expression specificity and protein identity as the two most critical factors (Fig. 5d and Fig. S5j). These findings align with both pleiotropy and dosage subfunctionalization models. Correlated evolutionary rates further support a refined pleiotropy model, i.e. the entanglement model (initially proposed for yeast), where negative selection on unshared functions constrains divergence of shared functions, preserving compensatory potential (Fig. 5a and g; [19,25]). The dosage subfunctionalization model suggests that under positive selection or genetic drift, duplicates may initially evolve to match the ancestral gene’s total dosage. Co-expression is then maintained over long-term evolution (Fig. 3b [21,88]). Together, pleiotropy and dosage constraints may limit the complete functional divergence of ancient paralogs, thereby preserving passive compensation. Other biological and evolutionary factors may also contribute; for example, duplicated essential genes more frequently showed compensation (Fig. S5e).

Young duplicates, however, do not exhibit the expected high compensation potential compared to other age groups (Fig. 5b and Fig. S5a and c), as many lose coding or regulatory regions upon duplication (Fig. 6b). This incompleteness, consistent with prior studies on segmental duplications and retropositions, limits compensatory capacity. As an exception, out-of-X retrogenes display high somatic compensatory capability (Fig. 6d and e). Expression profiling and functional assays reveal that these genes buffer their X-linked parental counterparts in the male germline, likely driven by positive selection [76,89]. As retrogenes typically follow an out-of-testis evolutionary trajectory (initially expressed in testis due to open chromatin and later expanding to other tissues), they evolve broad compensatory abilities, even replacing parental copies (Fig. 6e). Additionally, the age-dependent increase in expression level and breadth of duplicate genes from SSDs (Fig. 6b [77,88]) indicates that such duplicates gradually overcome mutational constraints and gain compensatory power. In contrast, WGD paralogs are born with full compensation potential.

Notably, despite our extensive efforts, the current map of paralogous interactions captures only ∼6% of the 80 000 paralog pairs in the human genome, and extrapolations from cell lines to whole organisms are associated with some uncertainty (Fig. 7c). Artificial intelligence (AI)-driven virtual cell models hold promise for predicting higher-order interactions from single-gene perturbation data [90], while combining CRISPR screening with organoid technologies enables the study of GIs in more physiologically relevant, tissue-like contexts [91]. Integrating these rapidly evolving computational and experimental approaches could yield a more comprehensive blueprint of the human paralog interaction network, providing deeper insights into the evolutionary forces shaping the genetic robustness.

In conclusion, the evolutionary remnants of residual functional overlap in ancient, entangled paralogs, combined with the limited redundancy of incomplete young duplicates, result in a moderate level of paralogous compensation in humans.

## MATERIALS AND METHODS

Please refer to the Supplementary Methods section of the Supplementary Data for detailed methodological descriptions.

### Generation of an association dataset comprising 386 pairs

An association dataset of predicted nGIs was compiled from two published single gene essentiality screens: De Kegel and Ryan (374 paralog pairs) [35] and Dede *et al.* (66 pairs) [40]. After merging and removing redundant entries, 386 unique paralog pairs were retained and served as an independent benchmark to assess the quality of experimental screens and to define core negative GI pairs.

### Feature enrichment analyses across CRISPR screening datasets

Paralog features were obtained from the CORUM 3.0 database (protein complexes) [92], Ensembl BioMart v96 (protein sequence identity) [93], PantherDB v17.0 (gene families) [94] and BioPlex v3.0 (PPI) [95]. Functional similarity was calculated using semantic similarity of GO terms with GOSemSim [96].

### Construction and characterization of the Union and Ito datasets

The Union dataset comprised 2168 non-redundant paralog pairs from Dede *et al.* [40], Parrish *et al.* [39], Thompson *et al.* [41] and this study; the Ito dataset comprised 4859 pairs from Ito *et al.* [42], focusing on enzymatic paralogs. To classify the robustness of the observed GIs, pairs were classified as core, conditional or non-nGI. Core nGIs were defined as occurring in >50% (40%) of assayed cell lines in Union (Ito), or as present in the association dataset. Other pairs with nGIs in fewer cell lines were classified as conditional nGIs, and those without nGIs as non-nGIs.

### Developmental co-expression and disease-gene depletion analyses

Developmental RNA sequencing (RNA-seq) data from seven human organs were obtained from Cardoso-Moreira *et al.* [54] and quantified with Kallisto v0.46.0 [97]. Expression profiles were rank-normalized [98] and Pearson correlations between paralogs were calculated separately for prenatal and postnatal stages. Significant co-expression was defined as reciprocal top 5% correlation.

Disease-associated genes were compiled from ClinGen [55], DDG2P v4.6 [56] and HGMD developmental-disorder datasets [58] compiled by Cardoso-Moreira *et al.* [57]. Disease-gene depletion among co-expressed versus non-co-expressed paralogs was assessed for each nGI category using odds ratios and one-sided Fisher’s exact tests.

### Active compensation and mutual exclusivity analysis in tumor samples

Pan-cancer Copy Number Variation (CNV) and Z-score-normalized expression data were obtained from TCGA via cBioPortal [99]. Active compensation was defined as significant upregulation of one paralog in samples with CN loss, as defined by Genomic Identification of Significant Targets in Cancer (GISTIC) scores of −1 or −2 of its partner, restricted to genes with CN loss in >30% of samples or among the 500 most frequently deleted genes. One-sided *t*-tests were used to assess paralog 1 loss and paralog 2 upregulation (false discovery rate [FDR] < 0.001 and <0.05, respectively). Compensation across tumor types was classified as cross-tumor, tumor-specific or absent and compared using one-sided Fisher’s exact tests.

Mutual exclusivity was tested using 2 × 2 loss-event tables and one-sided Fisher’s exact tests (FDR < 0.1), restricted to pairs with high CN-loss rates and at least one double-loss event.

To validate predicted active compensation between *RRAGC* and *RRAGD, RRAGC* was knocked down using three siRNAs or a negative control in immortalized human astrocytes to assess the potential upregulation of *RRAGD*.

### Entanglement analysis

To test the entanglement model of paralog retention, we adapted the methodology of Kuzmin *et al.* [19] to vertebrates. Orthologs from eight representative vertebrates were obtained from Ensembl. Multiple sequence alignments of the proteins and codons were generated using MAFFT v7.505 [100] and PAL2NAL v14 [101], separately. Site-specific ω (*K*_a_/*K*_s_) values for each paralog were estimated with the M7 model of codeml from PAML v4.9j [102] and Spearman’s correlation between paralogs’ codon-level ω values was used to quantify co-evolutionary constraint or entanglement.

### Expression and sequence feature analyses

Duplication age and duplication mechanism (WGD/SSD) were obtained from GenTree [103] and OHNOLOGS v2 [104]. Pairs annotated by OHNOLOGS v2 and dated to the vertebrate common ancestor in GenTree were classified as WGD-derived; others were classified as SSD-derived, with younger copy age used as the duplication age.

Nine features were collected to explore the predictors of compensation. Protein identity, expression level, asymmetry, temporal specificity (τ index [105]), GO features, PPI sharing and protein complex membership were obtained from public databases [93], developmental transcriptomes [54] or resources described in the section Feature enrichment analyses across CRISPR screening datasets. Protein alignment fractions were calculated from reciprocal BLASTP v2.2.26 alignments [106].

Core and non-core pairs were compared using two-sided Wilcoxon tests, with family-level averaging for the 50 Ito gene families containing both classes (Table S12). Within each family, we averaged features for core and for non-core pairs, then compared these averages with a paired two-sided Wilcoxon signed-rank test. A random forest model (randomForest v4.7-1.1 [107]) assessed feature importance in predicting core nGI status.

Logistic regression in the Ito dataset tested whether duplication age independently predicted core nGI status among pairs with CDS identity <90%, using expression breadth, expression level and protein sequence identity as covariates.

### Retrogene analyses

The Ito and Union datasets were merged to classify retrogene-associated pairs. Retrogenes were identified using RetrogeneDB [108] and GenTree [103], yielding 22 X-derived pairs (Table S12). Expression was assessed using HPA v22.0 bulk RNA-seq [109] and human spermatogenesis single-cell RNA-seq [110]. Orphan retrogenes were obtained from Ciomborowska *et al.* [79].

### Statistical analysis

Statistical analyses were performed using R. Depending on context, significance was assessed using tests such as the Binomial test, Fisher’s exact test or Wilcoxon rank-sum test. Sample sizes and exact *P* values were reported.

## Supplementary Material

nwag565_Supplemental_Files

## Data Availability

The raw sequencing data generated in this study have been submitted to the NCBI BioProject database (https://www.ncbi.nlm.nih.gov/bioproject/) and NGDC GSA database (https://ngdc.cncb.ac.cn/gsa/) under accession numbers PRJNA782415 and PRJCA007405, respectively. The code for all figures, datasets and the variance-weighted calculation of negative GIs were submitted to Zenodo (doi: 10.5281/zenodo.16938996).
