# Supplementary material for: Moderate prevalence and evolutionary causes of paralogous compensation in humans
Source: Natl Sci Rev. 2026 Sep 3;13(19):nwag565. doi: 10.1093/nsr/nwag565 (PMC13715443; doi:10.1093/nsr/nwag565)
Supplement: Supplementary file 1 [file nwag565_supplemental_files.zip › Supplementary Figures/Supplementary Figure1.pdf]

a

| | Editing efficiency | Between-replicate correlation | Rediscovery of positive controls | Correlation with swapped sgRNA | Validation rate | Off-target control criterion |
|---|---|---|---|---|---|---|
| Dede *et al.* 2020 | 80% | unreported | - | unreported | - | DeWeirdt library + empirical rules |
| **GP *et al.* 2020** | 10-43% | - | - | unreported | 4/6; - | CHyMErA + empirical rules |
| Parrish *et al.* 2021 | - | 0.82-0.92 | - | - | 4/4; 2/2 | Brunello library |
| **Thompson *et al.* 2021** | 54% | - | - | - | 5/7-7/7; 1/1 | WGE rules |
| Ito *et al.* 2021 | - | unreported | unreported | 0.47 | unreported | unreported |
| **Anvar *et al.* 2024** | - | 0.78-0.92 | 0.75 | unreported | 2/2; 2/2 | CRISPick |
| **This work** | 67-86% | 0.80-0.87 | 0.43 | 0.47-0.56 | 7/10; 5/7 | Position-aware empirical rules |

b

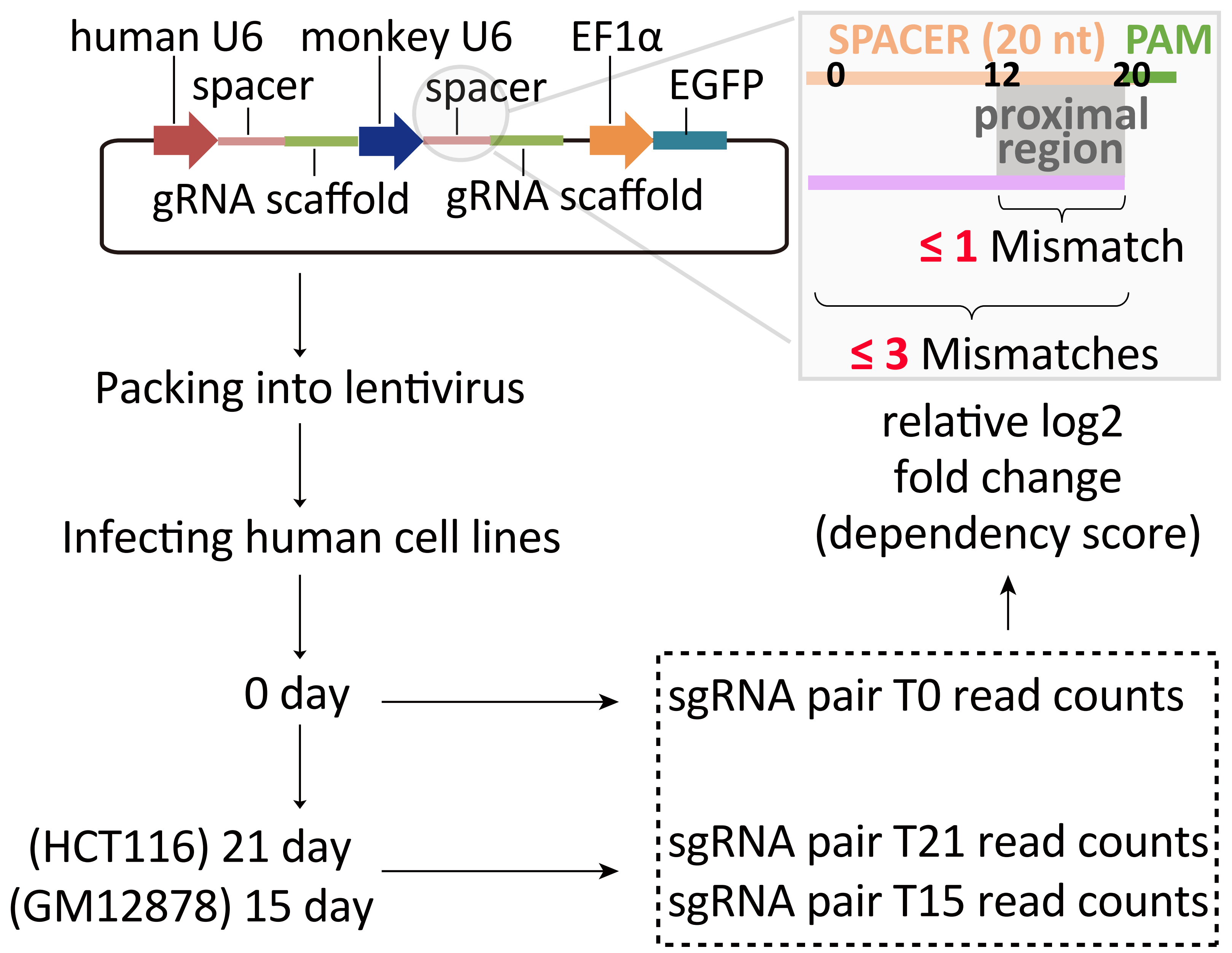


c

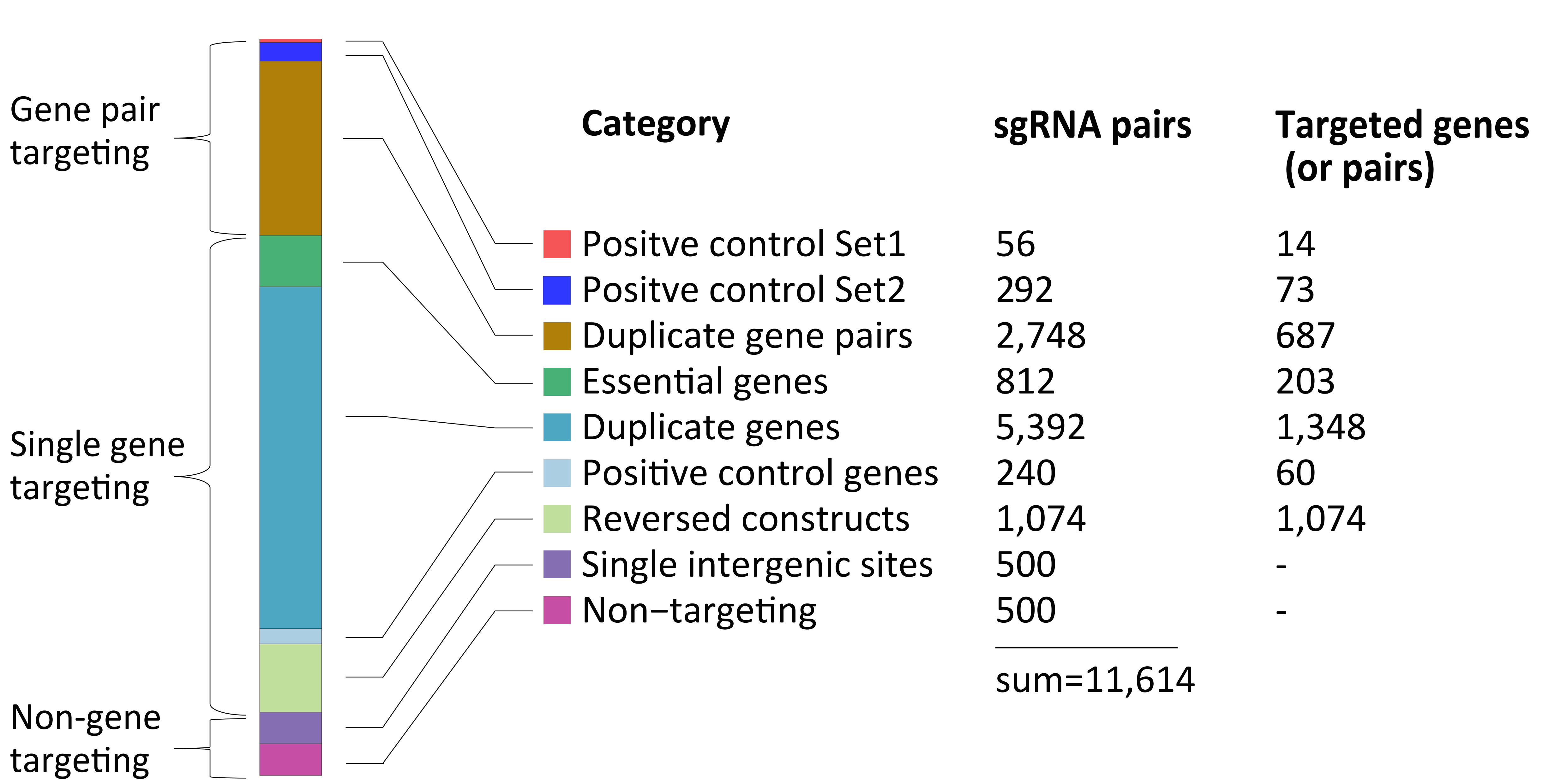


d

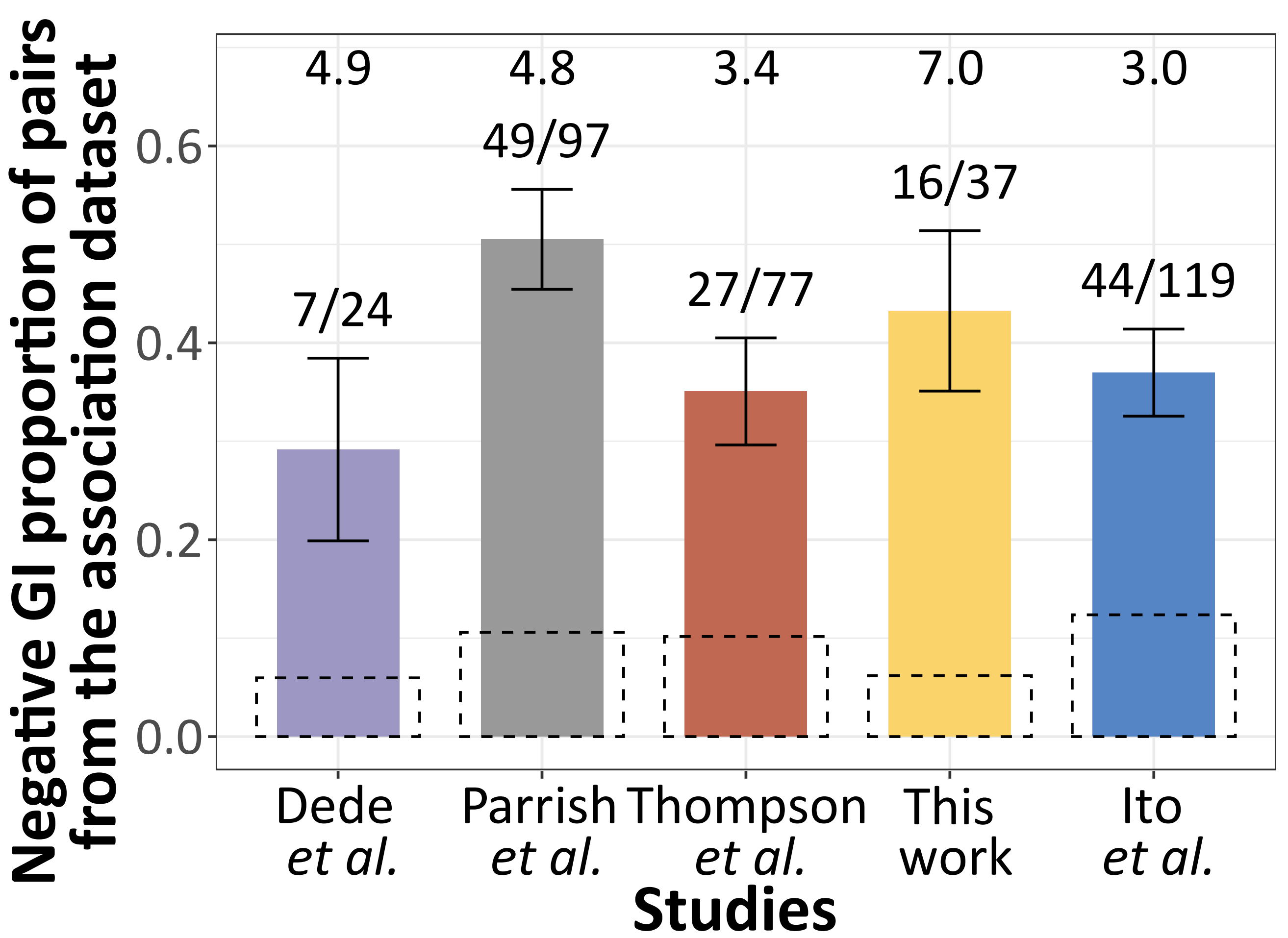


e

FDR values for feature enrichment tests between negative GI (nGI) and non-nGI pairs in different studies.

| | Association set | Co-complexed | Protein Identity | Shared PPIs | GO similarity |
|---|---|---|---|---|---|
| **Dede *et al.*** | 3.8e-04 | 0.13 | 1.5e-05 | 6.3e-03 | 0.13 |
| **Parrish *et al.*** | 5.5e-22 | 2.3e-07 | 7.5e-06 | 4.6e-05 | 1.7e-05 |
| **Thompson *et al.*** | 5.0e-09 | 6.4e-04 | 5.8e-05 | 3.5e-04 | 4.8e-05 |
| **This work** | 2.8e-10 | 1.6e-04 | 4.1e-03 | 2.3e-03 | 7.5e-04 |
| **Ito *et al.*** | 1.6e-11 | 1.3e-08 | 0.026 | 4.4e-03 | 0.069 |
