# Supplementary material for: Moderate prevalence and evolutionary causes of paralogous compensation in humans
Source: Natl Sci Rev. 2026 Sep 3;13(19):nwag565. doi: 10.1093/nsr/nwag565 (PMC13715443; doi:10.1093/nsr/nwag565)

a

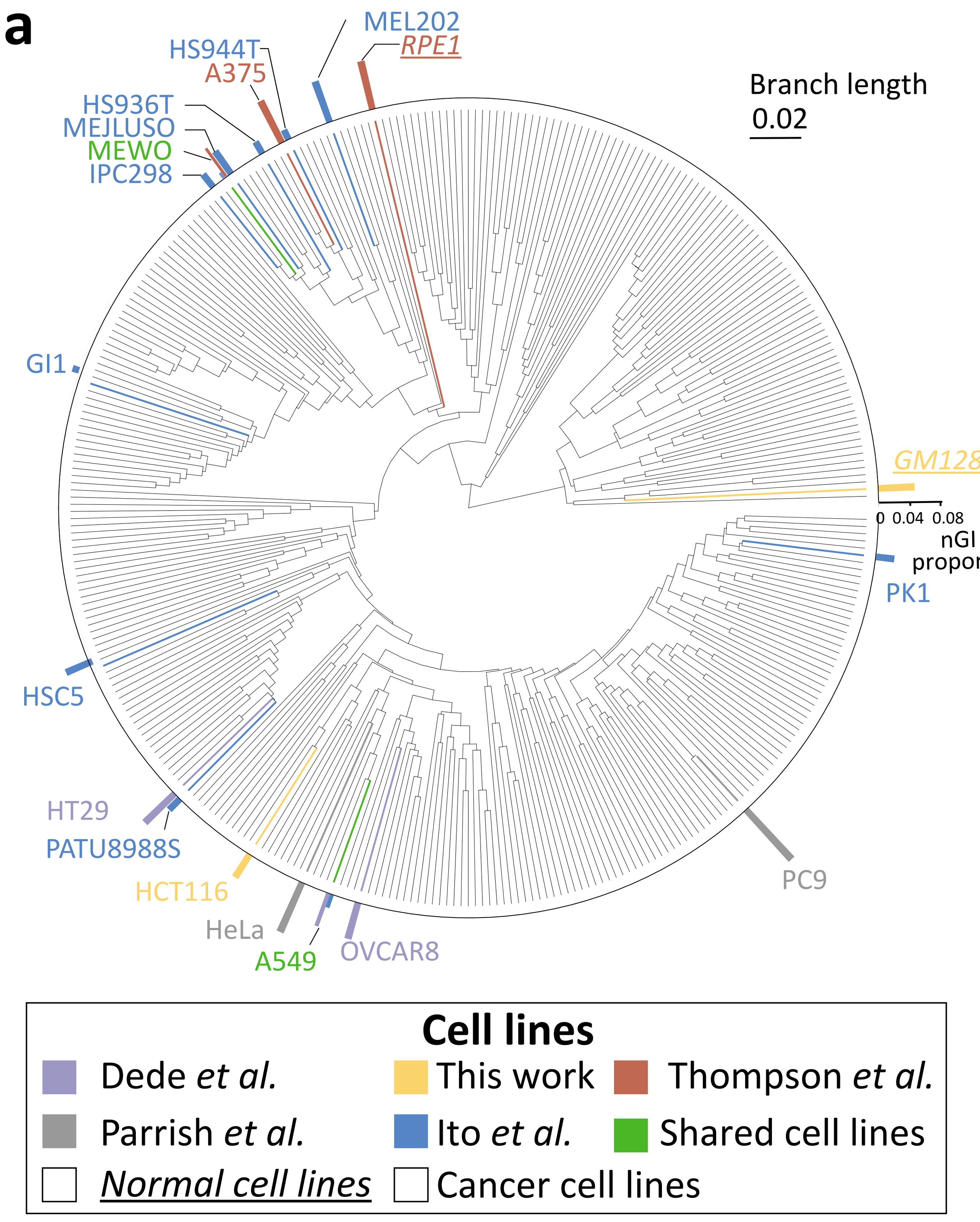

b

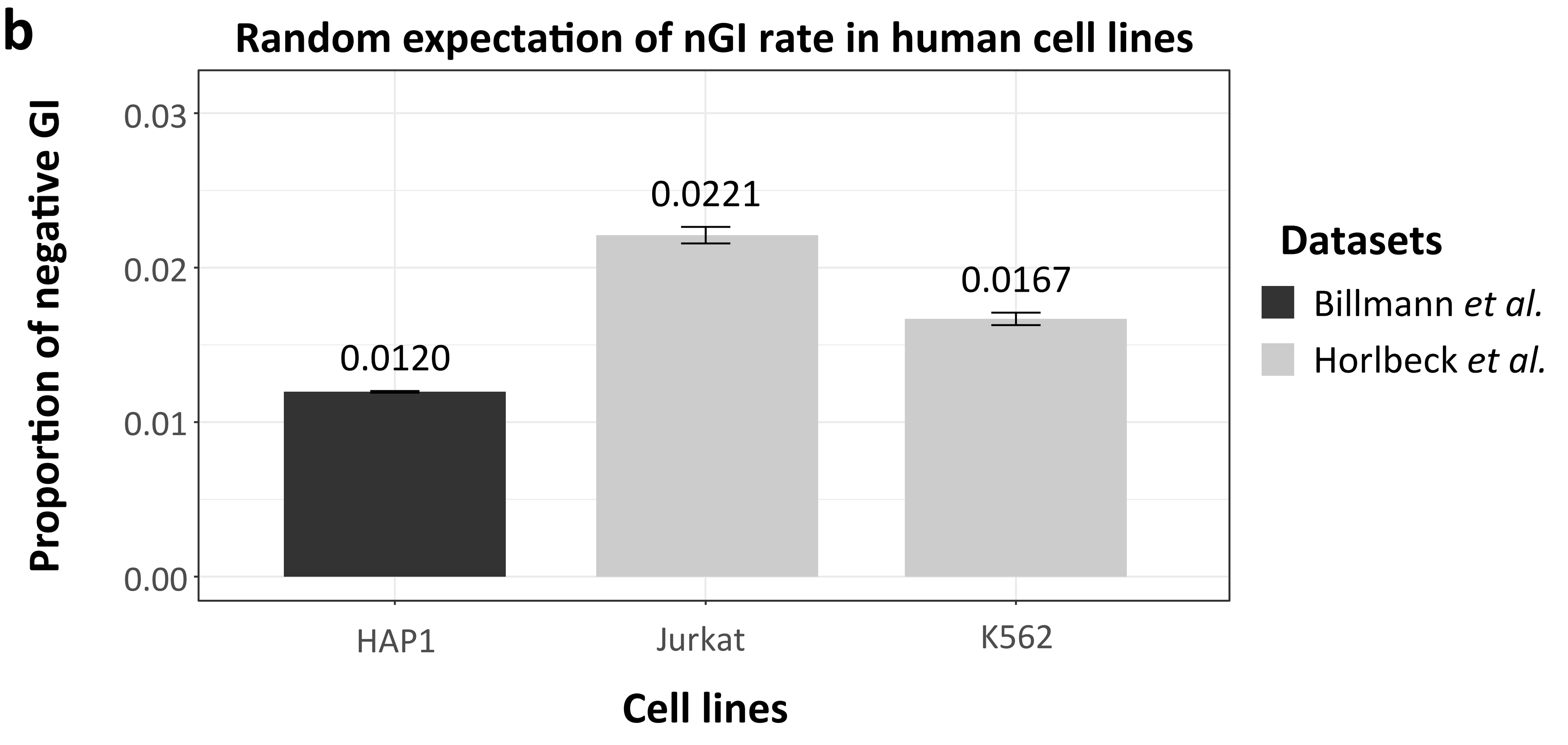

c

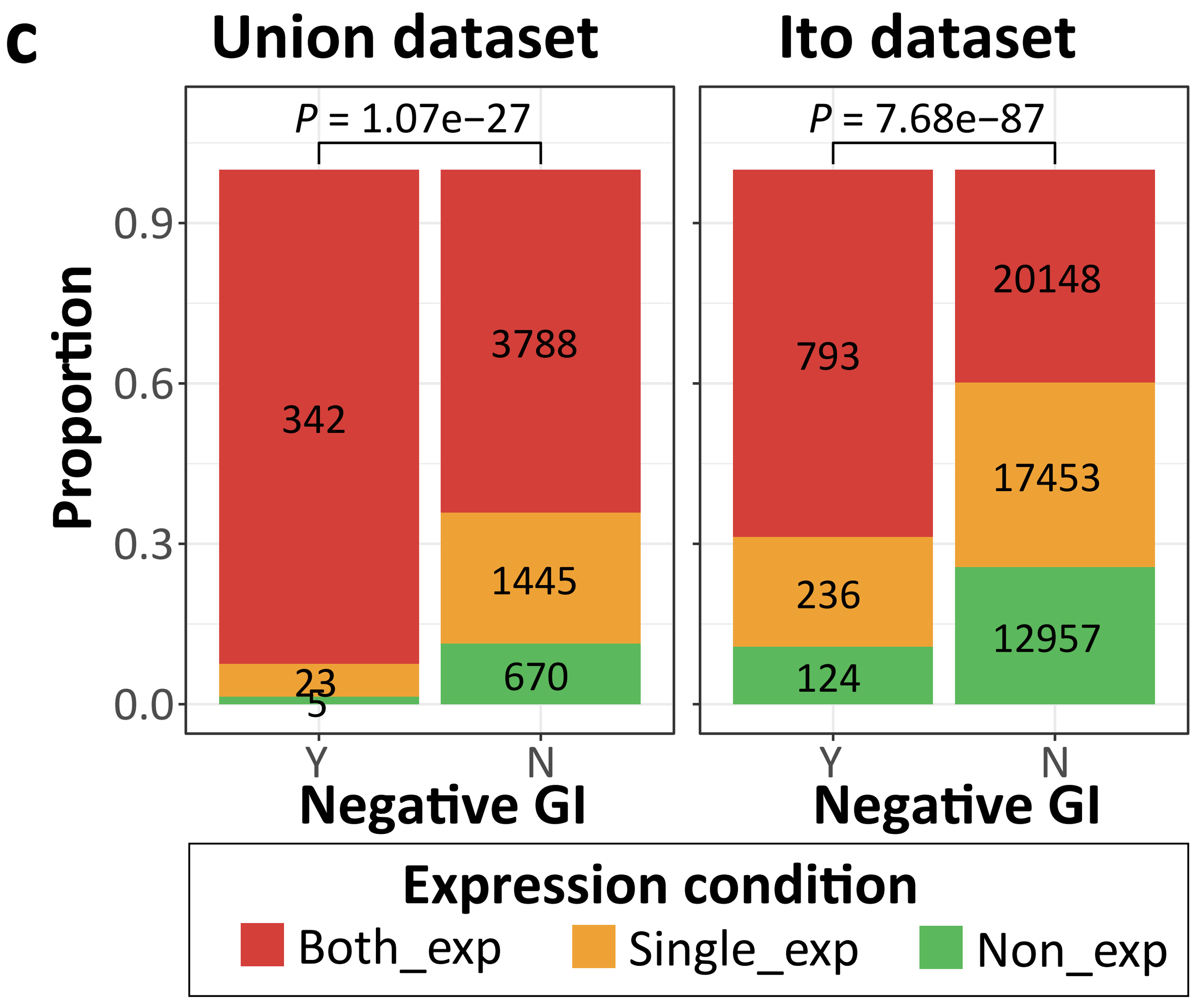

d

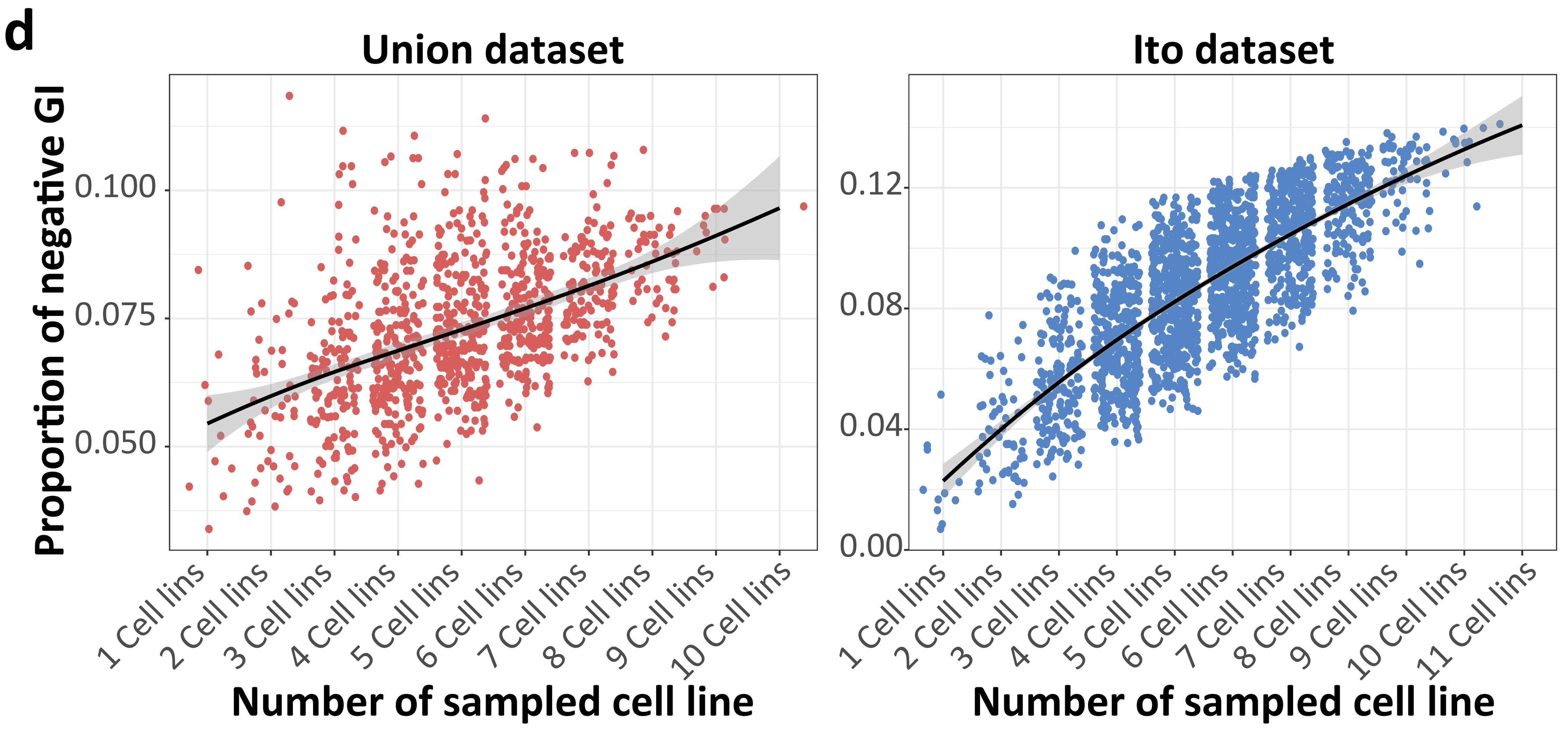

e

Cell line regrouping in Ito dataset

Cell lines	Origin	Re-grouping
MeWo	Skin melanoma	Skin melanoma
MELJUSO	Skin melanoma	
IPC298	Skin melanoma	
HS944T	Skin melanoma	
HS936T	Skin melanoma	
HSC5	Skin squamous cell carcinoma	HSC5
MEL202	Ocular melanoma	MEL202
A549	Non-small cell lung cancer	A549
PATU8988S	Pancreatic adenocarcinoma	PATU8988S
PK1	Pancreatic adenocarcinoma	PK1
GI1	Brain Gliosarcoma	GI1

f

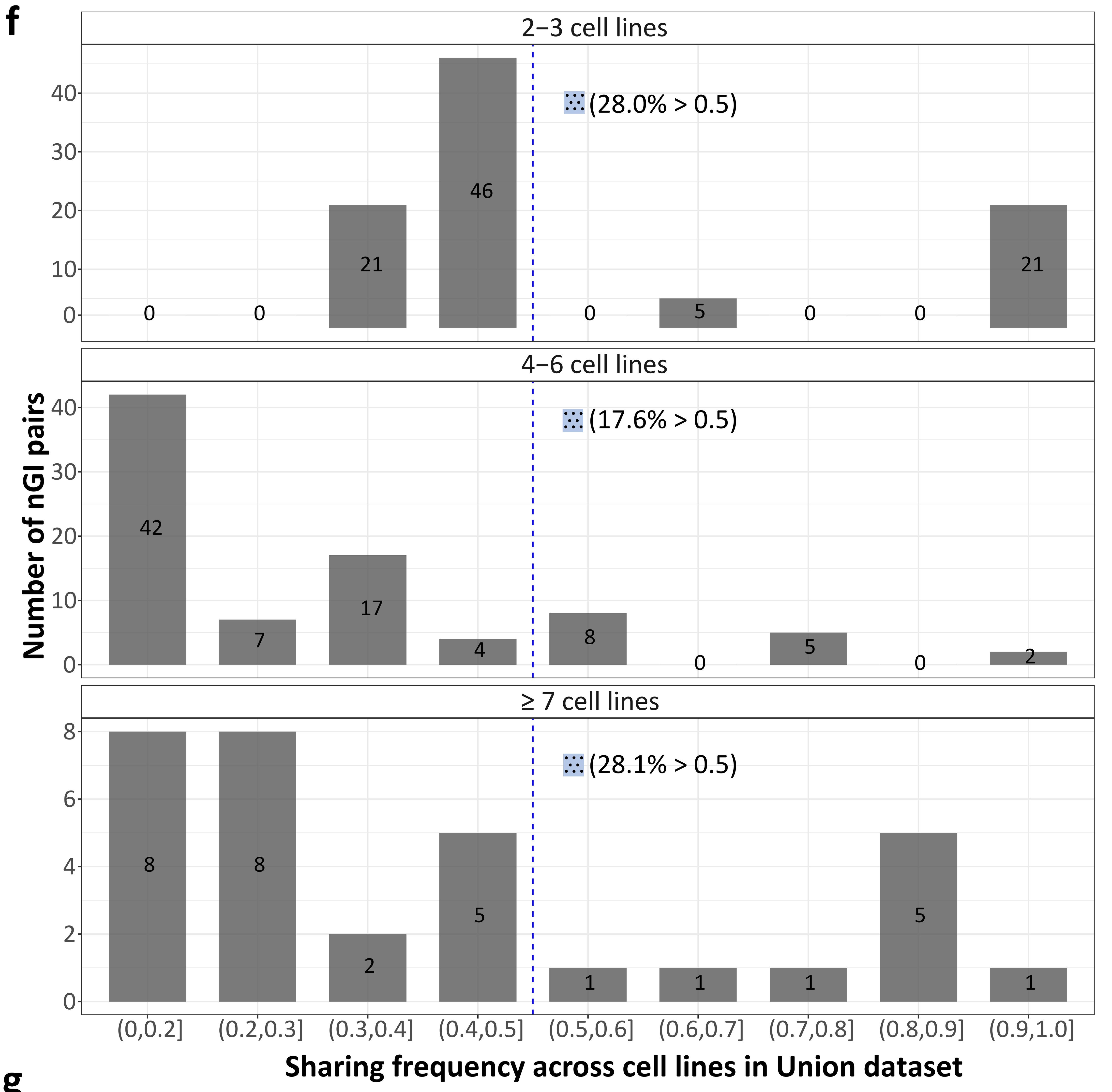

g

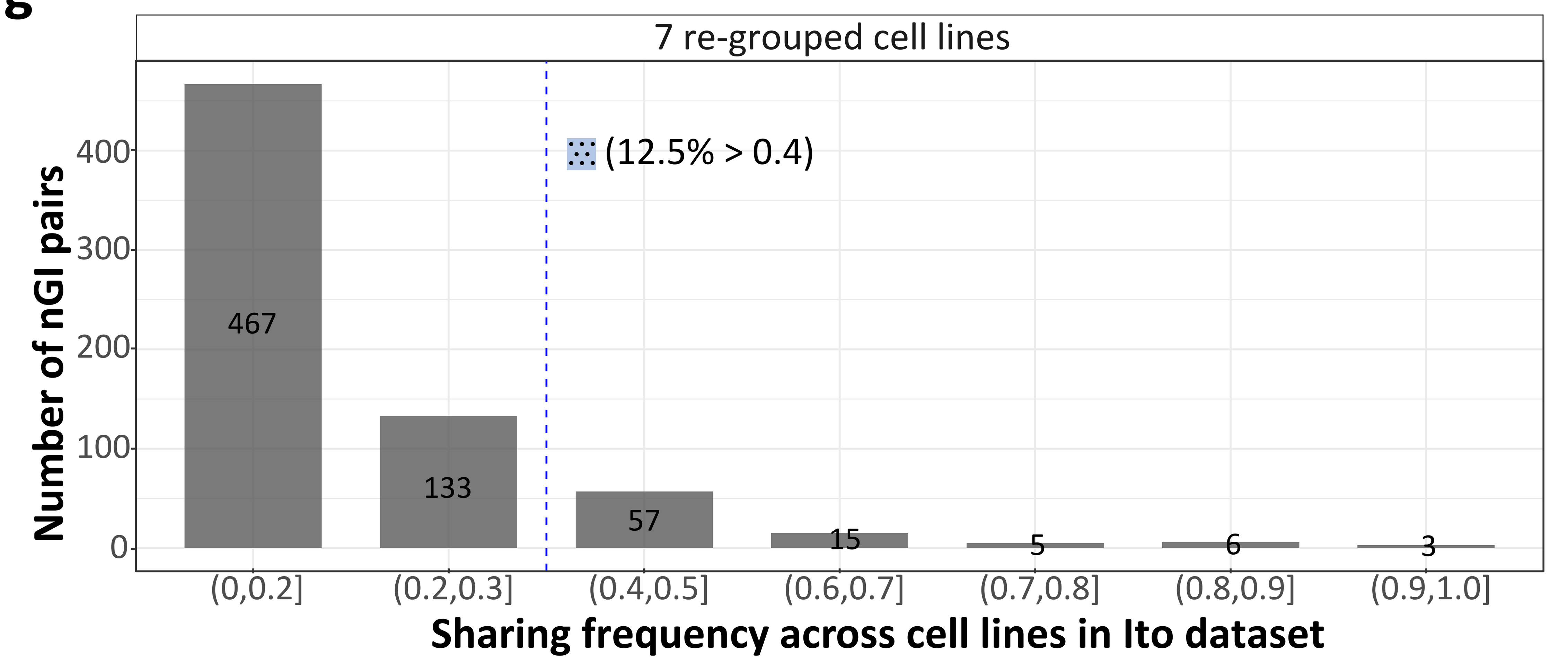

h

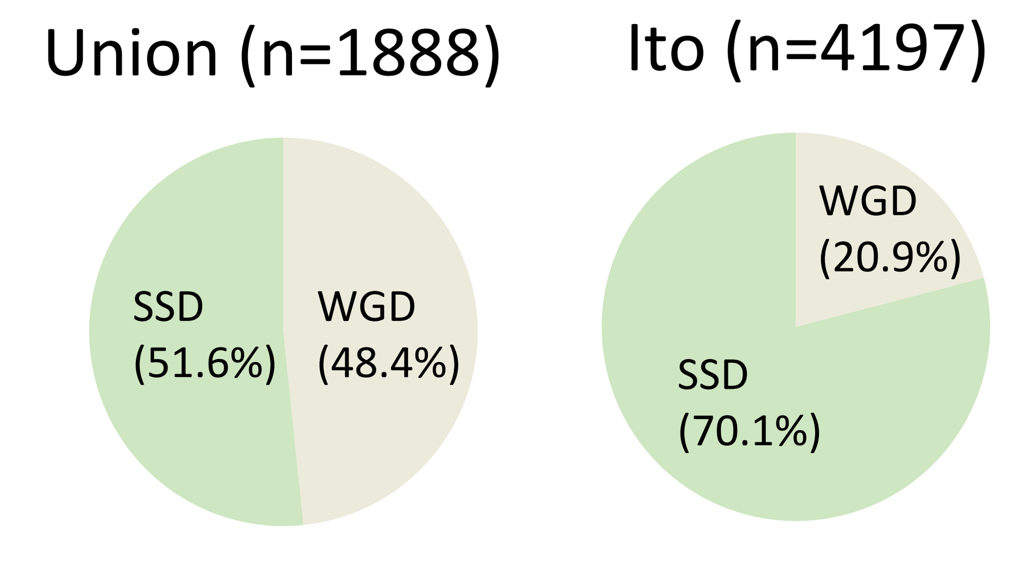

Supplement: Supplementary file 1 [file nwag565_supplemental_files.zip › Supplementary Figures/Supplementary Figure2.pdf]
