# Supplementary material for: Moderate prevalence and evolutionary causes of paralogous compensation in humans
Source: Natl Sci Rev. 2026 Sep 3;13(19):nwag565. doi: 10.1093/nsr/nwag565 (PMC13715443; doi:10.1093/nsr/nwag565)
Supplement: Supplementary file 1 [file nwag565_supplemental_files.zip › Supplementary Figures/Supplementary Figure3.pdf]

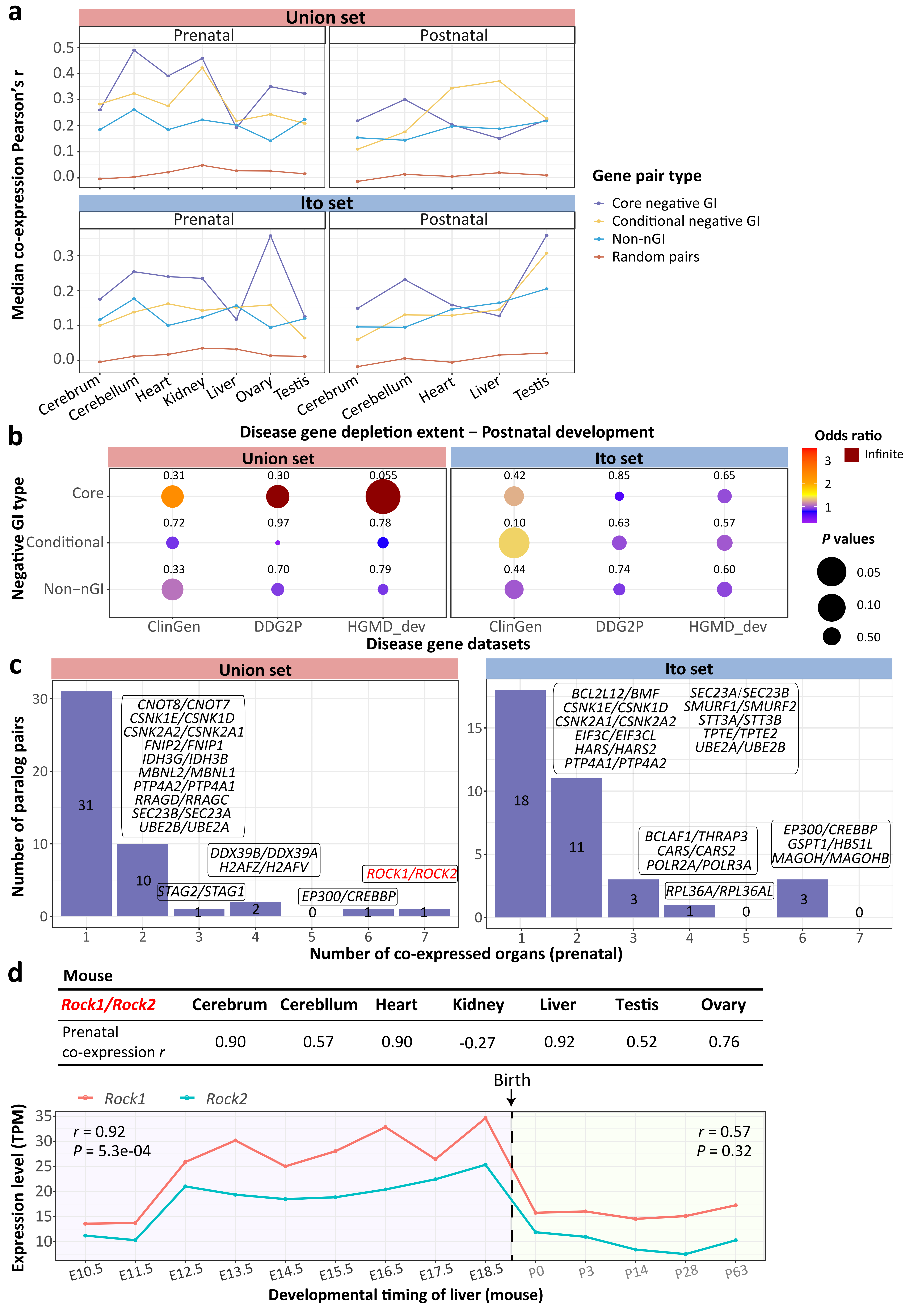
a
Union set
Prenatal
Postnatal
Ito set
Prenatal
Postnatal
Median co-expression Pearson's r
0.5
0.4
0.3
0.2
0.1
0.0
Cerebrum
Cerebellum
Heart
Kidney
Liver
Ovary
Testis
Cerebrum
Cerebellum
Heart
Liver
Testis
Gene pair type
Core negative GI
Conditional negative GI
Non-nGI
Random pairs
b
Disease gene depletion extent – Postnatal development
Union set
Ito set
Negative GI type
Core
Conditional
Non–nGI
0.31
0.30
0.055
0.42
0.85
0.65
0.72
0.97
0.78
0.10
0.63
0.57
0.33
0.70
0.79
0.44
0.74
0.60
ClinGen
DDG2P
HGMD_dev
ClinGen
DDG2P
HGMD_dev
Disease gene datasets
Odds ratio
Infinite
3
2
1
P values
0.05
0.10
0.50
c
Union set
Ito set
Number of paralog pairs
31
10
1
2
0
1
1
CNOT8/CNOT7
CSNK1E/CSNK1D
CSNK2A2/CSNK2A1
FNIP2/FNIP1
IDH3G/IDH3B
MBNL2/MBNL1
PTP4A2/PTP4A1
RRAGD/RRAGC
SEC23B/SEC23A
UBE2B/UBE2A
STAG2/STAG1
DDX39B/DDX39A
H2AFZ/H2AFV
EP300/CREBBP
ROCK1/ROCK2
18
11
3
1
0
3
0
BCL2L12/BMF
CSNK1E/CSNK1D
CSNK2A1/CSNK2A2
EIF3C/EIF3CL
HARS/HARS2
PTP4A1/PTP4A2
SEC23A/SEC23B
SMURF1/SMURF2
STT3A/STT3B
TPTE/TPTE2
UBE2A/UBE2B
BCLAF1/THRAP3
CARS/CARS2
POLR2A/POLR3A
RPL36A/RPL36AL
EP300/CREBBP
GSPT1/HBS1L
MAGOH/MAGOHB
Number of co-expressed organs (prenatal)
d
Mouse
Rock1/Rock2
Cerebrum
Cerebllum
Heart
Kidney
Liver
Testis
Ovary
Prenatal co-expression r
0.90
0.57
0.90
-0.27
0.92
0.52
0.76
Birth
Rock1
Rock2
r = 0.92
P = 5.3e-04
r = 0.57
P = 0.32
Expression level (TPM)
E10.5
E11.5
E12.5
E13.5
E14.5
E15.5
E16.5
E17.5
E18.5
P0
P3
P14
P28
P63
Developmental timing of liver (mouse)
