# Supplementary material for: Moderate prevalence and evolutionary causes of paralogous compensation in humans
Source: Natl Sci Rev. 2026 Sep 3;13(19):nwag565. doi: 10.1093/nsr/nwag565 (PMC13715443; doi:10.1093/nsr/nwag565)
Supplement: Supplementary file 1 [file nwag565_supplemental_files.zip › Supplementary Figures/Supplementary Figure4.pdf]

a

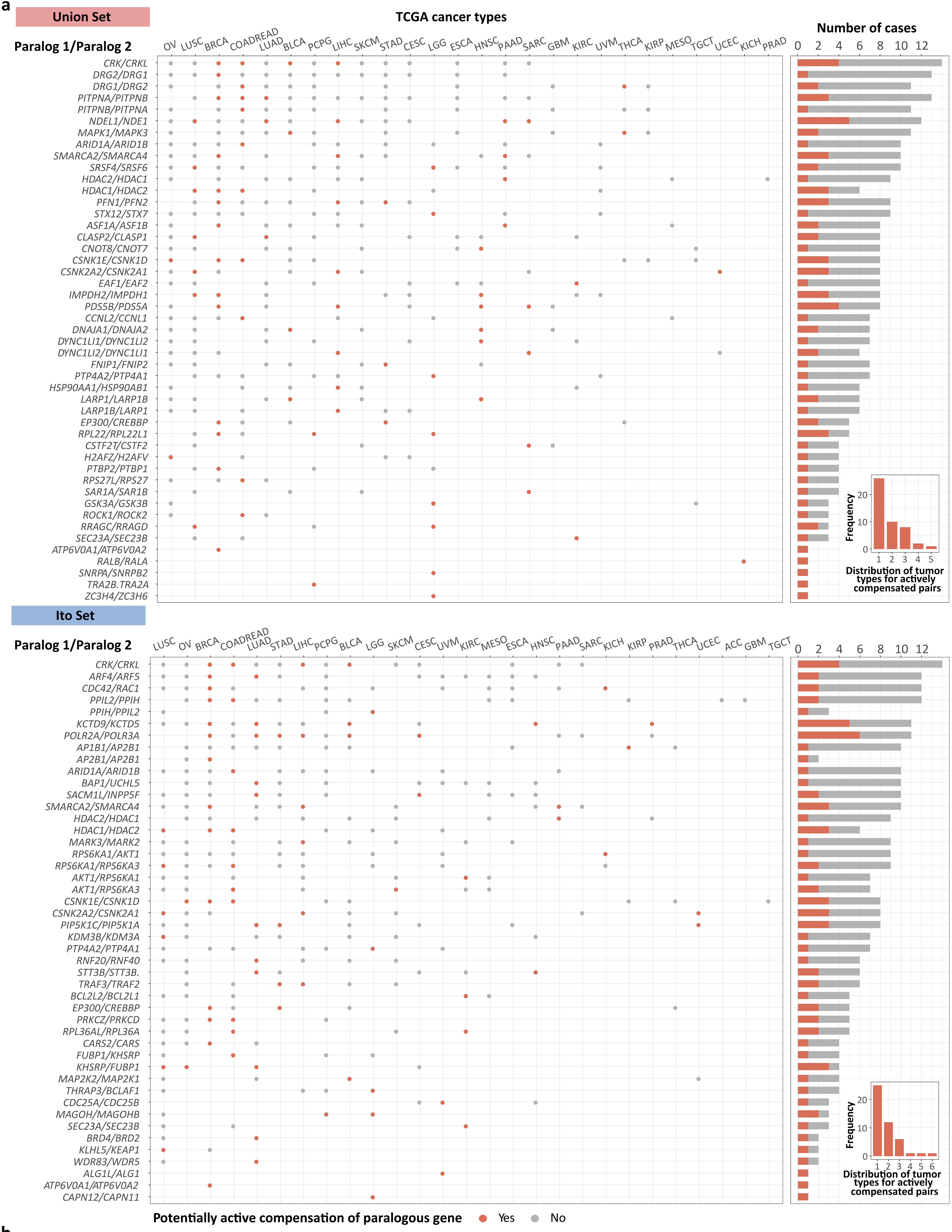

b

Paralog 1 (CN loss)	Paralog 2 (Upregulated)	Dataset	Tumor types		FDRs	
			Active compensation	Mutual exclusivity	Active compensation	Mutual exclusivity
RPL22	*RPL22L1*	Union	LGG	LGG	0.0e+00	7.6e-02
RPL22	*RPL22L1*	Union	BRCA	-	9.9e-09	-
RPL22	*RPL22L1*	Union	PCPG	-	1.1e-05	-
RRAGC	*RRAGD*	Union	LGG	LGG	4.0e-03	1.4e-04
RRAGC	*RRAGD*	Union	LUSC	-	2.3e-02	-
STX12	*STX7*	Union	LGG	LGG	3.1e-02	8.9e-06
PTP4A2	*PTP4A1*	Union & Ito	LGG	LGG	1.6e-08	4.5e-02
THRAP3	*BCLAF1*	Ito	LGG	LGG	4.6e-02	1.0e-06
PPIH	*PPIL2*	Ito	LGG	LGG	1.0e-02	5.6e-04
