# Supplementary material for: Moderate prevalence and evolutionary causes of paralogous compensation in humans
Source: Natl Sci Rev. 2026 Sep 3;13(19):nwag565. doi: 10.1093/nsr/nwag565 (PMC13715443; doi:10.1093/nsr/nwag565)
Supplement: Supplementary file 1 [file nwag565_supplemental_files.zip › Supplementary Figures/Supplementary Figure5.pdf]

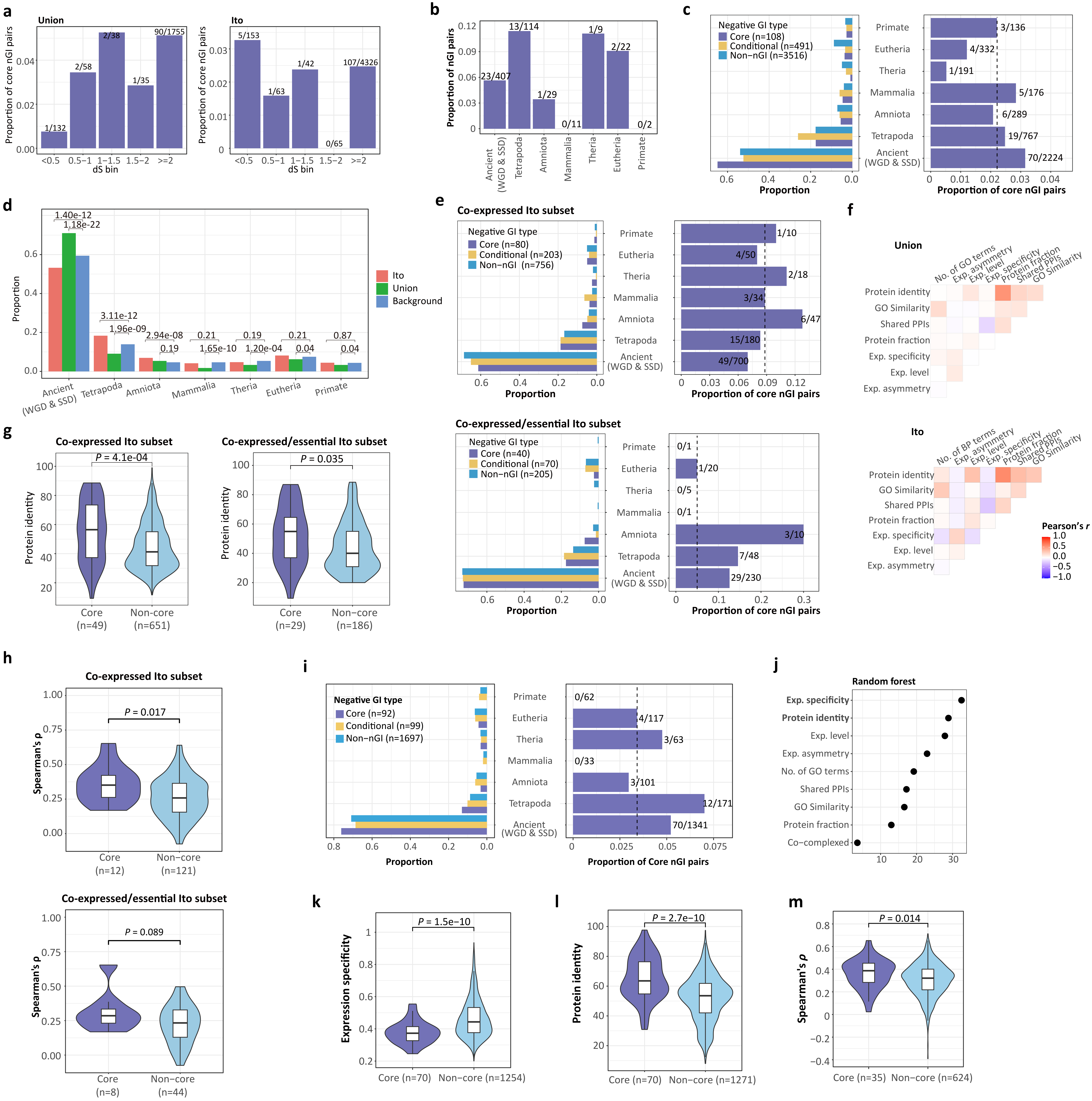

a
Union
Proportion of core nGI pairs
1/132
2/58
2/38
1/35
90/1755
<0.5
0.5−1
1−1.5
1.5−2
>=2
dS bin
Ito
5/153
1/63
1/42
0/65
107/4326
b
Proportion of nGI pairs
23/407
13/114
1/29
0/11
1/9
2/22
0/2
Ancient (WGD & SSD)
Tetrapoda
Amniota
Mammalia
Theria
Eutheria
Primate
c
Negative GI type
Core (n=108)
Conditional (n=491)
Non−nGI (n=3516)
Proportion
3/136
4/332
1/191
5/176
6/289
19/767
70/2224
Proportion of core nGI pairs
d
1.40e-12
1.18e-22
3.11e-12
1.96e-09
2.94e-08
0.19
0.21
1.65e-10
0.19
1.20e-04
0.21
0.04
0.87
0.04
Ito
Union
Background
Proportion
e
Co-expressed Ito subset
Negative GI type
Core (n=80)
Conditional (n=203)
Non−nGI (n=756)
1/10
4/50
2/18
3/34
6/47
15/180
49/700
Co-expressed/essential Ito subset
Core (n=40)
Conditional (n=70)
Non−nGI (n=205)
0/1
1/20
0/5
0/1
3/10
7/48
29/230
f
Union
No. of GO terms
Exp. asymmetry
Exp. level
Exp. specificity
Protein fraction
Shared PPIs
GO Similarity
Protein identity
Ito
No. of BP terms
Pearson's r
g
Co-expressed Ito subset
P = 4.1e-04
Protein identity
Core (n=49)
Non−core (n=651)
Co-expressed/essential Ito subset
P = 0.035
Core (n=29)
Non−core (n=186)
h
Co-expressed Ito subset
P = 0.017
Spearman's ρ
Core (n=12)
Non−core (n=121)
Co-expressed/essential Ito subset
P = 0.089
Core (n=8)
Non−core (n=44)
i
Negative GI type
Core (n=92)
Conditional (n=99)
Non−nGI (n=1697)
0/62
4/117
3/63
0/33
3/101
12/171
70/1341
Proportion of Core nGI pairs
j
Random forest
Exp. specificity
Protein identity
Exp. level
Exp. asymmetry
No. of GO terms
Shared PPIs
GO Similarity
Protein fraction
Co−complexed
k
P = 1.5e-10
Expression specificity
Core (n=70)
Non−core (n=1254)
l
P = 2.7e−10
Protein identity
Core (n=70)
Non−core (n=1271)
m
P = 0.014
Spearman's ρ
Core (n=35)
Non−core (n=624)
