# Supplementary material for: Moderate prevalence and evolutionary causes of paralogous compensation in humans
Source: Natl Sci Rev. 2026 Sep 3;13(19):nwag565. doi: 10.1093/nsr/nwag565 (PMC13715443; doi:10.1093/nsr/nwag565)
Supplement: Supplementary file 1 [file nwag565_supplemental_files.zip › Supplementary Figures/Supplementary Figure6.pdf]

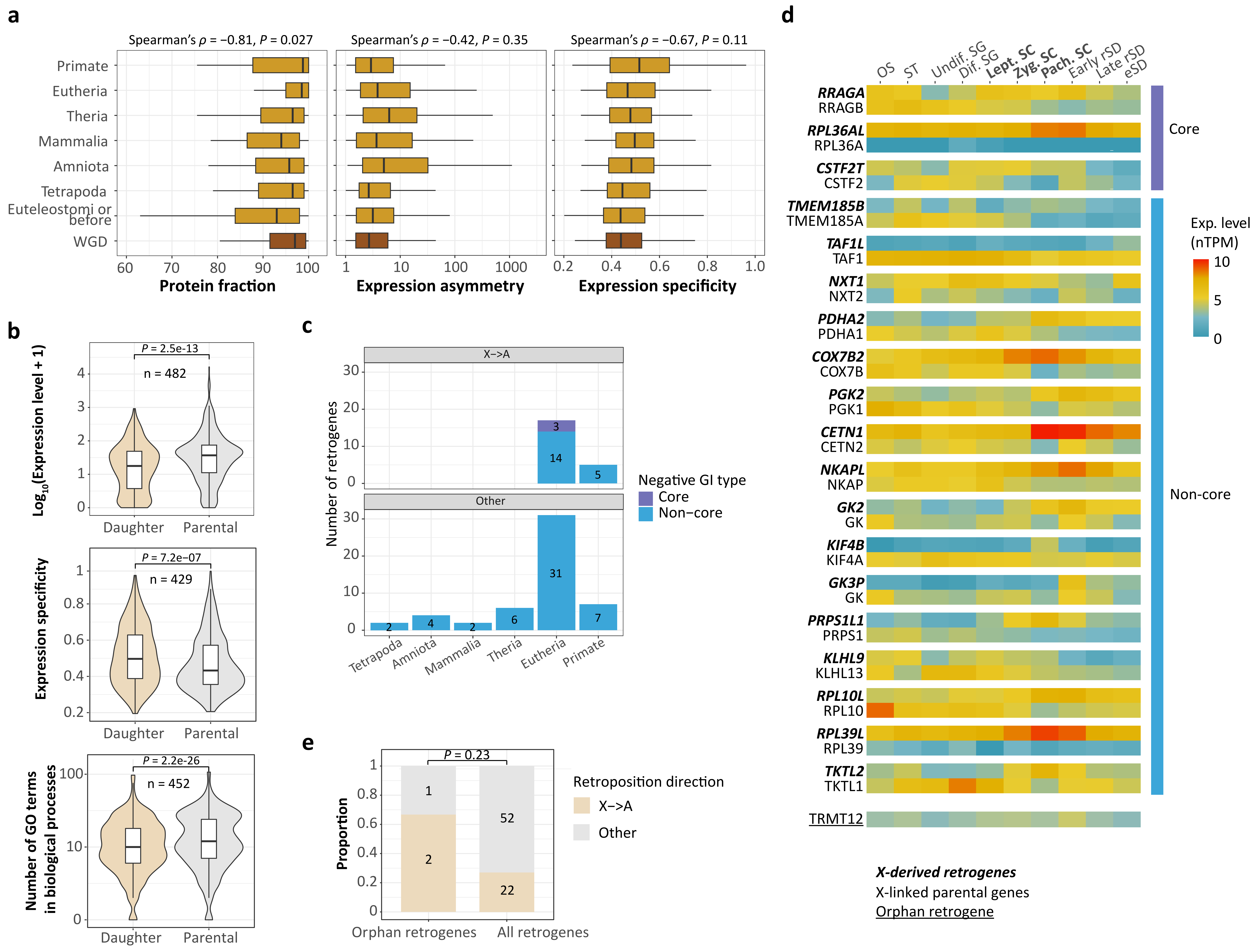
a
Spearman's ρ = −0.81, P = 0.027
Spearman's ρ = −0.42, P = 0.35
Spearman's ρ = −0.67, P = 0.11
Primate
Eutheria
Theria
Mammalia
Amniota
Tetrapoda
Euteleostomi or before
WGD
Protein fraction
Expression asymmetry
Expression specificity
b
Log10(Expression level + 1)
P = 2.5e-13
n = 482
Expression specificity
P = 7.2e−07
n = 429
Number of GO terms in biological processes
P = 2.2e-26
n = 452
Daughter
Parental
c
Number of retrogenes
X−>A
Other
Negative GI type
Core
Non−core
Tetrapoda
Amniota
Mammalia
Theria
Eutheria
Primate
d
OS
ST
Undif. SG
Dif. SG
Lept. SC
Zyg. SC
Pach. SC
Early rSD
Late rSD
eSD
RRAGA
RRAGB
RPL36AL
RPL36A
CSTF2T
CSTF2
TMEM185B
TMEM185A
TAF1L
TAF1
NXT1
NXT2
PDHA2
PDHA1
COX7B2
COX7B
PGK2
PGK1
CETN1
CETN2
NKAPL
NKAP
GK2
GK
KIF4B
KIF4A
GK3P
GK
PRPS1L1
PRPS1
KLHL9
KLHL13
RPL10L
RPL10
RPL39L
RPL39
TKTL2
TKTL1
TRMT12
Core
Non-core
Exp. level (nTPM)
X-derived retrogenes
X-linked parental genes
Orphan retrogene
e
Proportion
P = 0.23
Orphan retrogenes
All retrogenes
Retroposition direction
X−>A
Other
