# Supplementary material for: Moderate prevalence and evolutionary causes of paralogous compensation in humans
Source: Natl Sci Rev. 2026 Sep 3;13(19):nwag565. doi: 10.1093/nsr/nwag565 (PMC13715443; doi:10.1093/nsr/nwag565)
Supplement: Supplementary file 1 [file nwag565_supplemental_files.zip › Supplementary Figures/Supplementary Figure7.pdf]

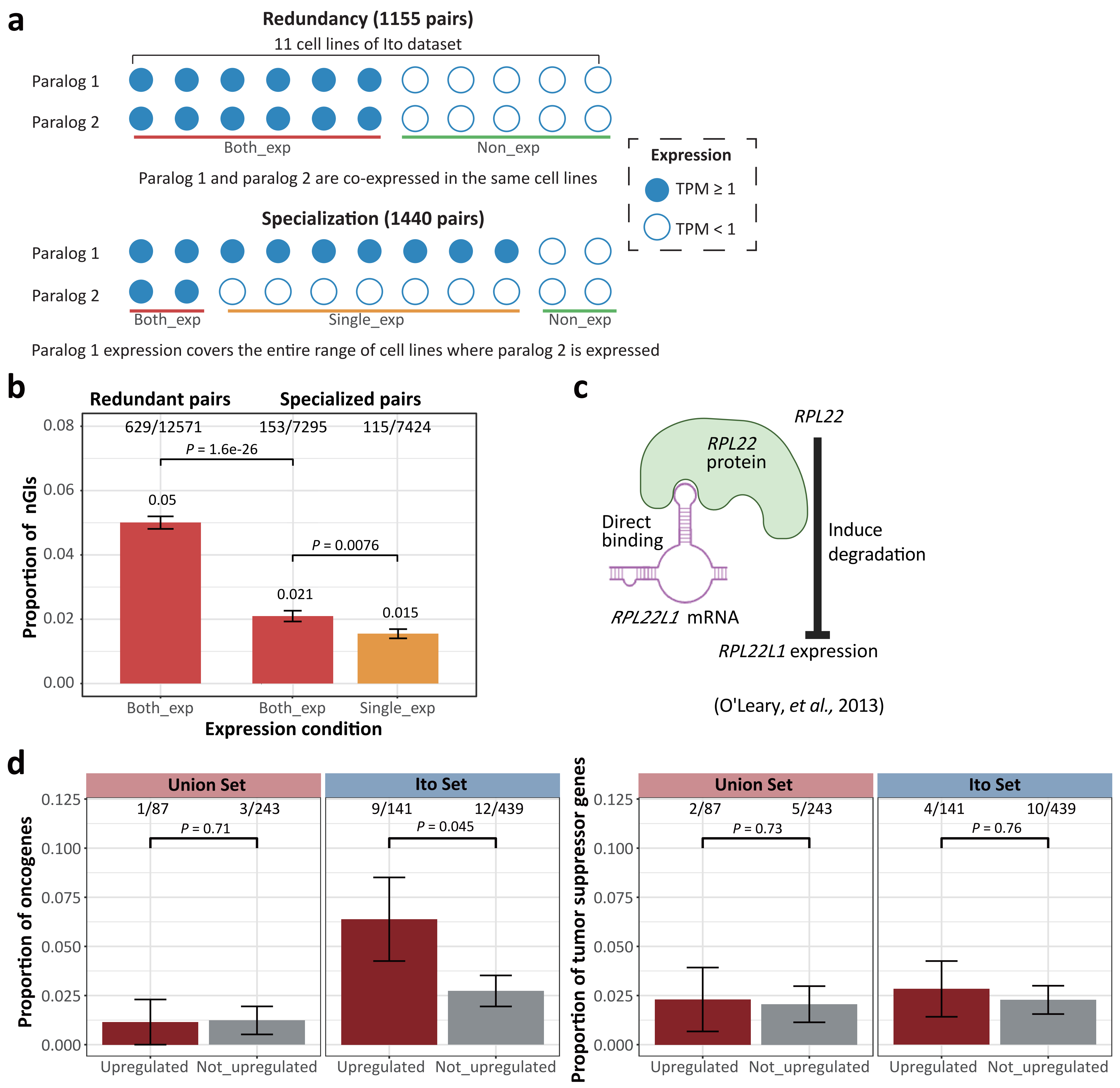
a
Redundancy (1155 pairs)
11 cell lines of Ito dataset
Paralog 1
Paralog 2
Both_exp
Non_exp
Paralog 1 and paralog 2 are co-expressed in the same cell lines
Specialization (1440 pairs)
Paralog 1
Paralog 2
Both_exp
Single_exp
Non_exp
Paralog 1 expression covers the entire range of cell lines where paralog 2 is expressed
Expression
TPM ≥ 1
TPM < 1
b
Redundant pairs
Specialized pairs
629/12571
153/7295
115/7424
P = 1.6e-26
P = 0.0076
0.05
0.021
0.015
Proportion of nGIs
0.08
0.06
0.04
0.02
0.00
Both_exp
Both_exp
Single_exp
Expression condition
c
RPL22
RPL22 protein
Direct binding
RPL22L1 mRNA
Induce degradation
RPL22L1 expression
(O'Leary, et al., 2013)
d
Union Set
Ito Set
1/87
3/243
P = 0.71
9/141
12/439
P = 0.045
Proportion of oncogenes
0.125
0.100
0.075
0.050
0.025
0.000
Upregulated
Not_upregulated
Union Set
Ito Set
2/87
5/243
P = 0.73
4/141
10/439
P = 0.76
Proportion of tumor suppressor genes
