# Supplementary material for: Moderate prevalence and evolutionary causes of paralogous compensation in humans
Source: Natl Sci Rev. 2026 Sep 3;13(19):nwag565. doi: 10.1093/nsr/nwag565 (PMC13715443; doi:10.1093/nsr/nwag565)
Supplement: Supplementary file 1 [file nwag565_supplemental_files.zip › Supplementary Information.docx]

**Title: Moderate prevalence and evolutionary causes of paralogous compensation in humans**

**Authors:** Jieyu Shen^1,2^†, Daqi Yu^1,2$^†*, Wenjia Wang^3#^†, Tianhan Su^1,2^†, Dingding Wang^3^^, Junbo Liang^3^, Yiqiong Guo^4^, Shangqi Zhao^4^, Huijing Ma^1^, Jichang Wang^5,6^, Xiaoyue Wang^4^*, Yong E. Zhang^1,2^*

**Affiliations:**

^1^ State Key Laboratory of Animal Biodiversity Conservation and Integrated Pest Management, Institute of Zoology, Chinese Academy of Sciences; Beijing 100101, China.

^2^ University of Chinese Academy of Sciences; Beijing 100049, China.

^3^ State Key Laboratory of Common Mechanism Research for Major Diseases, Department of Biochemistry and Molecular Biology, Institute of Basic Medical Sciences & School of Basic Medicine, Chinese Academy of Medical Sciences & Peking Union Medical College, Beijing 100005, China.

^4^ State Key Laboratory of Complex, Severe, and Rare Diseases; Center for Bioinformatics, National Infrastructures for Translational Medicine, Institute of Clinical Medicine, Peking Union Medical College Hospital, Chinese Academy of Medical Sciences & Peking Union Medical College, Beijing 100730, China.

^5^ Key Laboratory for Stem Cells and Tissue Engineering (Sun Yat-sen University), Ministry of Education, Guangzhou 510080, China.

^6^ Department of Histology and Embryology, Zhongshan School of Medicine, Sun Yat-sen University, Guangzhou 510080, China.

†These authors contributed equally to this work.

*Corresponding author. Email: [zhangyong@ioz.ac.cn](mailto:zhangyong@ioz.ac.cn), [wxy@ibms.pumc.edu.cn](mailto:wxy@ibms.pumc.edu.cn), [withydq@163.com](mailto:withydq@163.com).

^$^Current address: Beijing AstraGenomics Co., LTD, Beijing 100094, China.

^#^Current address: Department of Clinical Laboratory, Shandong Provincial Hospital Affiliated to Shandong First Medical University, Jinan 250021, China.

^^^Current address: JST Sarcopenia Research Centre, Beijing Research Institute of Traumatology and Orthopaedics, National Center for Orthopaedics, Beijing Jishuitan Hospital, Capital Medical University, Beijing 100035, China.

This file contains supplementary figures, supplementary methods, supplementary note, supplementary references, and supplementary note figures. Supplementary Methods provide detailed descriptions and additional details of the MATERIALS AND METHODS presented in the main text. Supplementary note consists of experimental and computational details for the first and fourth sections of the Results, focusing primarily on the details of self-generated digenic CRISPR screening and RNAi validation experiment.

Index

[Supplementary Figures	4](#_Toc238747586)

[Supplementary Methods	16](#_Toc238747587)

[Generation of an association dataset comprising 386 pairs	16](#_Toc238747588)

[Feature enrichment analyses across CRISPR screening datasets	16](#_Toc238747589)

[Transcriptome clustering across cell lines	17](#_Toc238747590)

[Construction and characterization of the Union and Ito datasets	17](#_Toc238747591)

[Co-expression analyses during early development	18](#_Toc238747592)

[Depletion analyses of disease-related genes in prenatal co-expressed paralogs	19](#_Toc238747593)

[Active compensation analysis in tumor samples	19](#_Toc238747594)

[Mutual exclusivity analysis of paralog pairs in tumor samples	20](#_Toc238747595)

[RNAi experiments	21](#_Toc238747596)

[Entanglement analysis	21](#_Toc238747597)

[Expression and sequence feature analyses	22](#_Toc238747598)

[Co-expressed and co-expressed/essential Ito subset	23](#_Toc238747599)

[Cell-line-level specialization analysis	23](#_Toc238747600)

[Retrogene analyses	24](#_Toc238747601)

[Supplementary Note	25](#_Toc238747602)

[Design of sgRNA sequences	25](#_Toc238747603)

[Specification of sgRNA pairs	26](#_Toc238747604)

[Overall structure of the screening library and sampling of 687 duplicate pairs	27](#_Toc238747605)

[Cell culture, lentivirus production and sgRNA plasmid cloning	28](#_Toc238747606)

[Library construction, CRISPR screening and sequencing	29](#_Toc238747607)

[Quantification of GIs based on dependency scores	31](#_Toc238747608)

[Analysis of screening data using the inverted variance weighting method	33](#_Toc238747609)

[Estimation of dual editing efficiency	37](#_Toc238747610)

[Experimental validations of negative GI status for 17 gene pairs	37](#_Toc238747611)

[Quality control of data generation and analysis algorithm	39](#_Toc238747612)

[The RNAi experiment design	40](#_Toc238747613)

[Supplementary References	44](#_Toc238747614)

[Supplementary Note Figures	49](#_Toc238747615)

Supplementary Figures

**Supplementary Figure. 1 Evaluation of public and purpose-built dual CRISPR knockout datasets. a** Quality assessment across studies. Our study is labeled as “This work,” and Gonatopoulos-Pournatzis *et al.* is abbreviated as GP *et al.* For each study, “-” and “unreported” indicate whether a specific evaluation was “not performed” and “performed without reported statistics”, respectively. Detailed evaluations of this work are shown in Supplementary Note Figs. 2-4. For off-target controls in this work, refer to panel **b** and the Supplementary Note. **b** Procedures for constructing a dual-targeting system. Off-target criteria for sgRNA design are included. **c** Library design. The library contains 11,614 sgRNA pairs from nine groups. Positive control Set1 and Set2 include negative genetic interaction (nGI) gene pairs extracted from the literature. These pairs primarily do not originate from HCT116 or GM12878 cells and thus serve as approximate controls. “Positive control genes” refer to single-gene knockouts targeting genes included in positive control Set1 and Set2. The number “1,348” is not precisely twice “687” due to some pairs sharing one paralog. **d** Proportion of negative GI pairs identified in the association dataset across five studies. Dashed bars indicate the overall negative GI rate estimated by the proportion of nGI pairs identified in at least one cell line relative to the total number of paralog pairs screened in each individual dataset, with the top number representing the odds ratio, denoting the enrichment fold relative to the overall proportion. Error bars show one standard error (s.e.) under a binomial distribution. **e** Enrichment tests of molecular features in duplicate pairs with negative GIs across five studies. PPI refers to protein-protein interaction. *P* values are calculated using Fisher’s exact test (one-sided, two left columns) and the Wilcoxon rank-sum test (two-sided, three right columns).

**Supplementary Figure. 2 Cell line diversity and generation of the Union and Ito datasets.** **a Phylogenetic distribution of 17 cancer cell lines and 2 relatively normal cell lines based on transcriptome data.** The outer layer depicts the proportion of negative GI pairs for each cell line. Shared cell lines from different studies are labeled separately. **b** Proportion of negative GI retrieved from two large-scale surveys [1, 2] of random gene pairs in human cell lines. Error bars show one standard error (s.e.) under a binomial distribution. **c** Association between paralog expression status and nGIs in screened cell lines. In each cell line, paralog pairs were classified and counted according to whether neither, one or both genes were expressed (Non_exp, Single_exp and Both_exp, respectively). The proportions of these expression categories were compared between pairs with (Y) and without (N) nGIs in the two datasets. *P* values were calculated using chi-squared tests. **d** Distribution of the proportion of nGI detected after downsampling to different numbers of cell lines in the Union (left) and Ito (right) datasets. Each point represents a unique combination of sampled cell lines at a given sampling size. The black curve shows a locally weighted regression fit generated by the “loess” method, and the shaded area indicates the standard error (confidence band) around the fitted curve. **e Cancer cell line regrouping based on tissue origin in the Ito dataset. f-g Distribution of negative GI pair-sharing frequency across cell lines in the Union (f) and Ito (g) datasets.** Sharing frequency is defined as the proportion of cell lines in which a gene pair exhibits a negative GI, relative to the total number of assayed cell lines. Note that the frequency bins increase with the total number of cell lines. **h Proportion of SSD and WGD pairs in the Union and Ito datasets.**

**Supplementary Figure. 3 Developmental co-expression of paralog pairs. a** Median co-expression levels of paralog pairs across different nGI groups during human prenatal and postnatal development. The right panel shows data from five of seven organs with sufficient postnatal samples (≥ 5). **b** Depletion status of postnatally co-expressed paralogs among disease-associated genes (ClinGen, DDG2P, HGMD development). Odds ratios compare disease-gene enrichment in non-co-expressed *vs.* co-expressed pairs; one-sided Fisher’s exact *P* values are shown. **c** Distribution of the number of organs in which core nGI pairs are co-expressed. Pairs co-expressed in at least two organs are labeled on bars. **d** Prenatal co-expression of *Rock1* and *Rock2* in mice across organs. The Pearson correlation of prenatal expression is shown, along with the expression dynamics of *Rock1* and *Rock2* during mouse liver development. TPM (transcripts per million) values are used to quantify expression levels. Pearson’s *r* and corresponding *P* values are displayed separately for prenatal (magenta background) and postnatal (yellow background) stages. "E" and "P" indicate embryonic days and postnatal days, respectively, during mouse development.

**Supplementary Figure. 4** **Paralog pairs with active compensation in Union and Ito datasets, and** **pairs exhibiting both active compensation and mutual exclusivity. a** Paralog pairs with core negative GI relationships and active compensation signals in at least one cancer type. A total of 47 and 46 duplicate pairs are shown for the Union and Ito datasets, respectively. Cases with high paralog 1 copy loss are labelled as dots, where active compensation is indicated in red, and others are shown in grey. For each paralog pair, the gene before the slash denotes the paralog with copy number loss, whereas the gene after the slash denotes the paralog tested for compensatory upregulation. Genes and cancer types are sorted by the total number of cases. Inset histograms display the distribution of cancer types per paralog pair. **b** Core negative GI pairs significant in both active compensation and mutual exclusivity analyses within the same tumor types. “-” indicates non-significance in the corresponding columns. Multiple testing correction was performed via false discovery rate (FDR).

**Supplementary Figure. 5 Extended analyses of pleiotropy and dosage subfunctionalization models. a** Proportion of core negative GI pairs across $d_{S}$ bins in the Union (left) and Ito (right) datasets. **b** Proportion of core negative GI pairs within each age group from this study. **c** Distribution of duplication age groups for Ito paralog pairs with CDS identity < 90%. **d** Comparison of duplication age distributions between either the Union or Ito dataset and genomic background. **e** Distribution of duplication age groups in the 11-cell-line co-expressed Ito subset (top) and the further restricted subset with essential mouse orthologs (bottom). **f** Correlations of sequence and function features of duplicate genes in Union (left) and Ito (right) dataset. **g** Distribution of protein identity for ancient duplicate pairs in the 11-cell-line co-expressed Ito subset (left) and the further restricted subset with essential mouse orthologs (right). **h** Distribution of paralogous correlations in evolutionary rates (*K*_a_/*K*_s_) across nGI types in the 11-cell-line co-expressed Ito subset (top) and the further restricted subset with essential mouse orthologs (bottom). **i** Distribution of duplication age groups in the Union dataset. **j** Feature importance evaluation for predicting paralog pairs in the Union dataset as core negative GI pairs using a random forest model. **k-l** Distribution of expression specificity (**k**) and protein identity (**l**) for ancient duplicate gene pairs. **m** Distribution of paralogous correlation in evolutionary rates (*K*_a_/*K*_s_) across different nGI types. Panels **i-m** are based on the Union dataset. The figure conventions for Panels **i-m** follow those of Fig. 5**b** and Fig. 5**d-g**, respectively.

**Supplementary Figure. 6 Extended analyses of SSD pairs and out-of-X retrogenes. a** Age-correlated distribution of protein fraction, expression asymmetry, and expression specificity of SSD gene pairs. **b** Distribution of expression and functional features of daughter and parental genes. **c** Duplication age group distribution of retroposed pairs. **d** Expression levels of 22 X-derived retrogenes and their X-linked parental genes across various testis cell types. Cell types included Sertoli cells (ST); undifferentiated and differentiated spermatogonia (undiff. SG and diff. SG); leptotene, zygotene, and pachytene spermatocytes (lept. SC, zyg. SC, pach. SC; all in bold); round spermatids (rSD); elongated spermatids (eSD); and other somatic cells (OS). For the two X-derived orphan retrogenes, *CHMP1B* and *TRMT12*, only the expression of *TRMT12* has been reported in the dataset. “nTPM” represents normalized TPM. **e** Proportion of X-derived retrogenes among orphan retrogenes and all retrogenes.

**Supplementary Figure. 7 Extended upregulation analyses and additional analyses for evolutionary theories.** **a** Schematic illustration of cell line level redundant and specialized paralog pairs. **b** Proportions of nGI events across Both_exp and Single_exp contexts for dosage-redundant and dosage-specialized pairs. Colors correspond to the expression categories defined in panel **a**. *P* values were calculated using one-sided Fisher’s exact tests. **c** The mechanism of active compensation between *RPL22* and *RPL22L1*. This panel is adapted from O’Leary *et al*. **d** Proportion of oncogenes and tumor suppressor genes in upregulated or non-upregulated paralog (paralog 2). *P* values are calculated by Fisher’s exact test (one-sided).

Supplementary Methods

Generation of an association dataset comprising 386 pairs
To validate our experimental findings against existing knowledge and to aid in the classification of negative genetic interactions (nGIs), we compiled a reference "association dataset" of predicted negative GIs. This dataset was generated by merging two previously published lists derived from large-scale essentiality screens. The first, from De Kegel *et al.*, identified 374 paralog pairs, where lower expression of one paralog correlated with higher cellular essentiality of the other across numerous cancer cell lines [3]. The second, from Dede *et al.*, identified 66 pairs where a loss-of-function mutation in one paralog correlated with higher essentiality of the other [4]. After merging these two lists and removing redundant entries, we obtained a final set of 386 unique paralog pairs. This dataset served as an independent benchmark for assessing the quality of experimental screens and as a key criterion for defining core negative GI pairs in our classification scheme.

Feature enrichment analyses across CRISPR screening datasets
To characterize the paralog pairs in our study and compare them across different datasets, we compiled a comprehensive set of molecular and functional features from various public databases. A dataset of annotated human protein complexes was downloaded from the CORUM 3.0 database [5] to determine co-complex membership. All annotated human paralogs and their protein sequence identities were retrieved from Ensembl BioMart (version 96 or v96) [6]; only pairs with a mean identity greater than 20% were included in our analyses. Gene family assignments were obtained from PantherDB (version 17.0) [7]. Pairwise protein-protein interaction (PPI) data were retrieved from BioPlex 3.0 [8], and the shared PPI ratio was calculated for pairs where each gene had at least three interaction partners. To quantify functional similarity, we calculated the semantic similarity of Gene Ontology (GO) annotations using the "BMA" method in the GOSemSim R package [9], excluding less reliable electronic annotations (IEA, ISS, NAS). Feature-enrichment analyses used binomial tests for proportional data (enrichment of association-derived pairs), Wilcoxon rank-sum tests for continuous variables, and Fisher’s exact tests for categorical variables, including cross-dataset concordance and comparisons between nGI and non-nGI pairs. For association-derived pairs, the baseline was the overall nGI proportion in each dataset, defined as the fraction of screened paralog pairs exhibiting an nGI in at least one cell line (dashed line in Fig. S1d).

Transcriptome clustering across cell lines
For visualizing the functional relationships among the 19 cell lines included in our integrated analysis, we performed clustering based on their transcriptomic profiles. Transcriptome quantification data (TPM values) for the 17 cancer cell lines were retrieved from the Broad Institute's Project Achilles (<https://depmap.org/portal/>, 2021Q3 release). Transcriptome data for the two normal cell lines, GM12878 and RPE1, were obtained from the NCBI Gene Expression Omnibus (GEO) under accession numbers GSE30400 and GSE89413, respectively. For the analysis, we retained only genes with a TPM value greater than 1 in more than half of the cell lines. To provide a broader context for the clustering, we included an additional 325 cell lines randomly sampled from the full Project Achilles dataset. Hierarchical clustering was performed using the "Average" linkage method implemented in the ape R package (version 5.5) [10]. The resulting phylogenetic tree was visualized and annotated using the ggtree (version 3.10.0) [11]. GgtreeExtra (version 1.12.0) [12] package was used to display the negative GI rates for each cell line.

Construction and characterization of the Union and Ito datasets
To facilitate robust and parallel analyses that account for the distinct characteristics of the available datasets, we organized the five selected experimental screens into two main resources. The Union dataset was created as a non-redundant compilation of the 2,168 unique pairs from the four studies: Dede *et al.* [4], Parrish *et al.* [13], Thompson *et al.* [14], and the new data generated in this work. The Ito dataset consisted solely of the 4,859 paralog pairs screened by Ito *et al.* [15], which has a focus on enzymatic paralogs. To classify the robustness of the observed genetic interactions, we categorized pairs in each dataset as core negative GI, conditional negative GI, or non-nGI based on the prevalence of negative GI signals. For the Union dataset, core GIs were defined as pairs showing negative GIs in at least 50% of the assayed cell lines or being present in the aforementioned association dataset. For the Ito dataset, the five melanoma-derived cell lines (MeWo, MELJUSO, IPC298, HS936T, and HS944T) were treated as a single cell type, wherein the negative GI was recorded if it showed negative GI in any of these cell lines. To obtain a comparable number of core pairs across Union and Ito datasets, we lowered the core nGI threshold to include pairs present in at least 40% of assayed cell lines or in the association dataset. All other pairs with a negative GI in at least one cell line were classified as conditional nGIs. The left pairs without negative GIs in any cell line were classified as Non-nGI pairs.

GO enrichment analysis was conducted using Metascape [16], focusing exclusively on biological process-related terms. For the Union dataset, Ito dataset, and all paralogs, 3,000 duplicate genes were randomly selected to meet Metascape’s input size limitation.

Co-expression analyses during early development
To investigate co-expression patterns during development, we downloaded raw RNA-seq data from Cardoso-Moreira *et al.* [17], which provides a high-resolution time course of the transcriptome across seven human organs (cerebrum, cerebellum, heart, kidney, liver, ovary, and testis) from embryonic to adult stages. Ovary and kidney were excluded from the postnatal analysis due to an insufficient number (< 5) of data points across postnatal developmental stages. The neonatal period (week 0) was classified as postnatal. RNA-seq samples were quantified individually using Kallisto (version 0.46.0) [18] with the Ensembl transcript annotation. For each organ and developmental stage, gene-expression values were summarized by taking the median across all available biological replicates. To normalize differences in library size and composition across developmental stages, we applied a rank-based normalization method using the top 1,000 genes with the lowest variation in expression rank across the genome [19]. Co-expression was quantified as Pearson’s correlation of the normalized expression profiles for each paralog pair, analyzed separately for prenatal and postnatal periods within each organ. For each organ, only genes with TPM > 1 in at least one stage were analyzed. A pair was deemed significantly co-expressed if each gene was within the top 5% of the other's co-expressed partners. GO enrichment analysis for the sets of significantly co-expressed genes was performed with the Metascape web server [16]. The random expectations are derived from all possible combinations of 4,147 genes in the Union dataset and 3,217 genes in the Ito dataset, respectively.

Depletion analyses of disease-related genes in prenatal co-expressed paralogs
To assess the link between paralogous compensation and human disease, we used three independent, curated datasets of disease-associated genes. These included: 1) the ClinGen dataset [20] (2024 release), filtered to include only 1,997 gene-disease pairs with "definitive", "strong", or "moderate" evidence; 2) the 883 genes in Developmental Disorder panel from the Gene2Phenotype database (DDG2P, version 4.6) [21]; and 3) a curated list of 1,815 developmental disorder genes from Human Gene Mutation Database (HGMD) [22], as initially compiled by Cardoso-Moreira *et al.* [23] and subsequently filtered by us with tags including “DM”, “Developmental”, or “Developmental++”. A paralog pair was defined as disease-related if either gene was present in a given dataset. For each nGI category (core, conditional, non-nGI), we calculated the odds ratio to quantify the depletion of disease-related genes among prenatally co-expressed *vs.* non-co-expressed paralog pairs. Statistical significance of the depletion was assessed using a one-sided Fisher’s exact test.

Active compensation analysis in tumor samples
To search for signals of potentially active compensation in a disease context, we retrieved pan-cancer copy number variation (CNV) and Z-score normalized gene expression data from The Cancer Genome Atlas (TCGA) project, accessed via the cBioPortal [24]. Notably, although TCGA includes 33 cancer types, COAD (Colon adenocarcinoma) and READ (Rectum adenocarcinoma) are merged as COADREAD (Colon adenocarcinoma/Rectum adenocarcinoma) in cBioPortal. For each paralog pair in each of the 32 TCGA tumor types, we tested for active compensation. This was defined as a significant upregulation of one paralog (responsive paralog, paralog 2) in tumor samples where the other paralog (paralog 1) had experienced CN loss (defined as deep or shallow deletion, with GISTIC values of -2 or -1). To ensure sufficient statistical power, this analysis was restricted to paralog 1 with a high rate of CN loss (>30% frequency or ranking among the top 500 most frequently deleted genes) in a given tumor type. Although we sought to test active compensation bidirectionally, some paralog pairs could be tested in only one direction because only one paralog met the minimum copy-number-loss frequency threshold. We performed one-sided t-tests to confirm that focal genes were significantly downregulated (false discovery rate or FDR < 0.001) in samples with CN loss. We then used a one-sided t-test to compare the expression of the responsive paralog in "paralog 1 loss" samples *vs.* "paralog 1 diploid" samples, with significance defined as a multiple testing-corrected FDR < 0.05. Random expectations for active compensation rates were generated by analyzing random pairings of duplicate genes within the same nGI group (core, conditional, non-nGI).

To assess active compensation across tumor types, we binned paralog 1 CN-loss downregulation into 1, 2-6, or 7-14 tumor types (maximum loss observed in 14 tumor types). Conditional on paralog 1 loss, the partner (paralog 2) was classified as cross-tumor (upregulated in > 1 tumor type), tumor-specific (upregulated in exactly 1 tumor type), or not upregulated. Cross-tumor compensation rates were then compared for core, conditional, and non-nGI pairs among paralog sets with cross-tumor paralog 1 CN loss, with significance assessed by one-sided Fisher’s exact tests.

We used COSMIC v98 Cancer Gene Census (CGC) [25] to test oncogene enrichment among responsive paralogs (paralog2). Only genes from core or conditional GI classes annotated as “oncogene” or “tumor suppressor gene (TSG)” were retained. We compared the proportions of oncogenes and TSGs between upregulated and not-upregulated responsive paralogs, assessing significance with one-sided Fisher’s exact test.

Mutual exclusivity analysis of paralog pairs in tumor samples
Using the same TCGA CNV data, we searched for signals of potential mutual exclusivity of gene loss events, which indicates a synthetic lethal relationship. For each paralog pair in each tumor type, we constructed a 2x2 contingency table of loss events (no loss, paralog 1 loss only, paralog 2 loss only, simultaneous loss). A pair was considered to exhibit mutual exclusivity if the number of samples with simultaneous loss was significantly lower than expected under a model of independence, as determined by a one-sided Fisher’s exact test (FDR < 0.1). The analysis was restricted to pairs where both paralogs had a high loss rate (>30% frequency or ranked among the top 500 most frequently deleted genes) and at least one double-loss event was observed across all samples. To generate the random expectation of mutual exclusivity, 100 pairs were randomly selected from the non-nGI groups of Union and from the conditional and non-nGI groups of Ito. Random expectations were generated by calculating the mutual exclusivity rates for the full combinatorial set of 100 gene pairs within each nGI group.

RNAi experiments
To experimentally validate the predicted active compensation between *RRAGC* and *RRAGD*, we performed siRNA-mediated knockdown experiments in an immortalized human astrocyte cell line. This cell line was generated by retroviral transduction of primary human astrocytes (ScienCell, Cat. No. 1801) with a pLXSN vector expressing the HPV16 E6/E7 oncogenes (Addgene, #52394), following the protocol described by Sonoda *et al.* [26]. Immortalized cells were transfected with one of three different siRNAs targeting *RRAGC* (synthesized by RiboBio) or a negative control siRNA using Lipofectamine RNAiMAX (Invitrogen). The siRNA sequences were as follows: siRNA1 (GTGACATGATCGATGTTGT), siRNA2 (TGATCGATGTTGTAATTGA), and siRNA3 (TGGCAATTATCAAGCTGAA). After 48 hours, total RNA was extracted using TRIzol reagent. RNA-seq libraries were prepared and sequenced. Raw reads were processed using a standard bioinformatics pipeline (Trimmomatic v0.39, STAR v2.7.11b, RSEM v1.3.3) [27-29], and differential expression of *RRAGD* was assessed using the Wald test implemented in the DESeq2 R package (v1.28.1) [30]. Please check “RNAi experiment” section of Supplementary Note for experimental details.

Entanglement analysis
To test the entanglement model of paralog retention, we adapted the methodology of Kuzmin *et al.* [31] from yeasts to vertebrates. In brief, for each human paralog pair, we retrieved orthologous coding sequences from eight phylogenetically representative vertebrate species (human, mouse, cattle, elephant, opossum, platypus, chicken, and lizard) from Ensembl. Multiple sequence alignments of the proteins were constructed using MAFFT (v7.505) [32] and then converted to codon alignments using PAL2NAL (v14) [33] with parameters of “-output paml -nogap”. We used the codeml program from the PAML package (v4.9j) [34] to estimate the site-specific ratio of non-synonymous to synonymous substitution rates (*K*_a_/*K*_s_ or ω) for each paralog. This analysis applied the site model M7, which assumes a null ω distribution following a beta distribution. The Spearman’s correlation of the codon-level ω values between the two paralogs was then calculated as a measure of their co-evolutionary constraint, or entanglement. The functional domain of *TLK1/TLK2* was annotated with InterPro [35].

Expression and sequence feature analyses
Duplication age, and duplication mechanism (WGD/SSD) were retrieved from GenTree database [36], and OHNOLOGS v2 database [37]. Pairs annotated in OHNOLOGS v2 and dated to the vertebrate common ancestor in GenTree were classified as WGD-derived; all others were classified as SSD-derived, with the age of the younger copy taken as the duplication age.

We calculated a suite of nine features for each paralog pair to explore the predictors of compensation. Protein identity was retrieved from Ensembl [6]. Expression level, expression asymmetry, and temporal expression specificity (τ index [38]) were calculated on the basis of the developmental transcriptome data [17]. Expression asymmetry was defined, for each developmental stage, as the median ratio of the expression level of the more highly expressed paralog to that of the less highly expressed paralog. The number of GO terms, GO semantic similarity, the number of shared PPI partners, and protein complex membership of paralogs were calculated as described in “Feature enrichment analyses across CRISPR screening data”. For each paralog pair, protein alignment fractions were calculated by reciprocal BLASTP (v2.2.26; default parameters) [39], with each paralog used once as the query and once as the target. For each direction, only the top hit was retained, defined by the lowest E-value or, in the case of a tie, the highest score. The pairwise alignment fraction was calculated as the mean of the query and target alignment fractions.

We compared expression and sequence features between core nGI and non-core pairs as follows. For the Union dataset, we used all pairs and ran two-sided Wilcoxon signed-rank tests. For the Ito dataset, we kept only the 50 gene families that contained both core and non-core negative GI pairs (see Table S12). Within each family, we averaged features for core and for non-core pairs, then compared these averages with a paired two-sided Wilcoxon signed-rank test.

A random forest model, implemented in the randomForest R package (v4.7.1.1) [40] with parameters mtry = 3 and ntree = 1000, was used to evaluate the relative importance of these features in predicting core nGI status. Feature importance was quantified using the mean decrease in Gini impurity (importance(..., type = 2)).

To formally test whether duplication age independently predicts core nGI status, we used the Ito dataset, chosen for its broader cell-line coverage and consistent screening of the same paralog pairs across all 11 cell lines. We fitted logistic regression models within the subset of paralog pairs with CDS identity <90%:

$$Model 1:logit\left( P\left( Y=1 \right) \right)=\beta_{0}+\beta_{1}Age+ \beta_{2}ExprBreadth+\beta_{3}ExprLevel+\beta_{4}SeqSim+\epsilon$$

$$Model 2:logit\left( P\left( Y=1 \right) \right)=\beta_{0}+\beta_{2}ExprBreadth+\beta_{3}ExprLevel+\beta_{4}SeqSim+\epsilon$$

Here, “Age” indicates the duplication age group; “ExprBreadth” indicates the fraction of co-expressed cell lines, calculated as the number of co-expressed cell lines divided by 11; “ExprLevel” indicates the median expression level across cell lines; and “SeqSim” indicates protein sequence identity.

Co-expressed and co-expressed/essential Ito subset

The co-expressed Ito subset comprised paralog pairs for which both genes were expressed at TPM ≥ 1 in all 11 screened cell lines. The co-expressed/essential subset further required both mouse orthologues to be classified as essential. Human–mouse orthology data were obtained from Ensembl [6], and essentiality annotations from International Mouse Phenotyping Consortium release 21.1 [41].

Cell-line-level specialization analysis

The classification of paralog-pair specialization and redundancy was adapted from a previous study [42]. We analyzed the Ito dataset because it provides broad cell-line coverage and assays the same paralog pairs across 11 cell lines. For each paralog pair in each cell line, pairs with both genes expressed, only one gene expressed or neither gene expressed were classified as Both_exp, Single_exp and Non_exp, respectively, using TPM ≥ 1 as the expression threshold. Dosage-specialized pairs were defined as pairs for which the expression spectrum of one paralog across cell lines was fully nested within that of the other. Dosage-redundant pairs were defined as pairs whose two paralogs shared the same expression spectrum across cell lines (Fig. S7a). nGI proportions were compared among Both_exp and Single_exp contexts separately for dosage-redundant and dosage-specialized pairs using one-sided Fisher’s exact tests. The Non_exp category was skipped because it showed minimal compensatory capacity (Fig. S2c).

Retrogene analyses
To analyze the compensatory potential of retrogenes, we first merged the Ito and Union datasets to increase statistical power. For pairs found in both datasets, we categorized them as follows: core negative GI if a pair was core in one dataset and core or conditional in the other; conditional negative GI if a pair was conditional in one dataset and conditional or non-nGI in the other; and non-nGI if a pair was conditional in one dataset and core or non-nGI in the other.

Retrogenes were identified by cross-referencing our paralog list with the RetrogeneDB [43] and GenTree [36] databases (Table S12). Paralog pairs were then categorized based on the chromosomal locations of the parental and daughter genes (*e.g.*, "X->A" for out-of-X retroposition events). We identified a total of 22 X-derived retrogene-associated pairs. The expression levels of these retrogenes and their parental genes were analyzed using bulk RNA-seq data from the Human Protein Atlas (HPA, v22.0) [44] and single-cell RNA-seq data from human spermatogenesis [45]. For single cell data, we retained only Ensembl-annotated genes from the normalized UMI matrix. For each cell type, we created pseudobulk profiles by: summing normalized counts across all cells of that type, scaling the total to 1e+06, and then transforming gene values as log2(x + 1).

The list of human orphan retrogenes and information on their parental genes in outgroup species were obtained from Ciomborowska *et al.* [46].

Supplementary Note

Design of sgRNA sequences

As shown in the Supplementary Note Fig. 1a, we ran in-house Python (version 3.4.8) scripts to design sgRNAs from the coding sequences (CDS) of each gene annotated by Ensembl version 73 [47], which corresponds to the UCSC hg19 reference genome [48]. For each gene, we used the CDS corresponding to the longest encoded protein. For each CDS, we predicted sgRNA sequences corresponding to the middle region (the 25% to 75% region) because frame-disrupting mutations in the middle region are more likely to disrupt the protein [49, 50].

We minimized the possibility of sgRNA off-targeting with the following considerations. Specifically, the editing activity of a sgRNA depends on the mismatches within the sgRNA pairing region. From the region close to the protospacer adjacent motif (PAM) to the distal region, mismatches become increasingly tolerable [51]. Although both mismatch number and mismatch positions have impacts on the editing activity of off-target sites, more than 3 mismatches seldom lead to efficient editing at an off-target site [52]. With this knowledge, we mapped the 20 nt spacer sequences plus an 3’ NGG PAM to the reference genome (hg19) with Bowtie version 1.2.2 [53]. Perfect mapping positions were directly identified while potential off-target sites were identified in the following way: 1) mismatches within 20 nt spacer regions were divided into two parts based on their position, *i.e.*, the 12 nt PAM distal region and the 8 nt PAM proximal region; and 2) a potential off-target was identified when there were at most 1 mismatch in the proximal region and at most 3 mismatches in the full 20 nt protospacer region. All potential off-target sites were then intersected with all annotated CDS in the genome. We retained 853,459 sgRNAs that exhibited perfect single-gene targeting and were free of any off-target on other coding genes. That is, potential off-targets on other CDS were not allowed, whereas potential off-targets on noncoding regions were permissible. We used this hybrid strategy to maximize the sgRNA coverage of duplicate gene pairs by ensuring sgRNA specificity for individual duplicate genes without overtly restricting the sample size of usable sgRNAs.

For these retained sgRNAs, we used two well-developed models (Doench-Root score and SSC score [54, 55]) to predict their editing efficiency based on sequence contexts. We retained 288,549 sgRNAs with both scores exceeding the corresponding median values of all sgRNAs. For sgRNAs targeting the same gene, we ranked the sgRNAs first by the absence of non-CDS off-target sites and then by the sum of the two scores. For one gene, we chose the top four sgRNAs and excluded genes having fewer than four targeting sgRNAs. By this filter, we obtained 62,444 sgRNAs targeting 15,611 coding genes.

We then specified two sets of control sgRNAs. For the first set, given intergenic regions of the UCSC human genome assembly (hg19), we predicted 414,365 targeting sgRNAs, which are located at least 20 kb from any protein-coding genes (Supplementary Note Fig. 1b). We then mapped these sgRNAs back to the human genome and obtained 1,497 intergenic targeting sgRNAs with one perfect match and free of any off-target site. For the second set, given chromosome 2R of the UCSC *D. melanogaster* genome assembly (dm6), we extracted 375,892 unique 20-nucleotide sequences. After mapping these sequences to the human genome, we identified 4,165 non-targeting sgRNAs with no detectable on-target or off-target matches (Supplementary Note Fig. 1c).

Specification of sgRNA pairs

To target one gene, we designed four different sgRNA pairs following the conventional “sgRNA (gene targeting)-Control (non-targeting sgRNA)” [14, 56] format, in which the former sgRNA is driven by the human U6 promoter and the latter is driven by the monkey U6 promoter..

To target one gene pair (*e.g.* Gene1 and Gene2), although 32 possible sgRNA combinations exist (4 sgRNAs per gene x 2 position options), we only chose four combinations due to library size constraints. Based on four ranked sgRNAs designed for either gene 1 or gene 2, we made four sequential combinations to target a gene pair: Gene1_sgRNA1-Gene2_sgRNA1, Gene1_sgRNA2-Gene2_sgRNA2, Gene1_sgRNA3-Gene2_sgRNA3 and Gene1_sgRNA4-Gene2_sgRNA4.

We further designed three internal control sets: 1) to target one essential gene with the design “sgRNA (gene targeting)-Control”; 2) to target intergenic regions as “sgRNA (intergenic targeting)-Control”; and 3) non-targeting controls as “Control-Control”.

Notably, all non-targeting control sgRNAs described above were randomly sampled from the aforementioned 4,165 non-targeting sgRNAs.

Overall structure of the screening library and sampling of 687 duplicate pairs

The library includes 500 non-targeting sgRNA pairs constructed as “Control-Control”, 500 intergenic targeting sgRNA pairs constructed as “sgRNA-Control”, 812 essential gene targeting sgRNA pairs constructed as “sgRNA-Control”, 3,096 dual gene targeting sgRNA pairs constructed as “sgRNA-sgRNA”, and 5,632 single gene targeting sgRNA pairs constructed as “sgRNA-Control”. We additionally randomly chose 1,074 “sgRNA-Control” single gene targeting constructs and reversed the sgRNA position as “Control-sgRNA” constructs. Therefore, all six groups summed to 11,614 constructs.

These groups served different purposes. Specifically, non-targeting controls were used to estimate the baseline fitness loss. The essential genes targeting sgRNA pairs were used to validate the efficiency of sgRNAs. Herein, 203 essential genes were randomly chosen from the published essential gene list for HCT116 [57]. Note that these genes are similarly essential in GM12878. Dual targeting sgRNA pairs and single gene targeting sgRNA pairs were used to infer the GI score of the double knockout. Among a total of 774 gene pairs, 14 pairs belonged to the positive control Set1 (Table S3), and these included interactions identified in four small-scale studies [58-61]. Another 73 pairs belonged to the positive control Set2, which showed the top negative GI score in one large-scale screening conducted in the K562 cell line [60]. Pairs in Set1 are expected to exhibit negative genetic interactions more frequently than those in Set2. The remaining 687 gene pairs were randomly selected duplicate pairs, which were also identified as nonessential in published HCT116 screening data [57]. Specifically, these pairs were randomly sampled in two ways: 1) 179 WGD duplicate (ohnolog) pairs identified by [62], with age confirmed by Gentree database; 2) 499 SSD duplicate pairs annotated in the GenTree database [36], including information on age and duplication mechanism.

Cell culture, lentivirus production and sgRNA plasmid cloning

HEK293T cells were cultured in Dulbecco’s modified Eagle’s medium (DMEM) (Corning) supplemented with 10% fetal calf serum (Gibco) and 1% penicillin/streptomycin (Gibco). HCT116-Cas9 cells were maintained in Iscove’s modified Dulbecco’s medium (IMDM) (Gibco) supplemented with 10% fetal calf serum (Gibco) and 1% penicillin/streptomycin (Gibco). GM12878-Cas9 cells were cultured in RPMI-1640 (Corning) with 10% fetal calf serum (Gibco) and 1% penicillin/streptomycin (Gibco). The HCT116-Cas9 cell line was purchased from EdiGene, and the GM12878-Cas9 cell line was kindly provided by Dr. Wensheng Wei’s laboratory at Peking University. The cells were cultured in an incubator at 37 °C with 5.0% CO_2_ and passaged once every 2-3 days.

HEK293T cells (7×10^6^) were plated in 6-well plates in 2 mL of DMEM with 10% FBS. After 24 h, the cells were transfected according to the manufacturer’s protocol. A DNA mixture containing the transfer vector (2.1 μg), the packaging plasmids psPAX2 (Addgene #12260, 1.5 μg) and pMD2.G (Addgene #12259, 0.7 μg) and Neofect^TM^ transfection reagent (Neo Biotech, 4.2 μL) was added to Opti-MEM (Corning, 210 μL). The mixture was incubated at room temperature for 25 min and then gently added to the cell medium. The culture medium was replaced by viral production medium (Lonza) 16 h after transfection. The supernatant containing lentivirus was collected 36 h after this media change, filtered through a 0.45-μm PVDF filter membrane (Millex-HV) and stored at -80 °C. To generate the paired gRNA library, lentivirus production was scaled up using the same experimental procedures.

The 110-nt paired gRNA oligonucleotides were synthesized by GenScript. The sequences were designed as follows:

GTGGAAAGGACGAAACACCGNNNNNNNNNNNNNNNNNNNNGTTTA**GAGACG***NNNNNNNN***CGTCTC**CACCGNNNNNNNNNNNNNNNNNNNNGTTTTAGAGCTAGAAATAGC

N: 20-nt gRNA spacer

*N*: 8-nt random sequences

**Bold**: BsmBI recognition site

Paired gRNA oligonucleotides were amplified by primers targeting the flanking regions of oligonucleotides that were complementary to the gRNA-expressing vector lentiGuide-Puro (Addgene #52963) or lentiGuide-GFP. The lentiGuide-GFP latter vector was constructed based on lentiGuide-Puro by changing the puromycin resistance flag to GFP. The amplicons were cloned into the BsmBI digested vector using the Gibson Assembly method and transformed into trans5α chemically competent cells (Transgen Biotech). The gRNA scaffold on the left side and monkey (rhesus macaque) U6 promoters were inserted into BsmBI-digested paired gRNA plasmids. The mutagenized gRNA scaffold sequences for the left gRNA were described by [63], which showed increased activity and reduced recombination. The monkey U6 promoter was provided by Cyagen. The sequence of the insert was the following:

GTTTCAGAGCTATGCTGGAAACTGCATAGCAAGTTGAAATAAGGCTAGTCCGTTATCAACTTGAAAAAGTGGCACCGAGTCGGTGCTTTTTTGGCCGGCC**GAGGGCCTATTTCCCATGAGTCCTTCATATTTGCATATACGATGCAAGAATGTTAGAGAGATAATTAGAATTAATTTGGCTATAAACATAAAGATATTAGTACAAAATATTGATGCAGAAAGTAATAATTTCTTGGGTAGTTTGTAATTTTAAAATTATGTTTTAAAATGGACCATGACATACTTGCCGTAAGTGTAAAGTATTTCTATTTCTTGCCTTTATATATCTTCTGGCAAGTTACCAACACCG**

Underline: left gRNA scaffold

**Bold**: monkey U6 promoter

The ligation mixture was transformed into trans5α chemically competent cells to obtain the final paired gRNA plasmids.

Library construction, CRISPR screening and sequencing

The paired gRNA library plasmids were constructed by Cyagen. The library was delivered into 400 million HCT116-Cas9 cells or GM12878-Cas9 cells by lentiviral infection at a multiplicity of infection (MOI) of ~0.3 and a library coverage of 1,000 cells per paired gRNA. A relatively smaller MOI (fewer viruses compared with cells) ensures that each cell is transfected by one or zero viruses. Two biological replicates were generated. The infected cells were sorted by flow cytometry (Sony SH800S instrument) for green fluorescence 2 days post infection and allowed to recover for ~48 h. At this moment, 12 million cells were collected as reference samples (T0). The remaining cells were passaged once every 3 days while keeping the library coverage of 1,000 cells per paired gRNA. The total culture time was 21 days (T21) for HCT116-Cas9 cells and 15 days (T15) for GM12878-Cas9 cells because we observed that GM12878 cells grew faster than HCT116 cells. These time lengths were also routinely used in CRISPR screening [54, 64]. Pools of cell populations at different time points (T9, T18 and T21 for HCT116 and T9 and T15 for GM12878) were harvested during culture for genomic DNA extraction and sequencing. Notably, for the HCT116 T21 and GM12878 T15 cells, we obtained and sequenced three separate technical replicates of each biological replicate.

Genomic DNA was extracted from each sample using the Blood & Cell Culture DNA Maxi Kit (QIAGEN). Regions corresponding to paired gRNAs were amplified by PCR using NEBNext High-Fidelity 2x PCR Master Mix (NEB). For each sample, in the first round of PCR, 1 μg of genomic DNA was used as the template for each reaction, and 25 reactions were performed. In the second round of PCR, 2 μL of the PCR products from the first round were amplified for each reaction, and 10 reactions were performed. The specific information, including primers and experimental conditions, is as follows:

PCR_1st_forward,

TACACGACGCTCTTCCGATCTCTTGTGGAAAGGACGAAACA;

PCR_1st_reverse,

GTGACTGGAGTTCAGACGTGTGCTCTTCCGATCTCCTTATTTTAACTTGCTATTTCTAGCTCTA;

PCR_2nd_forward,

AATGATACGGCGACCACCGAGATCTACACTCTTTCCCTACACGACGCTCTTCCGATC; and

PCR_2nd_reverse,

CAAGCAGAAGACGGCATACGAGATNNNNNNGTGACTGGAGTTCAGACGTG (N: Illumina sequencing index).

The thermocycling conditions of the first round of PCR were as follows: initial denaturation of 30 s at 98 °C; 18 cycles of 10 s at 98 °C, 30 s at 60 °C, and 30 s at 72 °C; and a final extension of 5 min at 72 °C.

The thermocycling conditions of the second round of PCR were as follows: an initial denaturation of 30 s at 98 °C; 10 cycles of 10 s at 98 °C, 30 s at 60 °C, and 30 s at 72 °C; and a final extension of 5 min at 72 °C.

The final PCR products were purified with a Zymoclean Gel DNA Recovery kit (Zymo) and sequenced on a HiSeq X10 platform (Illumina).

Quantification of GIs based on dependency scores

As conventionally performed (*e.g.*, [65]), we calculated the dependency score as the relative fold change of reads mapping to a sgRNA and interpreted this score as the loss of fitness with the corresponding gene knocked out. According to previous RNAi-based screens [66], we quantified the interaction strength as follows.

First, because the cell population exhibits exponential growth during the passaging process, the wild-type (WT) cell population dynamics can be described as follows:

$C_{t}^{WT}=C_{0}^{WT}*2^{kt}$, (2)

where

$C_{t}^{WT}$: wild-type cell population size at day 0,

$C_{t}^{WT}$: wild-type cell population size at day t,

$k$: doubling factor of the exponential growth, and

$t$: day t.

For a specific cell population with decreased fitness, such as a cell population targeted by one sgRNA, the population dynamics can be described as

$C_{t}^{'}=C_{0}^{'}*2^{k(1-s)t}$, (3)

where

$C_{t}^{'}:$ cell population size at day 0,

$C_{0}^{'}:$ cell population size at day t, and

$s:$ fitness loss of a cell population compared with the WT cell population.

After transformation, we could obtain *s* as

$s=[{log}_{2} (\frac{C_{t}^{WT}}{C_{0}^{WT}})-{log}_{2} (\frac{C_{t}^{'}}{C_{0}^{'}})]/kt$. (4)

We could not obtain the exact cell population size and thus used the read depth to represent the relative size of a cell population. The depth is proportional to the population size; thus,

$$C_{0}=N_{0}*R_{0}$$

$C_{t}=N_{t}*R_{t}$, (5)

where

$N_{0}:$ read depth of a cell population at day 0,

$N_{t}$: read depth of a cell population at day t,

$R_{0}:$ relative ratio of the read depth to the population size at day 0, and

$R_{t}:$ relative ratio of the read depth to the population size at day t.

Because $R_{0}$and $R_{t}$ are constants, the fitness loss can be calculated as

$s=[{log}_{2} (\frac{N_{t}^{\text{WT}}}{N_{0}^{WT}})-{log}_{2} (\frac{N_{t}^{'}}{N_{0}^{'}})]*\frac{1}{kt}$. (6)

For a specific experiment, $kt$ is a constant. We do not need to derive the exact value of the constant $kt$ because it has no effect on whether two genes exhibit GIs. Therefore, we only used the read depth to calculate the empirical (*emp*) fitness loss:

$s^{emp}={log}_{2} (\frac{N_{t}^{\text{WT}}}{N_{0}^{WT}})-{log}_{2} (\frac{N_{t}^{'}}{N_{0}^{'}})$. (7)

For two genes A and B, when we have read depth data from single-gene knockout and dual-gene knockout from day 0 and day t, we can obtain

$s_{A}^{emp}=[{log}_{2} (\frac{N_{t}^{WT}}{N_{0}^{WT}})-{log}_{2} (\frac{N_{t}^{a}}{N_{0}^{a}})]$

$s_{B}^{emp}=[{log}_{2} (\frac{N_{t}^{WT}}{N_{0}^{WT}})-{log}_{2} (\frac{N^{b}}{N_{0}^{b}})]$

$s_{AB}^{emp}=[{log}_{2} (\frac{N_{t}^{WT}}{N_{0}^{WT}})-{log}_{2} (\frac{N_{t}^{\text{ab}}}{N_{0}^{\text{ab}}})]$. (8)

Under the basic additive model of epistasis, the GI score would be

${GI_{AB}}^{emp}=\left( s_{A}^{emp}+s_{B}^{emp} \right)-s_{AB}^{emp}$. (9)

In studies of CRISPR-based screening, a more generally used term is the dependency score ($d$) instead of fitness loss. These variables follow a linear relationship:

$d^{emp}=-s^{emp}={log}_{2} (\frac{N_{t}^{'}}{N_{0}^{'}})-{log}_{2} (\frac{N_{t}^{\text{WT}}}{N_{0}^{WT}})$. (10)

Therefore,

${GI_{AB}}^{emp}=d_{AB}^{emp}-\left( d_{A}^{emp}+d_{B}^{emp} \right)$. (11)

Analysis of screening data using the inverted variance weighting method

We calculated dependency scores according to the read mapping data. Because an sgRNA pair consists of two reads, we mapped them separately to the plasmid sequences using Bwa version 0.7.17-r1188 [67] and calculated the depth. We implemented two filters to exclude sgRNAs with low-quality mapping. Recombination rate of our method is around 34%, which is in reasonable range of dual knockout system [1, 60]. We first removed combinations of two sgRNAs that did not belong to the library and should thus represent recombination products [68]. We further excluded sgRNA pairs with a low initial abundance. Specifically, for HCT116 and GM12878 cells, we used distinct strategies to filter the raw data based on the read counts at day 0. For the former cell line, we retained the sgRNA pairs with the top 80% raw read counts at day 0. For the latter, the cutoff was modified to 75% to retain more data because this cell line exhibited more random fluctuation. We also excluded sgRNA pairs with only zero or one read at any passaging days (> day 0) since data with small read count is less reliable. For HCT116 and GM12878, dependency scores from three technical replicates at the final time point were averaged using the mean.

We extended [69] and modeled the variance of all sgRNA constructs in one library. Specifically, we had two biological replicates of each cell line. For each replicate, we fitted the relationship of the T0 read counts and the standard deviation (SD) of the dependency scores for all non-targeting sgRNA pairs with an exponential family linear function. We then used this function to model the variance of all sgRNA constructs in the same library. To avoid a negative value for the estimated variance, the fitted variance (SD_low_) corresponding to the median T0 read count of the fifteenth non-targeting strata (the strata with highest T0 read count, Supplementary Note Fig. 4a) was set as the lower bound, and any fitted variance lower than SD_low_ was reset as this value. The rationale is that exponential decay could lead to a negative SD, as shown in the right tail of Supplementary Note Fig. 4c; thus, we set the SD with an even more abundant read count than the median fifteenth non-targeting strata as SD_low_.

For all sgRNA pairs targeting the same gene or the same gene pair, we obtained gene or gene pair-level statistics using the inverted variance weighting methodology. The formula is the following:

$$d\left( gene j \right)=\frac{\sum\left( \frac{1}{var(i)}*d(i) \right)}{\sum\left( \frac{1}{var(i)} \right)}$$

$var\left( gene j \right)=\frac{1}{\sum\left( \frac{1}{var(i)} \right)}$ , (12)

where

$i:$ sgRNA pair targeting gene$j$,

$d\left( i \right):$ dependency score corresponding to sgRNA pair $i$, and

$var\left( i \right):$ empirical variance of the score corresponding to sgRNA pair $i$.

Between two biological replicates, we assessed whether the gene pair-level dependency score was sufficiently consistent. The difference in the estimated dependency score between two replicates should follow a normal distribution:

$({d(gene j)}^{rep1}-{d\left( gene j \right)}^{rep2})\sim N(0, {var}^{replicates})$ , (13)

where

${var}^{replicates}={var\left( gene j \right)}^{rep1}+{var\left( gene j \right)}^{rep2}$. (14)

At the 95% confidence interval, the difference should be within a range,

$absolute\left( {d(gene j)}^{rep1}-{d\left( gene j \right)}^{rep2} \right)<1.96*{sd}^{replicates}$. (15)

If so, the two biological replicates were considered to show good concordance. We then obtained an estimation of the dependency score and its variance based on inverted variance weighting:

$${d\left( gene j \right)}^{final}=\frac{\sum\left( \frac{1}{{var\left( gene j \right)}^{rep k}}*{d(gene j)}^{rep k} \right)}{\sum\left( \frac{1}{{var\left( gene j \right)}^{rep k}} \right)}$$

${var\left( gene j \right)}^{final}=\frac{1}{\sum\left( \frac{1}{{var\left( gene j \right)}^{rep k}} \right)}$	, (16)

where

$k:$ biological replicate ID, *i.e.*, 1 or 2.

If the above is not true, a similar formula was used (the variance was specified as the larger value from variance estimates among replicates)

$${d\left( gene j \right)}^{final}=\frac{\sum\left( \frac{1}{{var\left( gene j \right)}^{rep k}}*{d(gene j)}^{rep k} \right)}{\sum\left( \frac{1}{{var\left( gene j \right)}^{rep k}} \right)}$$

${var\left( gene j \right)}^{final}=maximum({var\left( gene j \right)}^{rep1},{var\left( gene j \right)}^{rep2})$. (17)

The estimated dependency score and estimated variances for each gene and each gene pair were obtained, which enabled the identification of significant negative GI relationships.

According to equation (11) described above,

${GI_{AB}}^{final}=d_{AB}^{final}-\left( d_{A}^{final}+d_{B}^{final} \right)$. (18)

As a null hypothesis, the GI score should follow a normal distribution,

${GI_{AB}}^{final}\sim N(0, {var}^{GI})$. (19)

Herein,

${var}^{GI}={{var\left( gene pair AB \right)}^{final}+ var\left( gene A \right)}^{final}+{var\left( gene B \right)}^{final}$.

(20)

Therefore, we calculated the significance of the deviation from zero for the GI score of each gene pair. To increase the sensitivity, we performed a one-sided test of significance at the false discovery rate (FDR)-adjusted *q* cutoff, *i.e.*, 0.05.

Because of the relatively small amount of sgRNAs targeting each gene, we chose not to use non-parametric method to analyze our data. Moreover, our variance weighted method treats each sgRNAs differently according to their different initial abundance, which is more suitable for our data. Note that we additionally performed DESeq2 (version 1.28.1)-based variance modeling [70]. Our variance-weighted method performed slightly better than, or at least comparably to, the conventional mean and DESeq2 approaches (Supplementary Note Fig. 4d).

Estimation of dual editing efficiency

The dual editing efficiency was estimated via single-cell cloning method and was assessed as follows. Paired gRNA plasmids were packaged into lentivirus and transduced into HCT116-Cas9 cells, and cells were selected with 1.5 μg/mL puromycin for 2 days. The selected cells were plated in 96-well plates as single cells and cultured for 12 days. Genomic DNA was extracted from each single-cell-derived clone. The targeted sequences were amplified by PCR. The PCR products were purified by DNA Clean & ConcentratorTM-5 (ZYMO) and cloned into a TOPO vector using the pCloneEZ-Omni-Amp/HC Cloning Kit (Clone Smarter) according to the manufacturer’s instructions. Mutations were identified by manual inspection after Sanger sequencing.

Experimental validations of negative GI status for 17 gene pairs

We performed a cell viability assay to validate 17 negative GI gene pairs. In brief, paired gRNA plasmids targeting two genes and single gRNA plasmids targeting one gene and one control site (non-targeting region, NT) were packaged into lentiviruses and transduced into HCT116-Cas9 cells (Table S2). Cells were sorted by flow cytometry (Sony SH800S) for green fluorescence 2 days post infection and allowed to recover for ~24 hours. For each gRNA, one thousand cells were seeded in an opaque-walled 96-well plate. The relative proliferation rates of the cells were measured for 5 days by CellTiter-Glo (Promega) assay or CCK8 (Dojindo) assay according to the manufacturer’s instructions. The absorbance at 450 nm or luminescence was measured with a microplate reader (Biotek Synergy H1).

The pairs included three duplicate pairs with negative GIs in our screening and all 14 Set1 pairs in which six showed negative GIs in our screening. We monitored the cell growth dynamics and fitted the dynamics with exponential family curves with *R* language (version 3.6.3):

$N_{t}=N_{0}*\exp\left( k*t \right)$, (21)

where

*t*: days,

*N_t_*: cell number at days *t*, and

*k*: growth rate.

The growth rate relative to non-targeting control sgRNA was defined as the fitness loss(*s*) of an sgRNA construct:

$s_{sgRNA}=\frac{k_{sgRNA}-k_{NT}}{k_{NT}}$. (22)

According to the additive model, the expected fitness loss of digenic knockout is calculated as the sum of fitness loss for each single gene knockout. We performed a two-sided Student’s t test to examine whether the observed fitness loss of digenic knockout was significantly lower than the expected value under the additive model. A *P-*value of 0.05 was used as the cutoff to define a significant negative GI relationship.

As shown in (Table S2), four sgRNA pairs were designed for each gene pair. Among the four pairs, we randomly chose one sgRNA pair with a negative dependency score to validate their negative GI in the HCT116 cell line. For genes such as *AKT1*, all four sgRNAs have positive dependency scores, and thus, we randomly selected one. We first performed a pilot study of sgRNA efficiency in which we compared the cell growth after transduction with double knockout constructs against that of wild-type cells. Among 17 gene pairs, we found that cell growth did not change for four pairs: "*SMARCA2/SMARCA4*", "*ARID1A/ARID1B*", "*CDH1/CDH3*" and "*HSP90AA1/IMPDH1*". Therefore, we directly assessed their editing efficiencies. Different from the aforementioned time-consuming single-cell cloning experiments, we herein performed a TIDE assay, which generated a gross estimation of the efficiency by analyzing the Sanger sequencing data around the target site [71]. We found that sgRNA pairs targeting "*SMARCA2/SMARCA4*" and "*ARID1A/ARID1B*" had low editing efficiencies (< 50%). Therefore, in the final validation experiments, the originally designed sgRNA pairs for "*SMARCA2*/*SMARCA4*" and "*ARID1A/ARID1B*" were replaced by randomly sampling one of the three remaining sgRNAs.

For relatively time-consuming cell titer-based growth monitoring, we only examined three duplicate pairs, including *NABP2/NABP1*, *NDE1/NDEL1*, and *SRSF4/SRSF6*, and confirmed that all these pairs exhibited negative epistasis (Supplementary Note Fig. 2).

We additionally performed CCK8-based cell growth monitoring for all 17 gene pairs. Four of the six Set1 pairs called with negative GIs were successfully validated. Two pairs, including *ARID1A/ARID1B* and *CDH1/CDH3*, were rejected and should represent false positives in our screening data (Supplementary Note Fig. 3a). Among the remaining eight Set1 pairs without negative GIs in the screening, three (*ALAD/GPI*, *CDK4/CDK6* and *BRCA1/PARP1*) showed negative GIs in the validations (Supplementary Note Fig. 3b). Therefore, the sensitivity and specificity of the screening were estimated as 70% (7/10) and 71% (5/7), respectively.

Quality control of data generation and analysis algorithm

First, low editing efficiency of sgRNAs would impair the power of our data to resolve epistatic relationships. With single-cell cloning analysis, we estimated a dual editing efficiency between 67% (10/15) and 86% (18/21) with 50% (5/10) and 50% (9/18) editing outcomes being loss-of-function mutations (frame-shift mutations or large deletions) for both genes (Fig. S1a; Table S2). These results demonstrate that our system achieves comparable efficiency in generating stable dual knockouts to those reported in previous studies [4, 14, 72].

Second, because the random fluctuation of cell passaging affects the screening power, particularly for cells with an initial low abundance (reflected by the small read count in T0 [73]), we developed a method to control this effect (variance-weighted method). Taking the first replicate of HCT116 as an example, the read count of non-targeting sgRNAs (negative controls) spans 15 strata, with counts in the lowest stratum of approximately 100 and counts in the highest stratum greater than 10,000 (Supplementary Note Fig. 4c, Table S2). Due to the fluctuation of passaging [73], the dependency scores in the lower strata deviated more from the expected 0 (no fitness effect). The SDs of the scores for these control sgRNAs fit an exponential decay formula (Supplementary Note Fig. 4a). Presumably, the other sgRNAs also suffer from this effect, and we thus calculated their SDs given the formula. Using the inverse of variances as weights, we calculated the weighted mean of sgRNA level and gene level scores. Our variance-weighted method performed slightly better than, or at least comparably to, conventional approaches [74, 75], showing higher between-replicate correlations (0.87 *vs.* 0.80-0.85 for HCT116 and 0.80 *vs.* 0.71-0.75 for GM12878; Supplementary Note Fig. 4b, d; Fig. 1a).

In addition to the high between-replicate correlation, we followed previous efforts (*e.g.*, [4]) and performed another four evaluations at both the gene and pair levels (Fig. S1a): 1) essential genes showed expected higher dependency scores relative to duplicate genes in both cell lines (Supplementary Note Fig. 4e); 2) 6/14 (0.43) and 5/72 (0.07) of positive pairs (Set1 and Set2) have been rediscovered by HCT116 or GM12878 pooled screening, with the experimentally verified Set1 being more reliable (Table S2); 3) For 1,074 pairs evaluated, the sgRNA level score was largely reproducible with promoter position switched in both cell lines (correlation: 0.47-0.56, Supplementary Note Fig. 4f); 4) as aforementioned, validation experiments of 17 pairs indicated a sensitivity of 0.70 and a specificity of 0.71 (Table S2). These evaluations showed that the quality of our screening was comparable (Fig. S1a) to that of the previous studies [4, 13-15, 72, 76].

In summary, we built a Cas9-based screen targeting 687 paralog pairs (678 retained after applying T0 abundance and other filters) and identified 42 negative GI pairs in HCT116 or GM12878 cells.

The RNAi experiment design

Three siRNAs were designed to knock down *RRAGC* expression in an immortalized human astrocyte-derived cell line, with each siRNA paired with a control group. For each siRNA and control group, three biological replicates were performed.

- **Plasmids**

Given the limited proliferative capacity (approximately 7-8 passages) of normal human primary astrocytes, we adopted a retroviral transduction strategy to achieve immortalization, following the protocol described by Sonoda *et al* [26]. Briefly, a retrovirus expressing the HPV16 E6/E7 oncogenes was employed to infect human primary astrocytes. The pLXSN16E6E7 plasmid (Addgene, #52394) was obtained from Addgene.

- **siRNA stock**

siRNA-mediated silencing of the target gene *RRAGC* was performed using the sequences: siRNA1 (GTGACATGATCGATGTTGT), siRNA2 (TGATCGATGTTGTAATTGA), and siRNA3 (TGGCAATTATCAAGCTGAA). Lyophilized siRNA vials were briefly centrifuged, and 250 µL of nuclease-free water was added to prepare 20 µM siRNA stock solutions.

- **Cell Culture**

Human Astrocytes (HA) were purchased from Shanghai Zhong Qiao Xin Zhou Biotechnology Co., Ltd. Cells were cultured in Astrocyte Medium (ScienCell, Cat. No. 1801). This medium was supplemented with 2% fetal bovine serum (FBS; ScienCell, Cat.No.0010), 1% Penicillin-Streptomycin (P/S, ScienCell, Cat. No. 0503), and 1% Astrocyte Growth Supplement (AGS, ScienCell, Cat. No. 1852). Cultures were maintained in a humidified incubator at 37 °C with 5% CO₂ and passaged every 3-4 days to ensure logarithmic growth. All cell lines were routinely tested for mycoplasma contamination, with consistently negative results.

- **Construction of Immortalized Astrocytes**

HEK293T cells were seeded into 100-mm culture dishes coated with poly-L-lysine (Sigma, P4832) and cultured to ~90% confluency. For each 100-mm dish, HEK293T cells were co-transfected with 10.5 μg pLXSN16E6E7 plasmid, 7.5 μg psPAX2 (Addgene, #12260), and 3 μg pMD2.G (Addgene, #12259) using 42 μL of Neofect DNA Transfection Reagent (Neo Biotech). After 16 hours of transfection, the medium was replaced with fresh medium. Viral supernatants were collected at 48 hours and 72 hours post-transfection, filtered, and stored at -80°C.

To optimize infection, wild-type human primary astrocytes were seeded on day 0 at an appropriate density and exposed to varying volumes of viral supernatant supplemented with polybrene (final concentration: 8 µg/mL). At 24 hours post-infection (day 2), the medium was replaced with fresh medium containing G418, and cells were cultured until day 3. Following antibiotic selection, the viral volume resulting in approximately 50% cell survival was selected for subsequent experiments. The minimal effective volume of viral supernatant, determined by retroviral titer, was used to transduce wild-type human primary astrocytes. The medium was replaced 24 hours post-transduction, and cells were passaged at 48-hour post-transduction. When the adherent cells reached approximately 30-40% confluence, G418 selection was initiated and maintained until complete elimination of the uninfected control cells. Subsequently, cells were recovered in fresh medium for 24 hours, followed by single-cell clone isolation. Clones identified as immortalized astrocytes were confirmed by Western blot analysis showing significantly reduced expression of p53 and Rb proteins, while maintaining GFAP expression levels comparable to the parental (non-immortalized) cells and retaining the characteristic morphology of primary astrocytes. Twenty-four hours prior to the *RRAGC* knockdown experiment, wild-type immortalized human astrocyte-derived cells were seeded onto poly-L-lysine (Sigma, Cat. No. P4832)-coated 6-well plates at a density of 1.5 × 10⁵ cells per well. When cells reached 30%-50% confluency, they were transfected with either negative control siRNA (NC siRNA) or RRAGC siRNAs (siRNA1, siRNA2, and siRNA3). All siRNAs were synthesized by Guangzhou RiboBio Co., Ltd. For each well of a 6-well plate, 5 μL of 20 μM siRNA stock solution was co-transfected with 5 μL of Lipofectamine RNAiMAX Reagent (Invitrogen, Cat. No. 13778-075). Sixteen hours post-transfection, the medium was replaced with fresh medium. Forty-eight hours after transfection, cells were harvested, lysed in 1 mL of TRIzol reagent, and stored at -80 °C for RNA-seq analysis. RNA-sequencing was performed at GenePlus company with the standard RNA-seq protocol.

- **Data analysis**

Raw reads were processed using a standard bioinformatics pipeline (Trimmomatic v0.39, STAR v2.7.11b, RSEM v1.3.3) [27-29], and differential expression of *RRAGD* was assessed using the Wald test implemented in the DESeq2 R package (v1.28.1) [30].

**Supplementary References**

1.	Horlbeck MA, Xu A, Wang M *et al.* Mapping the Genetic Landscape of Human Cells. *Cell*. 2018; **174**: 953-967 e922. doi: 10.1016/j.cell.2018.06.010

2.	Billmann M, Costanzo M, Zhang X *et al.* A global genetic interaction map of a human cell reveals conserved principles of genetic networks. *bioRxiv*. 2025. doi: 10.1101/2025.06.30.662193

3.	De Kegel B, Ryan CJ. Paralog buffering contributes to the variable essentiality of genes in cancer cell lines. *PLoS Genetics*. 2019; **15**: e1008466. doi: 10.1371/journal.pgen.1008466

4.	Dede M, McLaughlin M, Kim E *et al.* Multiplex enCas12a screens detect functional buffering among paralogs otherwise masked in monogenic Cas9 knockout screens. *Genome Biology*. 2020; **21**: 262. doi: 10.1186/s13059-020-02173-2

5.	Giurgiu M, Reinhard J, Brauner B *et al.* CORUM: the comprehensive resource of mammalian protein complexes-2019. *Nucleic Acids Research*. 2019; **47**: D559-D563. doi: 10.1093/nar/gky973

6.	Cunningham F, Achuthan P, Akanni W *et al.* Ensembl 2019. *Nucleic Acids Research*. 2019; **47**: D745-D751. doi: 10.1093/nar/gky1113

7.	Thomas PD, Ebert D, Muruganujan A *et al.* PANTHER: Making genome-scale phylogenetics accessible to all. *Protein Science*. 2022; **31**: 8-22. doi: 10.1002/pro.4218

8.	Huttlin EL, Bruckner RJ, Navarrete-Perea J *et al.* Dual proteome-scale networks reveal cell-specific remodeling of the human interactome. *Cell*. 2021; **184**: 3022-3040.e3028. doi: 10.1016/j.cell.2021.04.011

9.	Yu GC, Li F, Qin YD *et al.* GOSemSim: an R package for measuring semantic similarity among GO terms and gene products. *Bioinformatics*. 2010; **26**: 976-978. doi: 10.1093/bioinformatics/btq064

10.	Paradis E, Schliep K. ape 5.0: an environment for modern phylogenetics and evolutionary analyses in R. *Bioinformatics*. 2019; **35**: 526-528. doi: 10.1093/bioinformatics/bty633

11.	Yu GC, Smith DK, Zhu HC *et al.* GGTREE: an R package for visualization and annotation of phylogenetic trees with their covariates and other associated data. *Methods in Ecology and Evolution*. 2017; **8**: 28-36. doi: 10.1111/2041-210x.12628

12.	Xu SB, Dai ZH, Guo PF *et al.* ggtreeExtra: Compact Visualization of Richly Annotated Phylogenetic Data. *Molecular Biology and Evolution*. 2021; **38**: 4039-4042. doi: 10.1093/molbev/msab166

13.	Parrish PC, Thomas JD, Gabel AM *et al.* Discovery of synthetic lethal and tumor suppressor paralog pairs in the human genome. *Cell Reports*. 2021; **36**: 109597. doi: 10.1016/j.celrep.2021.109597

14.	Thompson NA, Ranzani M, van der Weyden L *et al.* Combinatorial CRISPR screen identifies fitness effects of gene paralogues. *Nature Communications*. 2021; **12**: 1302. doi: 10.1038/s41467-021-21478-9

15.	Ito T, Young MJ, Li R *et al.* Paralog knockout profiling identifies DUSP4 and DUSP6 as a digenic dependence in MAPK pathway-driven cancers. *Nature Genetics*. 2021; **53**: 1664-1672. doi: 10.1038/s41588-021-00967-z

16.	Zhou YY, Zhou B, Pache L *et al.* Metascape provides a biologist-oriented resource for the analysis of systems-level datasets. *Nature Communications*. 2019; **10**: 1523. doi: 10.1038/s41467-019-09234-6

17.	Cardoso-Moreira M, Halbert J, Valloton D *et al.* Gene expression across mammalian organ development. *Nature*. 2019; **571**: 505-509. doi: 10.1038/s41586-019-1338-5

18.	Bray NL, Pimentel H, Melsted P *et al.* Near-optimal probabilistic RNA-seq quantification. *Nature Biotechnology*. 2016; **34**: 525. doi: 10.1038/nbt.3519

19.	Brawand D, Soumillon M, Necsulea A *et al.* The evolution of gene expression levels in mammalian organs. *Nature*. 2011; **478**: 343-348. doi: 10.1038/nature10532

20.	Rehm HL, Berg JS, Brooks LD *et al.* ClinGen--the Clinical Genome Resource. *The New England Journal of Medicine*. 2015; **372**: 2235-2242. doi: 10.1056/NEJMsr1406261

21.	Martin AR, Williams E, Foulger RE *et al.* PanelApp crowdsources expert knowledge to establish consensus diagnostic gene panels. *Nature Genetics*. 2019; **51**: 1560-1565. doi: 10.1038/s41588-019-0528-2

22.	Stenson PD, Mort M, Ball EV *et al.* The Human Gene Mutation Database (HGMD((R))): optimizing its use in a clinical diagnostic or research setting. *Human Genetics*. 2020; **139**: 1197-1207. doi: 10.1007/s00439-020-02199-3

23.	Cardoso-Moreira M, Sarropoulos I, Velten B *et al.* Developmental Gene Expression Differences between Humans and Mammalian Models. *Cell Reports*. 2020; **33**: 108308. doi: 10.1016/j.celrep.2020.108308

24.	Gao JJ, Aksoy BA, Dogrusoz U *et al.* Integrative Analysis of Complex Cancer Genomics and Clinical Profiles Using the cBioPortal. *Science Signaling*. 2013; **6**: pl1. doi: 10.1126/scisignal.2004088

25.	Sondka Z, Dhir NB, Carvalho-Silva D *et al.* COSMIC: a curated database of somatic variants and clinical data for cancer. *Nucleic Acids Research*. 2024; **52**: D1210-D1217. doi: 10.1093/nar/gkad986

26.	Sonoda Y, Ozawa T, Hirose Y *et al.* Formation of intracranial tumors by genetically modified human astrocytes defines four pathways critical in the development of human anaplastic astrocytoma. *Cancer Research*. 2001; **61**: 4956-4960.

27.	Bolger AM, Lohse M, Usadel B. Trimmomatic: a flexible trimmer for Illumina sequence data. *Bioinformatics*. 2014; **30**: 2114-2120. doi: 10.1093/bioinformatics/btu170

28.	Dobin A, Davis CA, Schlesinger F *et al.* STAR: ultrafast universal RNA-seq aligner. *Bioinformatics*. 2013; **29**: 15-21. doi: 10.1093/bioinformatics/bts635

29.	Li B, Dewey CN. RSEM: accurate transcript quantification from RNA-Seq data with or without a reference genome. *BMC Bioinformatics*. 2011; **12**: 323. doi: 10.1186/1471-2105-12-323

30.	Love MI, Huber W, Anders S. Moderated estimation of fold change and dispersion for RNA-seq data with DESeq2. *Genome Biology*. 2014; **15**: 550. doi: 10.1186/s13059-014-0550-8

31.	Kuzmin E, VanderSluis B, Ba ANN *et al.* Exploring whole-genome duplicate gene retention with complex genetic interaction analysis. *Science*. 2020; **368**: eaaz5667. doi: 10.1126/science.aaz5667

32.	Katoh K, Standley DM. MAFFT multiple sequence alignment software version 7: improvements in performance and usability. *Molecular Biology and Evolution*. 2013; **30**: 772-780. doi: 10.1093/molbev/mst010

33.	Suyama M, Torrents D, Bork P. PAL2NAL: robust conversion of protein sequence alignments into the corresponding codon alignments. *Nucleic Acids Research*. 2006; **34**: W609-612. doi: 10.1093/nar/gkl315

34.	Yang Z. PAML 4: phylogenetic analysis by maximum likelihood. *Molecular Biology and Evolution*. 2007; **24**: 1586-1591. doi: 10.1093/molbev/msm088

35.	Blum M, Andreeva A, Florentino LC *et al.* InterPro: the protein sequence classification resource in 2025. *Nucleic Acids Research*. 2024; **53**: D444-D456. doi: 10.1093/nar/gkae1082

36.	Shao Y, Chen CY, Shen H *et al.* GenTree, an integrated resource for analyzing the evolution and function of primate-specific coding genes. *Genome Research*. 2019; **29**: 682-696. doi: 10.1101/gr.238733.118

37.	Singh PP, Isambert H. OHNOLOGS v2: a comprehensive resource for the genes retained from whole genome duplication in vertebrates. *Nucleic Acids Research*. 2020; **48**: D724-D730. doi: 10.1093/nar/gkz909

38.	Yanai I, Benjamin H, Shmoish M *et al.* Genome-wide midrange transcription profiles reveal expression level relationships in human tissue specification. *Bioinformatics*. 2005; **21**: 650-659. doi: 10.1093/bioinformatics/bti042

39.	Altschul SF, Gish W, Miller W *et al.* Basic Local Alignment Search Tool. *Journal of Molecular Biology*. 1990; **215**: 403-410. doi: DOI 10.1006/jmbi.1990.9999

40.	Liaw A. Classification and regression by randomForest. *R news*. 2002.

41.	Dickinson ME, Flenniken AM, Ji X *et al.* High-throughput discovery of novel developmental phenotypes. *Nature*. 2016; **537**: 508-514. doi: 10.1038/nature19356

42.	Yu D, Ren Y, Uesaka M *et al.* Hagfish genome elucidates vertebrate whole-genome duplication events and their evolutionary consequences. *Nature Ecology & Evolution*. 2024; **8**: 519-535. doi: 10.1038/s41559-023-02299-z

43.	Rosikiewicz W, Kabza M, Kosinski JG *et al.* RetrogeneDB-a database of plant and animal retrocopies. *Database-Oxford*. 2017; **2017**: bax038. doi: 10.1093/database/bax038

44.	Uhlen M, Fagerberg L, Hallström BM *et al.* Tissue-based map of the human proteome. *Science*. 2015; **347**: 1260419. doi: 10.1126/science.1260419

45.	Murat F, Mbengue N, Winge SB *et al.* The molecular evolution of spermatogenesis across mammals. *Nature*. 2023; **613**: 308-316. doi: 10.1038/s41586-022-05547-7

46.	Ciomborowska J, Rosikiewicz W, Szklarczyk D *et al.* "Orphan" retrogenes in the human genome. *Mol Biol Evol*. 2013; **30**: 384-396. doi: 10.1093/molbev/mss235

47.	Flicek P, Amode MR, Barrell D *et al.* Ensembl 2012. *Nucleic Acids Research*. 2012; **40**: D84-D90.

48.	Rhead B, Karolchik D, Kuhn RM *et al.* The UCSC Genome Browser database: update 2010. *Nucleic Acids Research*. 2010; **38**: D613-619. doi: 10.1093/nar/gkp939

49.	Doench JG, Hartenian E, Graham DB *et al.* Rational design of highly active sgRNAs for CRISPR-Cas9-mediated gene inactivation. *Nature Biotechnology*. 2014; **32**: 1262-1267. doi: 10.1038/nbt.3026

50.	Yang H, He BZ, Ma H *et al.* Expression profile and gene age jointly shaped the genome-wide distribution of premature termination codons in a Drosophila melanogaster population. *Mol Biol Evol*. 2015; **32**: 216-228. doi: 10.1093/molbev/msu299

51.	Hsu PD, Scott DA, Weinstein JA *et al.* DNA targeting specificity of RNA-guided Cas9 nucleases. *Nature Biotechnology*. 2013; **31**: 827-832. doi: 10.1038/nbt.2647

52.	Tsai SQ, Zheng Z, Nguyen NT *et al.* GUIDE-seq enables genome-wide profiling of off-target cleavage by CRISPR-Cas nucleases. *Nature Biotechnology*. 2015; **33**: 187-197. doi: 10.1038/nbt.3117

53.	Langmead B, Trapnell C, Pop M *et al.* Ultrafast and memory-efficient alignment of short DNA sequences to the human genome. *Genome Biology*. 2009; **10**: R25. doi: gb-2009-10-3-r25 [pii]

10.1186/gb-2009-10-3-r25

54.	Doench JG, Fusi N, Sullender M *et al.* Optimized sgRNA design to maximize activity and minimize off-target effects of CRISPR-Cas9. *Nature Biotechnology*. 2016; **34**: 184-191. doi: 10.1038/nbt.3437

55.	Xu H, Xiao TF, Chen CH *et al.* Sequence determinants of improved CRISPR sgRNA design. *Genome Research*. 2015; **25**: 1147-1157. doi: 10.1101/gr.191452.115

56.	Parrish PCR, Thomas JD, Gabel AM *et al.* Discovery of synthetic lethal and tumor suppressor paralog pairs in the human genome. *Cell Rep*. 2021; **36**: 109597. doi: 10.1016/j.celrep.2021.109597

57.	Hart T, Chandrashekhar M, Aregger M *et al.* High-Resolution CRISPR Screens Reveal Fitness Genes and Genotype-Specific Cancer Liabilities. *Cell*. 2015; **163**: 1515-1526. doi: 10.1016/j.cell.2015.11.015

58.	Najm FJ, Strand C, Donovan KF *et al.* Orthologous CRISPR-Cas9 enzymes for combinatorial genetic screens. *Nature Biotechnology*. 2018; **36**: 179-189. doi: 10.1038/nbt.4048

59.	McDonald ER, 3rd, de Weck A, Schlabach MR *et al.* Project DRIVE: A Compendium of Cancer Dependencies and Synthetic Lethal Relationships Uncovered by Large-Scale, Deep RNAi Screening. *Cell*. 2017; **170**: 577-592.e510. doi: 10.1016/j.cell.2017.07.005

60.	Han K, Jeng EE, Hess GT *et al.* Synergistic drug combinations for cancer identified in a CRISPR screen for pairwise genetic interactions. *Nature Biotechnology*. 2017; **35**: 463-474. doi: 10.1038/nbt.3834

61.	D'Antonio M, Guerra RF, Cereda M *et al.* Recessive Cancer Genes Engage in Negative Genetic Interactions with Their Functional Paralogs. *Cell Reports*. 2013; **5**: 1519-1526. doi: 10.1016/j.celrep.2013.11.033

62.	Makino T, McLysaght A. Ohnologs in the human genome are dosage balanced and frequently associated with disease. *P Natl Acad Sci USA*. 2010; **107**: 9270-9274. doi: 10.1073/pnas.0914697107

63.	Shen JP, Zhao DX, Sasik R *et al.* Combinatorial CRISPR-Cas9 screens for de novo mapping of genetic interactions. *Nat Methods*. 2017; **14**: 573-576. doi: 10.1038/nmeth.4225

64.	Shalem O, Sanjana NE, Hartenian E *et al.* Genome-scale CRISPR-Cas9 knockout screening in human cells. *Science*. 2014; **343**: 84-87. doi: 10.1126/science.1247005

65.	Meyers RM, Bryan JG, McFarland JM *et al.* Computational correction of copy number effect improves specificity of CRISPR-Cas9 essentiality screens in cancer cells. *Nat Genet*. 2017; **49**: 1779-1784. doi: 10.1038/ng.3984

66.	Kampmann M, Bassik MC, Weissman JS. Integrated platform for genome-wide screening and construction of high-density genetic interaction maps in mammalian cells. *P Natl Acad Sci USA*. 2013; **110**: E2317-E2326. doi: 10.1073/pnas.1307002110

67.	Li H, Durbin R. Fast and accurate short read alignment with Burrows-Wheeler transform. *Bioinformatics*. 2009; **25**: 1754-1760. doi: 10.1093/bioinformatics/btp324

68.	Hanna RE, Doench JG. A case of mistaken identity. *Nature Biotechnology*. 2018; **36**: 802-804. doi: 10.1038/Nbt.4208

69.	Borenstein M, Hedges LV, Higgins JPT *et al.* A basic introduction to fixed-effect and random-effects models for meta-analysis. *Res Synth Methods*. 2010; **1**: 97-111. doi: 10.1002/jrsm.12

70.	Love MI, Huber W, Anders S. Moderated estimation of fold change and dispersion for RNA-seq data with DESeq2. *Genome Biol*. 2014; **15**. doi: 10.1186/s13059-014-0550-8

71.	Brinkman EK, Chen T, Amendola M *et al.* Easy quantitative assessment of genome editing by sequence trace decomposition. *Nucleic Acids Research*. 2014; **42**. doi: 10.1093/nar/gku936

72.	Gonatopoulos-Pournatzis T, Aregger M, Brown KR *et al.* Genetic interaction mapping and exon-resolution functional genomics with a hybrid Cas9-Cas12a platform. *Nature Biotechnology*. 2020; **38**. doi: 10.1038/s41587-020-0437-z

73.	Imkeller K, Ambrosi G, Boutros M *et al.* gscreend: modelling asymmetric count ratios in CRISPR screens to decrease experiment size and improve phenotype detection. *Genome Biology*. 2020; **21**. doi: 10.1186/s13059-020-1939-1

74.	Wang T, Birsoy K, Hughes NW *et al.* Identification and characterization of essential genes in the human genome. *Science*. 2015; **350**: 1096-1101. doi: 10.1126/science.aac7041

75.	Anders S, Huber W. Differential expression analysis for sequence count data. *Genome Biology*. 2010; **11**: R106. doi: 10.1186/gb-2010-11-10-r106

76.	Esmaeili Anvar N, Lin C, Ma X *et al.* Efficient gene knockout and genetic interaction screening using the in4mer CRISPR/Cas12a multiplex knockout platform. *Nature Communications*. 2024; **15**: 3577. doi: 10.1038/s41467-024-47795-3

Supplementary Note Figures

 **Supplementary Note Figure. 1 The pipeline schema for designing sgRNAs.**

**a** Design of gene-targeting sgRNAs. **b-c** Design of the two control sgRNA groups, the intergenic region targeting group (panel **b**) and the non-targeting group (panel **c**). The chromosome icon was designed by Jason D. Rowley (<https://thenounproject.com/>).

 **Supplementary Note** **Figure. 2 Validation of three negative GI pairs by cell titer experiments.** **a** Growth dynamics of cells. RLU refers to relative light unit. **b** Relative fitness of three types of knock-out. The digenic knock-out is colored in purple. The error bar indicates one standard deviation. *P* values come from Student’s t test (two-sided).

**Supplementary Note Figure. 3 Interrogation of 17 candidate negative GI pairs with Cell Counting Kit-8 (CCK8) assay.** **a** Nine pairs called as negative GIs in the screen. **b** Eight pairs called as non-negative GIs in the screening. Validation results inconsistent with the screening results are marked in the blue box. The error bar indicates one standard deviation. *P* values come from Student’s t test (two-sided).

**Supplementary Note Figure. 4 Quality evaluation of the dual knock-out system.** **a** The exponential fit between the standard deviation and T0 read counts. The formula is listed. **b** Correlation of the gene-level dependency score (calculated by the variance weighted method) between biological replicates. The color gradient indicates the data density. Pearson’s *r* was calculated. **c** Non-targeting sgRNAs pairs grouped into 15 strata with the same number of sgRNAs in each stratum of HCT116 cell line. **d** Comparison of correlation coefficients across methods. Notably, the data shown in DESeq2 bar was obtained similarly to our exponential family fitting except that the DESeq2 package was used to analyze the log2-fold change of each sgRNA pair and generate the variance. **e** Distribution of dependency scores for essential and duplicate genes. **f** Reproducibility of dependency scores with the positions of sgRNA pairs reversed.
