# Supplementary material for: Moderate prevalence and evolutionary causes of paralogous compensation in humans
Source: Natl Sci Rev. 2026 Sep 3;13(19):nwag565. doi: 10.1093/nsr/nwag565 (PMC13715443; doi:10.1093/nsr/nwag565)
Supplement: Supplementary file 1 [file nwag565_supplemental_files.zip › Supplementary Note Figures/Supplementary Note Figure1.pdf]

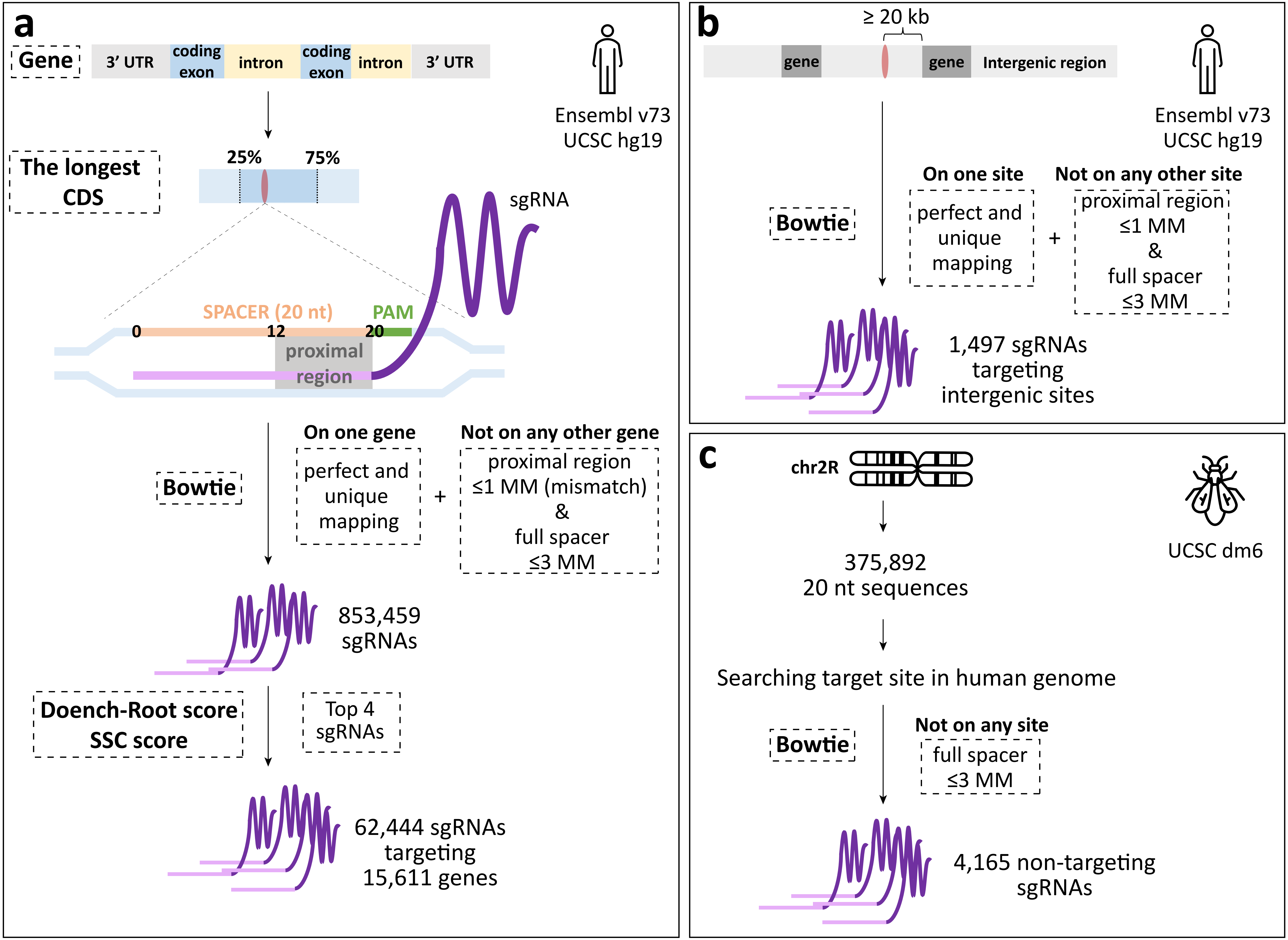
a
Gene
3′ UTR
coding exon
intron
coding exon
intron
3′ UTR
Ensembl v73
UCSC hg19
The longest CDS
25%
75%
sgRNA
SPACER (20 nt)
PAM
0
12
20
proximal region
Bowtie
On one gene
perfect and unique mapping
+
Not on any other gene
proximal region ≤1 MM (mismatch) & full spacer ≤3 MM
853,459 sgRNAs
Doench-Root score
SSC score
Top 4 sgRNAs
62,444 sgRNAs targeting 15,611 genes
b
≥ 20 kb
gene
gene
Intergenic region
Ensembl v73
UCSC hg19
Bowtie
On one site
perfect and unique mapping
+
Not on any other site
proximal region ≤1 MM & full spacer ≤3 MM
1,497 sgRNAs targeting intergenic sites
c
chr2R
UCSC dm6
375,892 20 nt sequences
Searching target site in human genome
Bowtie
Not on any site
full spacer ≤3 MM
4,165 non-targeting sgRNAs
