# Supplementary material for: Moderate prevalence and evolutionary causes of paralogous compensation in humans
Source: Natl Sci Rev. 2026 Sep 3;13(19):nwag565. doi: 10.1093/nsr/nwag565 (PMC13715443; doi:10.1093/nsr/nwag565)
Supplement: Supplementary file 1 [file nwag565_supplemental_files.zip › Supplementary Note Figures/Supplementary Note Figure2.pdf]

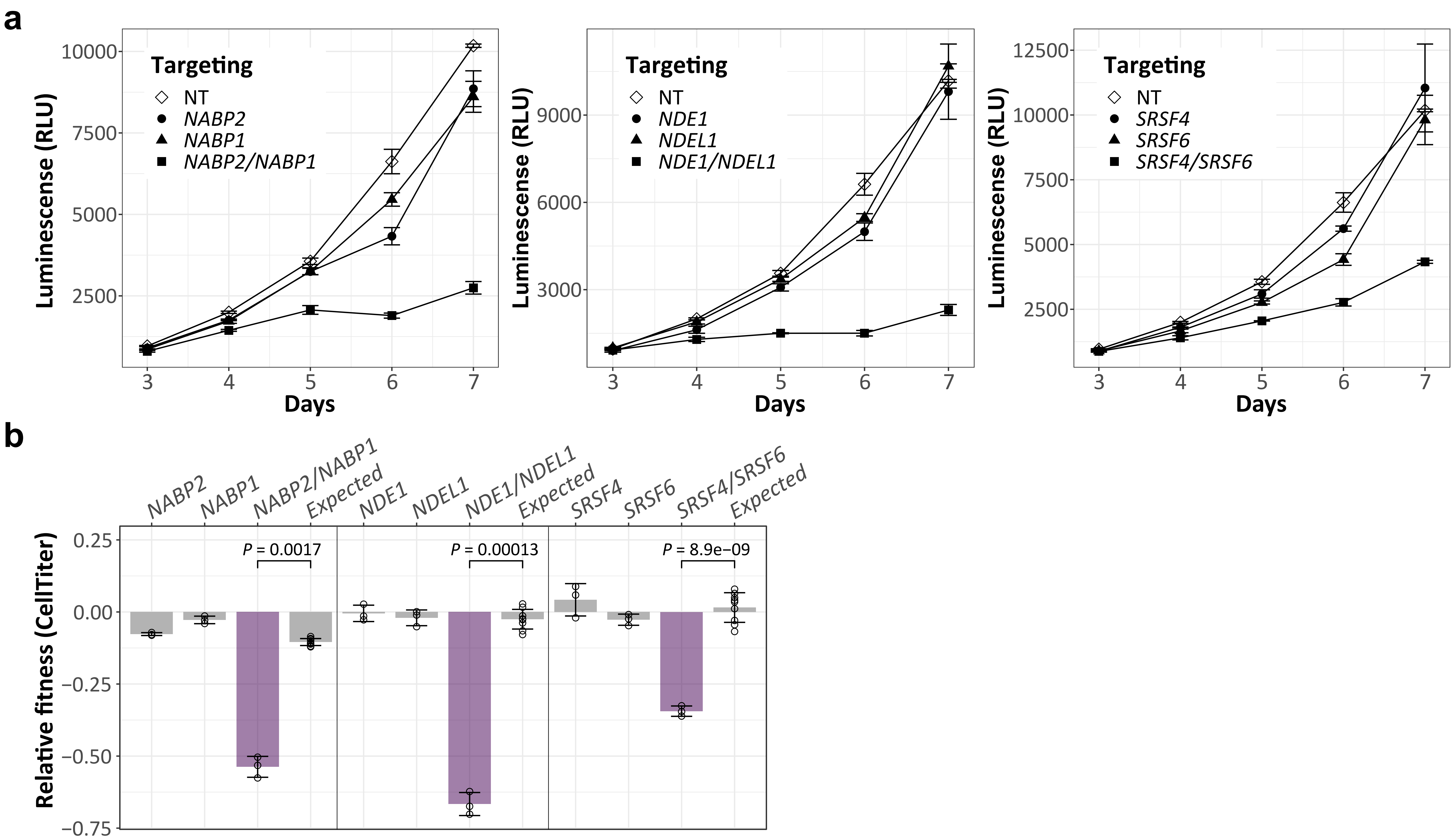

a
Targeting
NT
NABP2
NABP1
NABP2/NABP1
Luminescense (RLU)
Days
NDE1
NDEL1
NDE1/NDEL1
SRSF4
SRSF6
SRSF4/SRSF6
b
Expected
Relative fitness (CellTiter)
P = 0.0017
P = 0.00013
P = 8.9e−09
