# Supplementary material for: Moderate prevalence and evolutionary causes of paralogous compensation in humans
Source: Natl Sci Rev. 2026 Sep 3;13(19):nwag565. doi: 10.1093/nsr/nwag565 (PMC13715443; doi:10.1093/nsr/nwag565)
Supplement: Supplementary file 1 [file nwag565_supplemental_files.zip › Supplementary Note Figures/Supplementary Note Figure3.pdf]

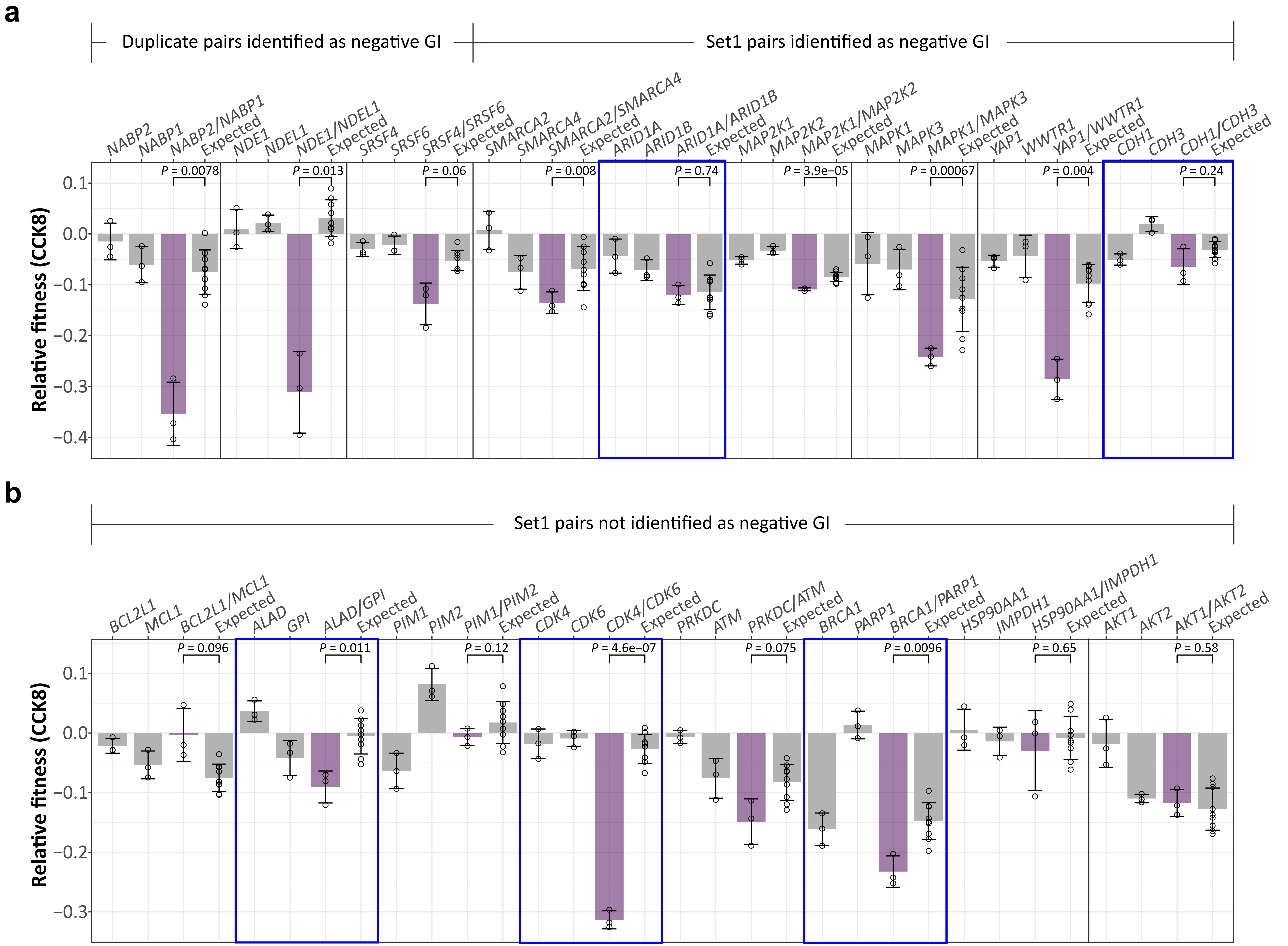
a
Duplicate pairs identified as negative GI
Set1 pairs idientified as negative GI
NABP2
NABP1
NABP2/NABP1
Expected
NDE1
NDEL1
NDE1/NDEL1
Expected
SRSF4
SRSF6
SRSF4/SRSF6
Expected
SMARCA2
SMARCA4
SMARCA2/SMARCA4
Expected
ARID1A
ARID1B
ARID1A/ARID1B
Expected
MAP2K1
MAP2K2
MAP2K1/MAP2K2
Expected
MAPK1
MAPK3
MAPK1/MAPK3
Expected
YAP1
WWTR1
YAP1/WWTR1
Expected
CDH1
CDH3
CDH1/CDH3
Expected
P = 0.0078
P = 0.013
P = 0.06
P = 0.008
P = 0.74
P = 3.9e−05
P = 0.00067
P = 0.004
P = 0.24
Relative fitness (CCK8)
b
Set1 pairs not idientified as negative GI
BCL2L1
MCL1
BCL2L1/MCL1
Expected
ALAD
GPI
ALAD/GPI
Expected
PIM1
PIM2
PIM1/PIM2
Expected
CDK4
CDK6
CDK4/CDK6
Expected
PRKDC
ATM
PRKDC/ATM
Expected
BRCA1
PARP1
BRCA1/PARP1
Expected
HSP90AA1
IMPDH1
HSP90AA1/IMPDH1
Expected
AKT1
AKT2
AKT1/AKT2
Expected
P = 0.096
P = 0.011
P = 0.12
P = 4.6e−07
P = 0.075
P = 0.0096
P = 0.65
P = 0.58
Relative fitness (CCK8)
