# Supplementary material for: Moderate prevalence and evolutionary causes of paralogous compensation in humans
Source: Natl Sci Rev. 2026 Sep 3;13(19):nwag565. doi: 10.1093/nsr/nwag565 (PMC13715443; doi:10.1093/nsr/nwag565)
Supplement: Supplementary file 1 [file nwag565_supplemental_files.zip › Supplementary Note Figures/Supplementary Note Figure4.pdf]

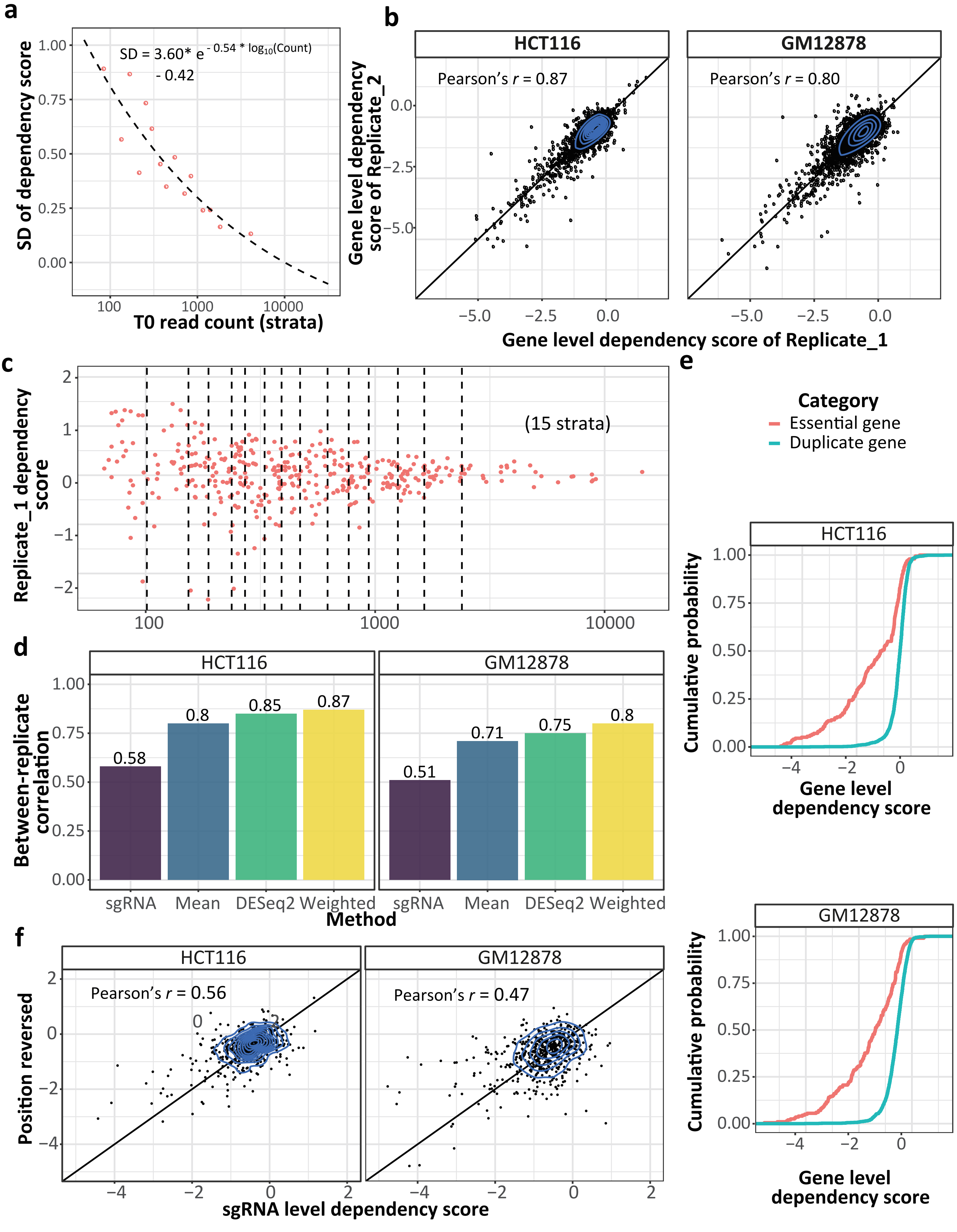

a
SD = 3.60* e - 0.54 * log10(Count) - 0.42
SD of dependency score
T0 read count (strata)
b
HCT116
GM12878
Pearson's r = 0.87
Pearson's r = 0.80
Gene level dependency score of Replicate_2
Gene level dependency score of Replicate_1
c
(15 strata)
Replicate_1 dependency score
d
HCT116
GM12878
0.58
0.8
0.85
0.87
0.51
0.71
0.75
0.8
Between-replicate correlation
sgRNA
Mean
DESeq2
Weighted
Method
e
Category
Essential gene
Duplicate gene
HCT116
GM12878
Cumulative probability
Gene level dependency score
f
HCT116
GM12878
Pearson's r = 0.56
Pearson's r = 0.47
Position reversed
sgRNA level dependency score
